# Discovery, Biosynthesis and Biological Activity of a Succinylated Myxochelin from the Myxobacterial Strain MSr12020

**DOI:** 10.3390/microorganisms10101959

**Published:** 2022-09-30

**Authors:** Dorothy A. Okoth, Joachim J. Hug, Ronald Garcia, Rolf Müller

**Affiliations:** 1 Helmholtz-Institute for Pharmaceutical Research Saarland (HIPS), Helmholtz Centre for Infection Research (HZI), Department of Microbial Natural Products, Campus E8 1, Saarland University, 66123 Saarbrücken, Germany; 2 Department of Pharmacy, Saarland University, 66123 Saarbrücken, Germany; 3 German Center for Infection Research (DZIF), Partner Site Hannover-Braunschweig, 38124 Braunschweig, Germany; 4 Helmholtz International Labs, Department of Microbial Natural Products, Campus E8 1, Saarland University, 66123 Saarbrücken, Germany; 5 Department of Chemistry, School of Physical and Biological Sciences, Main campus, Maseno University, Maseno P.O. Box 333-40105, Kenya

**Keywords:** myxochelin, myxobacteria, biosynthesis, natural products, secondary metabolites, succinylation, siderophore, succinyl-coenzyme A

## Abstract

Myxobacteria feature unique biological characteristics, including their capability to glide on the surface, undergo different multicellular developmental stages and produce structurally unique natural products such as the catecholate-type siderophores myxochelins A and B. Herein, we report the isolation, structure elucidation and a proposed biosynthesis of the new congener myxochelin B-succinate from the terrestrial myxobacterial strain MSr12020, featuring a succinyl decoration at its primary amine group. Myxochelin-B-succinate exhibited antibacterial growth inhibition and moderate cytotoxic activity against selected human cancer cell lines. This unique chemical modification of myxochelin B might provide interesting insights for future microbiological studies to understand the biological function and biosynthesis of secondary metabolite succinylation.

## 1. Introduction

Iron is an essential element for most microorganisms [1,2]. Despite the abundance of iron in the earth’s crust, it is not readily bioavailable in aerobic environments due to its low solubility. Therefore most bacteria produce low molecular weight iron scavenging secondary metabolites to obtain iron from their environment [3,4], which are defined as siderophores [5]. These small, high-affinity iron-chelating secondary metabolites can be categorized into the four chemical classes catecholate, phenolate, hydroxamate and carboxylate types of siderophores on the basis of the structural moieties involved in iron chelation, whereby hybrids thereof are also commonly described [4,6]. Siderophores are typically synthetized by non-ribosomal peptide synthetases (NRPSs) modular multienzymes [7], whereas a smaller fraction of siderophores is produced by pathways that are independent of NRPSs and polyketide synthases (PKSs) such as desferrioxamine [8,9] or quinolobactin [10].

Myxobacteria not only display exceptional biological characteristics such as a complex chemical communication systems, multicellular development stages and the capability to move in coordinated manner to prey on other microorganisms [11] but are also producers of chemically exceptional and bioactive secondary metabolites [12]. Two different chemical types of siderophores are known to date from myxobacteria: the hydroxamate-type nannochelins [13] and the catecholate-type hyalachelins [14] and myxochelins [15,16], of which the latter have been investigated thoroughly in the last three decades [17,18,19].

The myxochelins are produced by numerous myxobacterial strains including *Stigmatella aurantica* sg a15 [20], *Sorangium cellulosum* So ce56 [21], *Myxococcus xanthus* DK 1622 [22,23] and *Angiococcus disciformis* An d30 [15] to maintain their iron homeostasis since it is indispensable for microbial viability [20]. These myxobacterial siderophores have been occasionally described or associated with other bacteria such as the actinomycetes *Nonomuraea* sp. TP-A0861 [24] and *Steptomyces albicus* m-9-20 [25] or the *Chloroflexi* bacterium *Herpetosiphon aurantiacus* [26]. Recently a number of new myxochelin derivatives have been isolated from different myxobacterial strains in which the common 2,3-dihydroxybenzoic acid has been replaced by a nicotinic acid moiety [17] or a 4,5-dihydroimidazole moiety [27]. Due to the relative simple structure of the myxochelins, chemical synthetic efforts led not only to the diversification of the aromatic scaffold [17,28] but also to the generation of *hexadentate* siderophores termed myxochelin C–F [16]. In addition, more myxochelin derivatives have been generated biotechnologically by precursor-directed biosynthesis [29]. However, to the best of our knowledge, except for myxochelin C, none of the described naturally produced myxochelins feature a modification of the primary alcohol or amino group.

**Figure 1:**
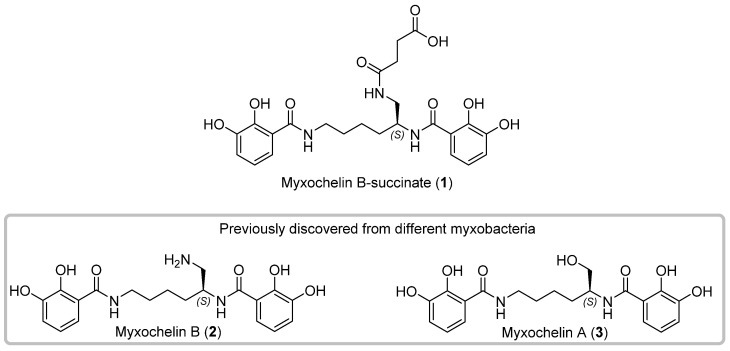
Chemical structures of the new myxochelin derivative myxochelin B-succinate (**1**), and the rediscovered congeners myxochelin B (**2**) and A (**3**) isolated from the myxobacterial strain MSr12020 (in grey box).

We hereby report the isolation, full structure elucidation and propose a biosynthetic pathway leading to the uniquely modified myxochelin congener myxochelin-B-succinate (**1**) from the myxobacterial strain MSr12020 along the re-isolated congeners myxochelins B (**2**) and A (**3**) (Figure 1). The genetic origin of **1**–**3** was identified by in silico analysis and the biosynthetic conversion from **2** to **1** was further probed by in vitro reactions with the highly reactive intracellular metabolite succinyl-coenzyme A (succinyl-CoA) in order to reveal the non-enzymatic succinylation leading to the formation of **1**.

## 2. Materials and Methods

### 2.1. Maintenance of Myxobacterial Cultures

The myxobacterial strain MSr12020 was cultivated in VY/2 medium [%, (*w*/*v*) 0.2 soytone (BD), 0.3 casitone (BD), 0.2 glucose (Sigma-Aldrich), 0.8 soluble starch (Roth), 0.15 Yeast extract (BD), 0.1 CaCl_2_ x 2H_2_O, 0.1 MgSO_4_ x 7H_2_O, 50 mM HEPES, 8 mg/L Fe-EDTA, pH adjusted to 7.2 with 10N KOH before autoclaving] containing 5% (*v*/*v*) cell inoculum and 2% (*v*/*v*) amberlite resin XAD-16 (Sigma) for 14 days at 160 rpm, 30 °C. At the end of fermentation, resin and cells were harvested together by centrifugation at 8000 rpm, 30 min, 4 °C.

### 2.2. Standardized HPLC–MS Conditions for Analysis of Secondary Metabolism of Crude Extracts

The broth extracts were analyzed by high-performance liquid chromatography–high-resolution electrospray ionization-diode array-detector–mass spectrometry (HPLC-HRESI-DAD-MS) on a maXis 4G mass spectrometer (Bruker Daltonics, Billerica, MA, USA) coupled with a Dionex UltiMate 3000 Rapid Separation (RS)LC system (Thermo Fisher Scientific, Waltham, MA, USA) using a BEH C18 column (100 × 2.1 mm, 1.7 μm) (Waters, Eschborn, Germany) with a gradient of 5–95% acetonitrile (ACN) + 0.1% formic acid (FA) in H_2_O + 0.1% FA at 0.6 mL/min and 45 °C over 18 min with ultraviolet (UV) detection by a diode array detector (DAD) at 200–600 nm. Mass spectra were acquired from 150 to 2000 *m*/*z* at 2 Hz. Detection was performed in the positive MS mode. The plugin for Chromeleon Xpress (Thermo Fisher Scientific, Waltham, MA, USA, version 6.8) was used for operation of the Dionex UltiMate 3000 RSLC system. HyStar (Bruker Daltonics, Billerica, MA, USA, version 3.2) was used to operate on the maXis 4G mass spectrometer system. HPLC-MS mass spectra were analyzed with DataAnalysis (Bruker Daltonics, Billerica, MA, USA, version 4.2).

In order to conduct statistical metabolome analysis to identify alternative producers of **1**–**3**, both the myxobacterial strain and medium blanks were cultivated and extracted in triplicates as described elsewhere [17]. Each crude extract was measured as technical duplicates yielding a total number of six replicates for the bacterial and medium blank extracts. T-ReX-3D molecular feature finder of MetaboScape 6.0.2 (Bruker Daltonics, Billerica, MA, USA) was used to obtain molecular features. Detection parameters were set to intensity threshold 5 × 10^3^ and minimum peak length of five spectra. Identification of bacterial features was performed with the built-in t-test routine and filtered to appearance in all six bacterial extracts and in none of medium blank extracts. The in-house standard extract database embedded in the software bundle Mxbase Explorer 3.2.27 was used for the search of alternative producers of **1**–**3**. The molecular formula and experimentally determined retention times of ions typically observed from the myxochelins were used as data input.

### 2.3. Isolation of ***1**–**3*** Via Liquid–Liquid Extraction, Flash Chromatography and Semi-Preparative HPLC

The myxobacterial strain MSr12020 was cultivated in 26 L bufVY/2 medium containing 5% (*v*/*v*) cell inoculum and 2% (*v*/*v*) amberlite resin XAD-16 for 14 days at 160 rpm, 30 °C. At the end of fermentation, wet cell mass and adsorber resin XAD-16 were harvested together by centrifugation at 8000 rpm, 30 min and 4 °C. The crude extract was obtained from the fermentation broth via liquid acetone extraction; afterwards the acetone extract was dried under vacuum. The dried acetone extract (10.4 g) was then partitioned between methanol (MeOH) and *n*-hexane to remove fats. The MeOH layer was dried under vacuum to yield 6.6 g of extract. This extract was again sequentially partitioned in H_2_O and chloroform (CHCl_3_) followed by ethyl acetate (EA) partitioning. The non-aqueous extracts were dried in vacuo while the water portion was freeze-dried by lyophilization to yield a CHCl_3_ (2.86 g), EA (1.94 g) and H_2_O (1.8 g) extract. Each liquid–liquid extraction fraction was monitored for the presence of **1**–**3** via HPLC-MS as described above. Myxochelins **1**–**3** were detected in the EA and H_2_O residue.

The EA and H_2_O extracts revealed similar HPLC-MS profiles and were combined after evaporation of solvents. The extract was initially separated on a flash chromatography on an Isolera™One (Biotage, Uppsala, Sweden) with a SNAP 100 g column packed with C18-Reverse phase silica gel (70 Å, 200–400 mesh, 40–75 μm) using H_2_O (0.1% FA) as solvent **A**, ACN (0.1% FA) as solvent **B**, and acetone (0.1% FA) as solvent **C**. The flow rate was 50 mL/min, UV/VIS absorption was set at 250 and 312 nm. Collected fractions (45 mL) were monitored on a Dionex UltiMate 3000 RSLC system (Thermo Fisher Scientific, Waltham, MA, USA) coupled to an amaZon ion trap MS (Bruker Daltonics, Billerica, MA, USA). The elution gradient consisted of an initial isocratic mixture of 95:5% (H_2_O:ACN) for five column volumes (CVs), then ramped to 70:30% (H_2_O:ACN) for 10 CV. The gradient was held at 70:30 (H_2_O:ACN) for five CVs before being raised again to 5:95% (H_2_O:ACN) for 25 CVs. This was followed by an isocratic solvent system 5:95% (H_2_O:ACN) for five CVs. Similar fractions, based on mass profiles were pooled together. Fractions 38–42 and 55–63 contained the molecular masses of interest and were dried under vacuum to yield 76 mg and 43.2 mg respectively.

The flash chromatography fractions 38–42 and 55–63 were purified on UltiMate 3000 semi-preparative system coupled to a Thermo Scientific Dionex UltiMate 3000 Series automated fraction collector (Bruker Daltonics, Billerica, MA, USA) using a XSelect CSH C_18_ Prep column, 5 μm, 10 × 250 mm (Waters TM) and eluted with H_2_O (0.1% FA) and ACN (0.1% FA). The fractions were monitored by mass spectrometry and by using the UV/VIS detector set at 220, 250, 312, and 400 nm. The gradient program was adjusted to an initial isocratic gradient 95:5% (H_2_O:ACN) for 3 min followed by gradient ramp to 16:84 (H_2_O:ACN) in 5 min. The gradient was then raised to 17:83% (H_2_O:ACN) for 23 min and then raised again to 5:95% (H_2_O:ACN) in 5 min and held for 2 min before lowering the gradient back to 95:5% (H_2_O:ACN) in 1 min. The column was re-equilibrated for 5 min using 95:5% (H_2_O:ACN). Compound **2** and **3** were detected using mass spectrometry on the Agilent 1100 series (Agilent Technologies, Santa Clara, CA, USA) coupled to the HCT 3D ion trap (Bruker Daltonics, Billerica, MA, USA) or with a UV detector on the Dionex UltiMate 3000 RSLC system by UV absorption at 220, 250, 312, and 400 nm. Fraction 38–42 yielded compound **2** at a retention time of 10 min while fraction 55–63 led to isolation of compounds **1** and **3** at retention times 16 min and 18 min respectively. The HPLC fractions were dried under N_2_ yielding compound **1** (7.4 mg), **2** (12.2 mg) and **3** (4.9 mg).

The identity and chemical purity of **1**–**3** was confirmed and monitored before NMR analysis via HPLC-HRESI-DAD-MS as described above (2.2), with the exception that the gradient of 5–95% (H_2_O–ACN) + 0.1% FA) at 0.6 mL/min and 45 °C was conducted over 9 min (termed “Short standardized HPLC–MS condition”).

Myxochelin-B-succinate (**1**): pale brown paste; ${\left[\alpha \right]}_{D}^{25}$ −12.2 (*c* 0.5, MeOH), UV (MeOH) λ_max_ nm (log ε): 210 (4.82), 248 (4.4), 312 (2.89) nm; ^1^H and ^13^C NMR, Table S1; HR–ESITOFMS (*m*/*z*): [M+H]^+^ calcd for C_24_H_30_N_3_O_9_, 504.1977; found 504.1976; Δ 0.2 ppm, retention time 5.52 min (according to standardized HPLC–MS conditions in 2.2).

Mxochelin B (**2**): pale brown powder; ${\left[\alpha \right]}_{D}^{25}$ −9.2 (*c* 0.5, MeOH), UV (MeOH) λ_max_ nm (log ε): 208(4.21) 246 (4.4), 312 (2.89) nm; ^1^H and ^13^C NMR, Table S2; HR–ESITOFMS (*m*/*z*): [M+H]^+^ calcd for C_20_H_26_N_3_O_6_, 404.1817; found, 404.1819; Δ 0.5 ppm, retention time 4.00 min (according to standardized HPLC–MS conditions in 2.2).

Myxochelin A (**3**): pale brown powder; ${\left[\alpha \right]}_{D}^{25}$ −8.6 (*c* 0.5, MeOH), UV (MeOH) λ_max_ nm (log ε): 210 (4.35) (4.21) 248 (4.4), 312 (2.89) nm; ^1^H and ^13^C NMR, Table S3; HR–ESITOFMS (*m*/*z*): [M+H]^+^ calcd for C_20_H_25_N_2_O_7_, 405.1657; found 405.1658; Δ 0.2 ppm, retention time 5.49 min (according to standardized HPLC–MS conditions in 2.2).

### 2.4. NMR Based Structure Elucidation and Chiroptical Measurement

The chemical structures of **1**–**3** were determined via multidimensional NMR analysis. ^1^H-NMR, ^13^C-NMR and 2D spectra were recorded at 500 MHz (1H)/175 MHz (^13^C), conducting an Ascend 500 spectrometer using a cryogenically cooled triple resonance probe (Bruker Biospin, Rheinstetten, Germany). Samples were dissolved in CD_3_OD. Chemical shifts are reported in ppm relative to tetramethylsilane; the solvent was used as the internal standard (Supplementary Materials, Figures S16–S61).

Chiroptical rotation of **1**–**3** was measured in MeOH using the polarimeter model 341 (PerkinElmer Inc., Waltham, MA, USA) in a 50 mm × 2 mm cell at 25 °C (${\left[\alpha \right]}_{D}^{20}$). The sample solution concentration was 0.5 mg/mL. Circular dichroism measurements were performed for **1** at 0.5 mg/mL in MeOH (190–400 nm) with the J-1500 CD spectrophotometer (JASCO, Easton, MD, USA).

### 2.5. Bioactivity Profiling

Antimicrobial activity was determined using agar diffusion assay [30] paralleling previous bioactivity investigations of different myxochelins [15,31]. Single colonies of *Staphylococcus aureus*, *Acinetobacter baumannii* and *Candida albicans* were picked and inoculated into 8 mL TSB liquid medium, respectively. The overnight culture at 37 °C were diluted to 1 × 10^7^ cells/mL with TSB liquid medium. The respective compound was dissolved in MeOH (5 mg/mL), and 5 μL of the solution was applied on a paper disk (Cytiva Whatman^®^ Antibiotic Assay Discs, 6 mm diameter). The disks were then placed onto an agar plate containing a soft agar overlay of the test microorganisms. Kanamycin (antimicrobial standard agent against Gram-negative bacterial microorganisms), fusidic acid (antimicrobial standard agent against Gram-positive bacterial microorganisms) and cycloheximide (antimicrobial standard agent against Gram-positive fungi) at a concentration of 5 mg/mL were used as positive controls, and the solvent MeOH or dimethyl sulfoxide (DMSO) as the negative control. After incubation at 37 °C for 18 h, growth inhibition zones (in mm) were recorded as antimicrobial activity.

Carcinoma cell line HCT-116, DSMZ No. ACC 581, KB-3-1 (cervix carcinoma cell line, DSMZ No. ACC 158) and U2OS (human bone osteosarcoma epithelial cells) were cultured in RPMI 1640 medium supplemented with 10% fetal bovine serum 100 U/mL penicillin and 100 μg/mL streptomycin, respectively. Media and supplements were purchased from Sigma-Aldrich (St. Louis, MO, USA). The cells were incubated in 5% CO_2_ at 37 °C until they reached approximately 50−70% confluence, and then treated with various concentrations of compounds dissolved in water. DMSO was used as the negative control. An MTT [(3-(4,5-dimethylthiazol-2-yl)-2,5-diphenyl-2H-tetrazolium bromide), Sigma, St. Louis, MO, USA] assay was used to measure the proliferation of cells (6 x 10^3^ cells) treated with different compounds in 96-well plates. After 24 h treatment with DMSO, different concentrations of tested compounds, as well as the positive control doxorubicin in DMSO, the cells were incubated with 10 μL of MTT^32^ (5 mg/mL) for 4 h at 37 °C. The medium was discarded, and cells were washed with 100 μL PBS before adding 100 μL isopropanol/10 N HCl (250:1) in order to dissolve formazan granules. The absorbance at 570 nm was measured using a microplate reader (Tecan Infinite M200Pro). Cell viability was expressed as percentage relative to the respective DMSO control. The IC_50_ values were determined by sigmoidal curve fitting using GraphPad PRISM 8 (GraphPad Software, San Diego, CA, USA). All bioactivity experiments were conducted as triplicates.

### 2.6. In Vitro Succinylation Reactions of ***2*** and ***3***

Non-enzymatic succinylation reactions of **2** and **3** were tested in a reaction mixture (100 µL volume) containing 1 μM **2** or **3** and 100 μM succinyl-CoA (Sigma-Aldrich (St. Louis, MO, USA); succinyl coenzyme A sodium salt, CAS: 108347-97-3) or succinate acid (Sigma-Aldrich (St. Louis, MO, USA); succinic acid disodium salt, CAS: 150-90-3) in DPBS buffer (Dulbecco’s Phosphate Buffered Saline, (Sigma-Aldrich (St. Louis, MO, USA). The experimental setting of the performed in vitro reactions with regard to intracellular concentration of succinyl CoA in (myxo)bacteria, approximately resembles the value of ~ 230 µM found in *E. coli* [32]. Therefore, the performed non-enzymatic succinylation reactions are applicable to the physiological bacterial environment regarding the chosen concentration of succinyl CoA and ratio to its substrate. Due to the instability of succinyl-CoA in aqueous solution [33], the freshly prepared stock solution (10 mM) was immediately used for in vitro reactions. The pH and ionic strength of the DPBS buffer was adjusted by KOH and NaCl, respectively (pH 5.5/7.2/10.0). The reaction was carried out for 2.5 h at 30 °C in a 1.5 mL microcentrifuge tube. Negative control testing were performed by omitting succinyl-CoA or succinic acid. The mixture was subsequently transferred for centrifugation at 13,000× *g* for 15 min at 4 °C (VWR centrifuge ECN521-3601, Hitachi Koki Co., Ltd., Tokyo, Japan) and the SN was subjected to HPLC-MS analysis as described above.

### 2.7. Applied Software, DNA Sequence Analysis, and Bioinformatics Methods

Genomic DNA isolation and sequencing of myxobacterial strain MSr12020—which belongs according to its 16S rRNA to a novel branch in a *Polyangiaceae* family and shows closest neighbor with *Polyangium* within the myxobacterial suborder Sorangiineae—has been described previously by Okoth et al. [34]. The MSr12020 genome was screened for secondary metabolite BGCs using the antiSMASH 6.0 [35] online tool and the software Geneious Prime^®^ (Biomatters Ltd., Auckland, New Zealand, 2020.0.5) [36]. The nucleotide or amino acid sequence of interest was aligned with the basic local alignment search tool (BLAST) against our in-house genome database or the publicly available nucleotide database, in order to find homologous genes or proteins. The functional prediction of ORFs was performed by either using protein blast and/or blastx programs and Pfam [37]. To obtain further information concerning the catalytic function of the identified biosynthetic proteins, the amino acid sequences were evaluated by the in silico protein homology analogy recognition engine 2 (Phyre2) [38]. Raw data from the alignments for in silico evaluation of the myxochelin biosynthetic proteins were stored on the in-house server. Sequence alignments were performed with embedded Geneious alignment software with the following setups:

Pairwise alignments (alignment type: global alignment with free end gaps; cost matrix: Blosum62; gap open penalty: 12; gap extension penalty: 3). Multiple alignments (alignment type: global alignment with free end gaps; cost matrix: Blosum45; gap open penalty: 12; gap extension penalty: 3; refinement iterations: 2).

The nucleotide sequence of the myxochelin BGC originating from MSr12020 has been deposited in GenBank and is accessible under the accession number OP359050. The same nucleotide sequence will be implemented in the Minimum Information about a Biosynthetic Gene cluster (MIBiG) database. Further information concerning gene sequences can be found in the Supplementary Information.

## 3. Results

### 3.1. Discovery, Isolation and Structural Elucidation of ***1***

**Figure 2:**
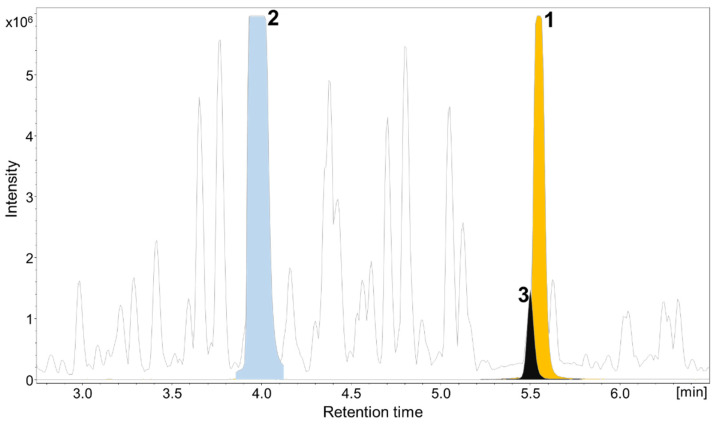
High-performance liquid chromatography–mass spectrometry base peak chromatogram (HPLC–MS BPC) (grey) and extracted ion chromatograms (EICs) of **1** orange ([M+H]^+^ 504.1976 *m*/*z*, (orange), **2** ([M+H]^+^ 404.1819 *m*/*z*, blue), and **3** ([M+H]^+^ 405.1658 *m*/*z*, black) from myxobacterial MSr12020 crude extract.

Cultivation of the myxobacterial strain MSr12020 was performed in VY/2 medium with supplementation of adsorber resin XAD-16. The secondary metabolome of MSr12020 revealed—according to our in-house LC–MS metabolome database termed Myxobase [39]—one previously uncharacterized myxochelin congener **1** and the known compounds **2** and **3** (Figure 2). A cultivation volume of 26 L containing bacterial cells and adsorber resin XAD-16 was extracted with acetone followed by liquid–liquid extraction to yield a semi-crude EA and H_2_O extract. Both extracts were combined and separated via flash chromatography and the fractions containing compounds **1**–**3** were further purified by semi-preparative HPLC. This resulted in compounds **1** (7.4 mg), **2** (12.2 mg) and **3** (4.9 mg).

Compound **1** was isolated as a brown solid with a molecular formula of C_24_H_30_N_3_O_9_ as observed in the high-resolution mass spectrum (HRMS). A molecular ion of [M+H]^+^ m/z of 504.1976 and *m*/*z* 1007.3858 [2M+H]^+^ was observed (calculated for 504.1977). The tandem MS (MS^2^) fragmentation was characterized by *m*/*z* 486.19 (C_24_H_28_N_3_O_8_^+^, [M-H_2_O+H]^+^), 444.21 (C_23_H_30_N_3_O_6_^+^), 404.19 (C_20_H_26_N_3_O_6_^+^, [M-succinyl+H]^+^), 387.15 (C_20_H_23_N_2_O_6_^+^, [M-succinyl-NH_2_+H]^+^), 386.18 (C_17_H_26_N_3_O_6_^+^, [M-dihdroxybenzoyl+H]^+^) due to loss of the dihydroxybenzoyl group), 350.17 (C_17_H_24_N_3_O_5_^+^, [M-H_2_O-dihydroxybenzoyl+H]^+^), 268.16 (C_13_H_22_N_3_O_3_^+^, [M-dihdroxybenzoyl-succinyl+H]^+^), 251.14 (C_13_H_19_N_2_O_3_^+^, [M-dihdroxybenzoyl-succinyl-NH_2_+H]^+^), 234.11 (C_13_H_16_NO_3_^+^), 214.15 (C_10_H_20_NO_2_^+^, [M-H_2_O-2×dihydroxybenzoyl+H]^+^). The UV-VIS absorption of **1** at λ_max_ 212, 250 and 312 nm corresponds to π-π* transition of benzene ring of the dihydroxybenzoyl group [40,41].

The ^1^H NMR spectrum showed six aromatic protons δ_H_ 6.92 (1H, *dd*, *J =* 1.45, 7.9 Hz, 4′-H), 6.90 (1H, *dd*, *J =* 1.45, 8.0 Hz, 4″-H), 6.70 (1H, *dd*, *J =* 7.9, 8.0 Hz, H-5′), 6.68 (1H, *dd*, *J =* 7.86, 8.0 Hz, H-5″) 7.18 (1H, *dd*, *J =* 1.45, 8.0 Hz, H-6′) and δ_H_ 7.21 (1H, *dd*, *J =* 1.45, 8.0 Hz, H-6″) whose coupling pattern and coupling constants suggested each ring had three neighboring protons in a 1,2,3 trisubstituted aromatic ring. The ^1^H NMR spectrum also showed the presence of seven methylene signals δ_H_ 3.44 (1H, *dd*, *J =* 4.85, 13.8 Hz, H-1), 3.38 (2H, *t*, *J =* 7.0 Hz, H-6), 3.29 (1H, *dd*, *J =* 7.3, 13.8 Hz, H-1), 2.55 (2H, *t*, *J =* 6.85 Hz, H-3‴) 2.44 (2H, *m*, H-2‴), 1.67 (2H, *m*, H-3 and 1H, H-5) and δ_H_ 1.48 (2H, *m*, 4-H) and one methine proton δ_H_ 4.21(1H, *m*, H-2).

The ^13^C NMR spectrum was characterized by one acid carbonyl δ_C_ 176.8 (C-4‴) and three amide carbonyls 175.5 (C-1‴), 171.8 (C-7″), 171.7 (C-7′), four oxygenated quartenary aromatic carbons δ_C_ 150.5 (C-2″), 150.4 (C-2′), 147.5 (C-3′), 147.4 (C-3″), six aromatic methines δ_C_ 119.8 (C-5′), 119.7 (C-4′ and C-4″), 119.7 (C-5″), 118.9 (C-6″), 118.7 (C-6′), and two quaternary carbons δ_C_ 116.9 (C-1″) and 116.9 (C-1′). In addition, the ^13^C NMR spectrum also indicated the presence of seven methylenes δ_C_ 44.1 (C-1), 40.4 (C-6), 32.6 (C-3), 31.9 (C-2‴), 30.7 (C-3‴), 30.3 (C-5), 24.6 (C-4) and one methine δ_C_ 51.2 (C-2) resonance. The presence of two dihydroxybenzamide groups was verified by ^1^H-^1^H COSY H-4′/H-5′ and H-5′/H-6′ and ^1^H-^13^C H-4′/C-1′,C-2′, C-3′, H-5′/C-1′, C-2′, C-3′, C-6′, C-7′, H-6′/C-1′, C-2′, C-3′, C-4′, C-5′ and C-7′ HMBC correlations. The occurrence of an aliphatic alkyl chain -CH_2_-CH_2_-CH_2_-CH-CH_2_-partial structure was deduced from the H-1/H-2, H-2/H-3, H-3/H-4, H-5/-H-6 ^1^H-^1^H correlations.

**Figure 3:**
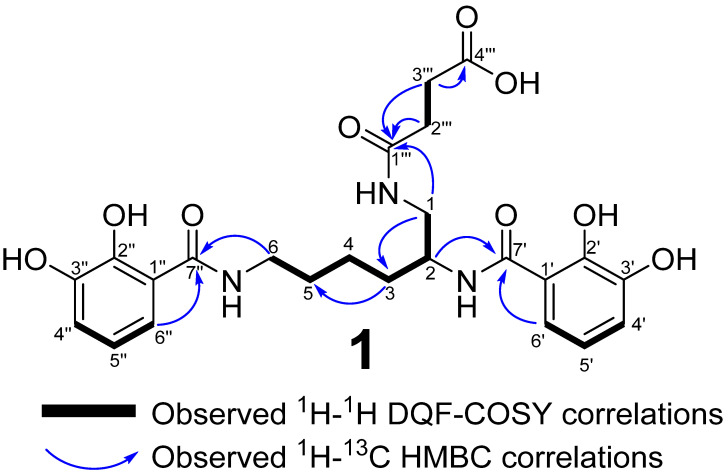
Carbon numbering, key ^1^H-^1^H COSY and ^1^H-^13^C HMBC correlations for **1**.

The observed ^1^H-^13^C HMBC correlations between the H-6 and C-7″, H-2 and H-C-7′ confirmed the chain being attached to the dihdroxybenzamide at C-7′ and C-7″. The remaining two methylene groups and two carbonyls were assigned to the succinyl amide, inferred from the H-2‴/H-3‴ COSY cross peaks and H-2‴/C-1‴, C-3‴ and C-4‴ and H-3‴/C-1‴and C-4‴ HMBC interactions. It was noted that the succinyl moiety was connected to the aliphatic chain at H-1 based on observed H-1/C-1‴ ^1^H-^13^C HMBC interactions (Figure 3). Thus **1** was elucidated as myxochelin-B-succinate based on the similarities with the previously reported siderophore **2**. The negative optical rotation and the positive cotton effect observed for **1** (Figure S12) were similar to those observed in naturally occurring and synthetic **2** and **3** -(2*S*)-isomer [16,17,24,28,42].

### 3.2. Bioactivity of ***1**–**3***

**Figure 4:**
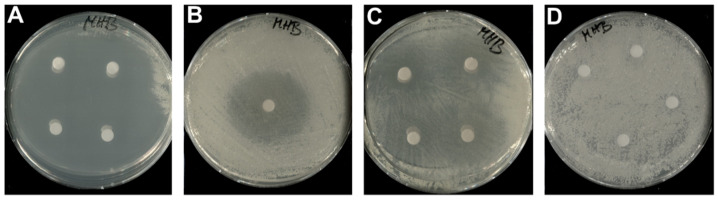
Zone of inhibition assays on agar plate containing a soft agar overlay of the test microorganism *Micrococcus luteus* DSM 1790. (**A**) White paper disks contain the well-known antibacterial drug fusidic acid as positive control. (**B**) The white paper disk contains **1**. (**C**) The white paper disks contain **2**. (**D**) White paper disks contain MeOH as negative control.

Compound **3** was reported to be weakly active against a few Gram-positive bacteria, whereas Gram-negative bacteria, yeast and fungi are resistant against **3** [15]. Compound **2** showed antibacterial activity against *Salmonella typhumirium* [16]. The myxochelin congeners A and B are known to be active against different cancer and tumor cell lines [24,28,40]. The antileukemic activity of the myxochelins can be explained through the inhibition of human 5-lipoxygenase. This well-known drug target [43], which catalyzes the conversion of arachidonic acid to leukotrienes is involved in important inflammatory processes [42,44,45]. Compound **1**–**3** showed no antibacterial activity against the Gram-negative test strains (Table 1), but **1** featured modest antibacterial activity against *Micrococcus luteus* (Figure 4) and moderate cytotoxic activity against the tested cancer cell lines being in line with **2** (Table 2). In conclusion, the succinylation of **2** seems to affect its biological activity according to the observed performance in the conducted bioactivity assays.

### 3.3. Biosynthesis of ***1**–**3***

**Figure 5:**
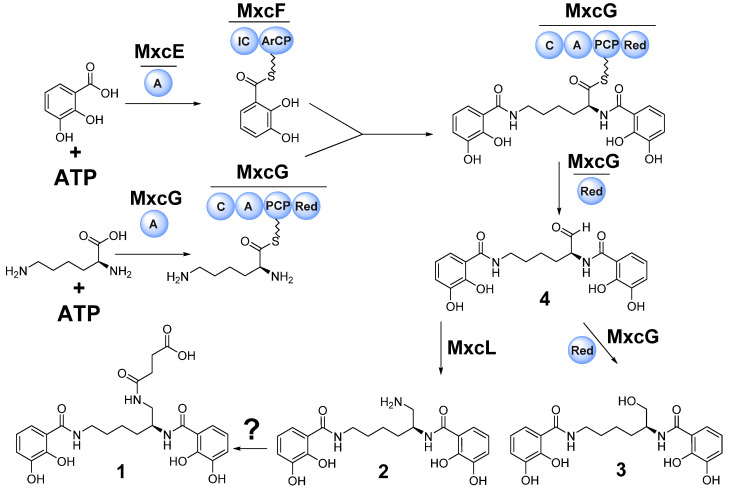
Proposed biosynthetic pathway leading to the formation of **1** alongside the previously identified congeners **2** and **3**, via the biosynthetic aldehyde intermediate **4**. Scheme adapted from Li et al. [19]. Sphere: Biosynthetic domain, PCP: Peptidyl carrier protein; A: Adenylation domain, IC: isochorismate synthase, ArCP: Aryl carrier protein, C: Condensation domain, Red: Reduction domain.

Genomic investigation of the myxobacterial strain MSr12020 led to the identification of a particular myxochelin-like biosynthetic gene cluster (BGC) that could be responsible for the biosynthesis of the new congener **1** and for the previously discovered compounds **2** and **3**. Although the architecture (localization of conserved myxochelin biosynthesis genes and surrounding accessory genes) of the identified genetic locus from MSr12020 deviates from previously investigated myxochelin BGCs (Supplementary Materials, Figure S13), the overall similarity regarding gene sequence identity, suggests a similar biosynthetic pathway leading to **1** and the known congeners **2** and **3** (Figure 5).

The myxochelin biosynthesis was elucidated by in vitro reconstitution of the complete biosynthetic pathway using the recombinantly produced core NRPS biosynthetic proteins MxcE–G as well as MxcL in *Escherichia coli* [18,19,21]. The biosynthesis of the myxochelins is initiated by ATP-dependent activation of 2,3-dihydroxybenzoic acid (2,3-DHBA) via the standalone adenylation domain MxcE. 2,3-DHBA is then transferred to the carrier protein of the bimodule MxcF, which contains an aryl-carrier protein (ArCP) domain and an isochorismate synthase (IC). Subsequently MxcF transfers two 2,3-DHBA units to MxcG for condensation with both, the α- and ε-side-chain amino groups of the activated lysine. The PCP-bound thioester intermediate is reduced and thereby released from the enzyme complex to yield aldehyde **4** which can undergo NAD(P)-H dependent catalyzed reduction to generate the corresponding alcohol **3** or reductive transamination by MxcL to produce **2** (Figure 5).

Based on the elucidated chemical structure of **1**—being a succinylated derivative of **2**—the presence of a genetically conserved myxochelin BGC and the prolific production of **2** and **3**, it is reasonable to propose that **2** is the biosynthetic precursor of **1**. Therefore, it seems to be plausible that either a single enzyme is catalyzing the succinylation of **2** to yield **1** or a non-enzymatic reaction of **2** with the reactive intracellular metabolite succinyl-CoA generates **1**.

**Figure 6:**
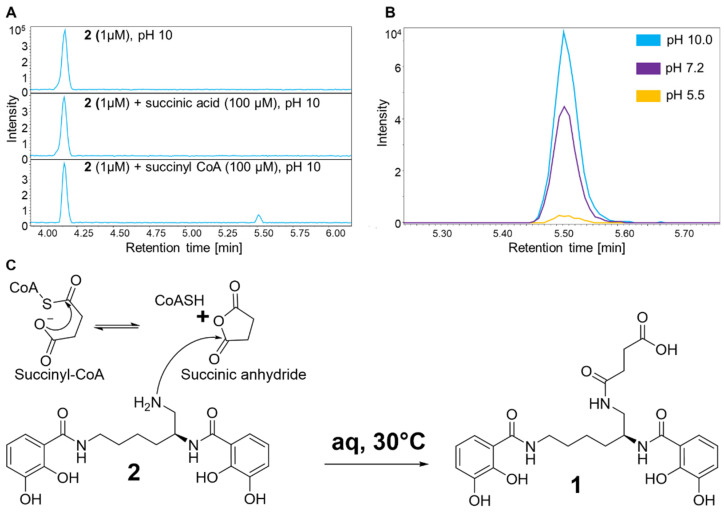
(**A**) HPLC–MS BPCs of in vitro reactions with **2.** Only in the presence of succinyl-CoA (100-fold molar excess), the conversion of **2** to **1** was observed. (**B**) Production of **1** in reaction solution (1 µM **2** and 100 µM succinyl CoA) with different pH values observed as an HPLC–MS EIC at 504.1983 ± 0.05 Da [M+H]^+^. (**C**) Proposed mechanism for the non-enzymatic succinylation of **2** during the biosynthesis of **1**. The proposed reactive cyclic succinic anhydride intermediate might react in a dose-dependent manner [49].

Since careful genomic in silico analysis of the identified myxochelin BGC in the myxobacterial strain MSr12020 did not result in a potential co-localized gene candidate, which was expected to encode a transferase responsible for the catalytic conversion of **2** to **1** (Supplementary Materials, Figure S13, Table S5), we performed different in vitro reactions by incubating **2** or **3** with succinyl-CoA or succinate under physiological conditions in order to probe a possible non-enzymatic formation of **1**. As expected, incubation of **2** or **3** with succinate did not yield any observable reaction product, whereas incubation of **2** with succinyl-CoA in fact revealed minor production of **1** (Figure 6A). This non-enzymatic reaction was clearly pH dependent, since in vitro reactions performed at pH 5.5 did only show minute amounts of **1**, whereas the succinylation rate was highest at pH 10 (Figure 6B). Similar observations considering the pH dependency of non-enzymatic succinylation reactions have been reported previously [46,47]. Compound **3** was not succinylated under the tested conditions, which resembles the finding that we could not observe any myxochelin-A-succinyl derivative in the secondary metabolome of MSr12020. The higher nucleophilicity of the primary amine of **2** compared to the primary alcohol of **3** [48] might provide a reasonable explanation for this observation. Therefore, a possible mechanism for the succinylation of **2** might comprise the nucleophilic attack of a primary amino group on the carbonyl carbon of the succinyl group in succinyl-CoA. Accordingly, an alkaline pH accelerates the formation of a highly reactive cyclic succinic anhydride intermediate, which can react with the primary amine of **2** [49] (Figure 6C).

Due to the fact that myxochelin production is prevalent and conserved within the order of *Myxococcales*, we performed an extended survey investigating the occurrence of **1**–**3** across myxobacterial taxa, using a previously established collection of high-resolution HPLC-MS datasets from approx. 2600 myxobacterial strains [39]. The chosen parameters to evaluate those MS datasets considering the exact mass (exact mass deviation below 5 ppm), isotope pattern, and retention time matching (retention time deviation below 0.3 min) was adapted from a previous study investigating the presence of myxochelin congeners featuring nicotinic acid moieties [17]. This metabolomic survey revealed that **1** is a commonly observed byproduct of myxochelin B biosynthesis (Figure S14). The observed production titer of **1** displays a great bandwidth from being produced in trace amounts up to an equally produced congener such as **3** (Figure S15). Similarly to the secondary metabolome of MSr12020, we could not observe any molecular ion, which might account for a myxochelin-A-succinyl derivative in these myxobacterial HPLC-MS datasets. It is worth mentioning that the production of iron-chelating myxochelins in myxobacteria is strongly depending on the availability of iron in the fermentation medium; consequently fermentation media supplemented with ferric ethylenediaminetetraacetic acid (EDTA) is abolishing myxochelin production [17].

Taken together, these results do not entirely exclude that a tailoring enzyme is catalyzing the formation of **1** (in particular in those observed myxobacterial high producers), however such non-enzymatic succinylations of natural products seem not to be unprecedented as exemplified by the discovery of succinylated derivatives of the subclass IId bacteriocin BacSp222 [47]. Overall, our results indicate that non-enzymatic conversion of **2** seems to be a reasonable route leading to the formation of **1**.

## 4. Discussion and Conclusions

**Figure 7:**
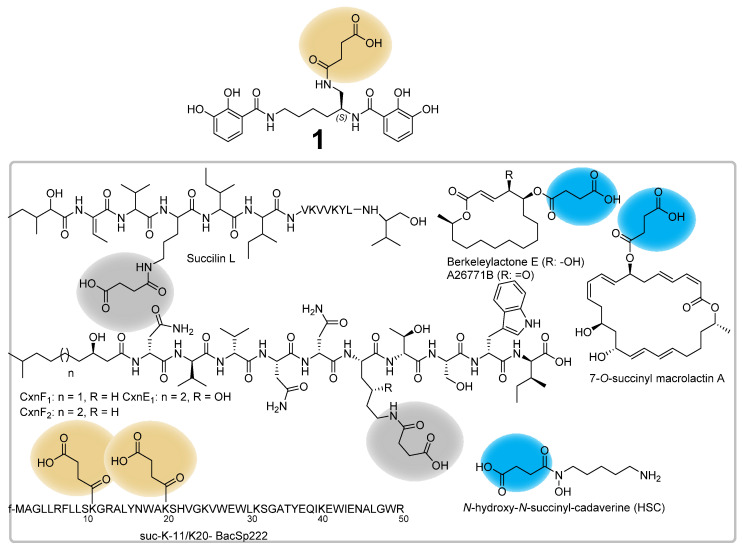
Selected examples of microbial natural products featuring a succinyl substructure. While the succinylation of berkeleylactone E, A26771B, 7-O-succinyl macrolactin A and HSC are catalyzed by acyl-transferase-like proteins (succinyl moiety colored in blue), the succinylation of **1** and suc-K11/suc-K20-BacSp222 occurs non-enzymatically (succinyl moiety colored in orange). The succinylation of the succilins and cerexins remains elusive (succinyl moiety colored in grey).

Succinylations as post-translational modification of proteins originating from bacteria, yeasts or animals were described in previous studies [50,51], whereas the modification in microbial secondary metabolite biosynthesis is a rather uncommon tailoring reaction. Examples of microbial natural products featuring succinyl substructures include the subtilin-like lantibiotic S-entianin [52], the lipopeptides cerexins [53] and succilins [54], the tetrahydroisoquinolines perquinolines A–C [55], the nonpeptide hydroxamate siderophores desferrioxamine [8], the fungal macrolides A26771B and berkeleylactone [56], the 24-membered macrolide 7-*O*-succinyl macrolactin A [57] and succinylated derivatives of the subclass IId bacteriocin BacSp222 (suc-K20-BacSp222 and suc-K11/suc-K20-BacSp222) [47] (Figure 7). The perquinolines A–C biosynthesis highlights a chemically interesting natural product assembly in which the biosynthesis is initiated by the condensation of succinyl-CoA and l-phenylalanine catalyzed by the amino-7-oxononanoate synthase-like enzyme PqrA [55]. In the course of the biosynthesis of the fungal macrolide antibiotic A26771B, the acyltransferase BerkE catalyzes the succinylation of the biosynthetic intermediate berklactone C to the succinylated intermediate berkeleylactone E [56]. The structural decoration of the 7-OH group of macrolactin A to its succinylated derivative *O*-succinyl macrolactin A is catalyzed by the a β-lactamase homolog BmmI, which could specifically attach C3–C5 alkyl acid thioesters and also exhibit substrate promiscuity toward acyl acceptors with different scaffolds [57].

Paralleling those aforementioned biosynthetic examples, the biosynthetic formation of the hydroxamate siderophores desferrioxamines involves the acyl-CoA-dependent acyl transferase DesC, which catalyzes the acylation of *N*-hydroxypentane-1,5-diamine (*N*-hydroxycadaverine) with succinyl- and acetyl-CoA to form *N*-hydroxy-*N*-acetyl-cadaverine (HAC) and *N*-hydroxy-*N*-succinyl-cadaverine (HSC) [58]. While for those natural product classes the catalysis of succinylation was identified by acyl-transferase-like proteins, the underlying succinylation mechanism of other natural product classes such as the subtilin-like lantibiotic S-entianin [52], the lipopeptides cerexins [53] and succilins [54] elusive at present.

In summary, this study describes the discovery, isolation, full structure elucidation and a possible biosynthetic pathway of **1** which shows in contrast to previous naturally and semi-synthetically produced myxochelins a unique succinylation decoration on the primary amino group. Although the biological and iron-chelation function of the succinyl group of **1** remains elusive, we observed that the succinylation of **2** leads to a significant reduction in antibacterial activity. In silico genome analysis of the myxobacterium MSr12020 revealed the genetic origin of **1**–**3** and in vitro experiments display the possibility that **1** might be biosynthesized by non-enzymatic succinylation of **2** with the highly reactive intracellular metabolite succinyl-CoA. Further confirmation of this finding would hint towards interesting biological implications regarding the purpose of succinylation during the biosynthesis of natural products and as posttranslational modification. It seems that both enzymatic and non-enzymatic succinylations play a central role in living (micro)organisms [59], and future studies might provide a better understanding concerning the imposing consequences of these chemical modifications. Thus, the discovery and proposed biosynthesis of **1** from the myxobacterium MSr12020 provides an intriguing puzzle piece of natural product succinylation.

## Figures and Tables

**Table 1:** Antimicrobial activity of myxochelin-B-succinate (**1**), myxochelin B (**2**), myxochelin A (**3**) and different well-known antimicrobial drugs as control against common microbial pathogens. NT: not tested.

	Zone of Inhibition in mm						
Microorganism	1	2	3	Kanamycin	Fusidic Acid	Cycloheximide	MeOH/DMSO
*Escherichia coli* HS 996	6	6	6	30	NT	NT	6
*E. coli* BW251123	6	6	6	32	NT	NT	6
*Micrococcus luteus* DSM 1790	40	40	6	NT	44	NT	6
*Bacillus subtilis* DSM 10	6	10	6	NT	27	NT	6
*Mucor hiemalis* DSM 2656	6	6	6	NT	NT	15	6
*Pichia anomala* DSM 6766	6	6	6	NT	NT	52	6

**Table 2:** Cytotoxic activity of myxochelin-B-succinate (**1**), myxochelin B (**2**), myxochelin A (**3**) and doxorubicin (well-known cytotoxic drug) as control.

	IC_50_ Values of 1–3 in μg/mL			
Cancer Cell Line	1	2	3	Doxorubicin
HCT-116	23.3	23.2	24.3	0.1
KB-3-1	22.7	21.2	41.7	0.6
U-2 OS	31.5	29.1	34.6	0.2

## Data Availability

All data presented in this study are available from the corresponding author on reasonable request.

## Supplementary Materials

The following supporting information can be downloaded at: https://www.mdpi.com/article/10.3390/microorganisms10101959/s1, Figure S1: LC MS UV-VIS spectrum of myxochelin B-succinate (**1**); Figure S2: MS^2^ fragmentation of myxochelin B-succinate (**1**), Figure S3: MS^2^ fragmentation of myxochelin-B succinate (**1**); Figure S4: MS^2^ spectrum of in vitro produced **1** (**A**) and authentic **1** (**B**); Figure S5: LC MS UV-VIS spectrum of myxochelin B (**2**); Figure S6: MS^2^ fragmentation of myxochelin B (**2**); Figure S7: MS^2^ spectrum of myxochelin B (**2**); Figure S8: LC MS UV-VIS spectrum of myxochelin A (**3**); Figure S9: MS^2^ fragmentation of myxochelin A (**3**); Figure S10: MS^2^ fragmentation of myxochelin A (**3**); Figure S11: MS^2^ fragmentation scheme of **1** and proposed structure of identified key ions; Figure S12: CD spectrum of **1** at a concentration of 0.5 mg/mL in MeOH in the area 190–400 nm; Figure S13: Comparison of *mxc* BGCs that have been characterized to produce different myxochelins in different myxobacterial strains and *Pseudomonasalteromonas piscicida*; Figure S14: Alternative producers of **1** from our in-house metabolome database sorted by suborder; Figure S15: High performance liquid chromatography–mass spectrometry extracted ion chromatograms (HPLC–MS EIC) of **1** orange ([M+H]^+^ 504.1976 m/z, (orange), **2** ([M+H]^+^ 404.1819 m/z, blue), and **3** ([M+H]^+^ 405.1658 m/z, black) from the myxobacterial crude extracts of MCy9555, MCy10589, MCy9523 and MCy9472; Figure S16: ^1^H NMR of compound **1**; Figure S17: ^1^H NMR spectrum of compound **1** (expanded part 1); Figure S18: ^1^H NMR spectrum of compound **1** (expanded part 2); Figure S19: ^13^C NMR spectrum of compound **1**; Figure S20: ^1^H-^1^H DQF-COSY spectrum of compound **1**; Figure S21: HSQC spectrum of compound **1**; Figure S22: HSQC spectrum of compound **1** (expanded); Figure S23: HSQC spectrum of compound **1** (expanded); Figure S24: ^1^H-^13^C HMBC spectrum of compound **1**; Figure S25: ^1^H-^13^C HMBC spectrum of compound **1** (expanded part 1); Figure S26: ^1^H-^13^C HMBC spectrum of compound **1** (expanded part 2); Figure S27: ^1^H-^13^C HMBC spectrum of compound **1** (expanded part 3); Figure S28: ^1^H NMR spectrum of compound **2**.; Figure S29: ^1^H NMR spectrum of compound **2** (expanded part 1); Figure S30: ^1^H NMR spectrum of compound **2** (expanded part 2); Figure S31: ^13^C NMR spectrum of compound **2** in MeOD; Figure S32: ^13^C NMR spectrum of compound **2** (expanded part 1); Figure S33: ^13^C NMR spectrum of compound **2** (expanded part 2); Figure S34: ^1^H-^1^H DQF-COSY spectrum of compound **2**; Figure S35: ^1^H-^1^H DQF-COSY spectrum of compound **2**; Figure S36: ^1^H-^1^H DQF-COSY spectrum of compound **2** (expanded); Figure S37: HSQC spectrum of compound **2**; Figure S38: HSQC spectrum of compound **2** (expanded part 1); Figure S39: HSQC spectrum of compound **2** (expanded part 2); Figure S40: ^1^H-^13^C HMBC spectrum of compound **2**; Figure S41: ^1^H-^13^C HMBC spectrum of compound **2** (expanded part 1); Figure S42: ^1^H-^13^C HMBC spectrum of compound **2** (expanded part 2); Figure S43: ^1^H-^13^C HMBC spectrum of compound **2** (expanded part 3); Figure S44: ^1^H-^13^C HMBC spectrum of compound **2** (expanded part 4); Figure S45: ^1^H NMR spectrum of compound **3**; Figure S46: ^1^H NMR spectrum of compound **3** (expanded part 1); Figure S47: ^1^H NMR spectrum of compound **3** (expanded part 2); Figure S48: ^13^C NMR spectrum of compound **3**; Figure S49: ^13^C NMR spectrum of compound **3** (expanded part 1); Figure S50: ^13^C NMR spectrum of compound **3** (expanded part 2); Figure S51: ^1^H-^1^H DQF-COSY spectrum of compound **3**; Figure S52: ^1^H-^1^H DQF-COSY spectrum of compound **3** (expanded part 1); Figure S53: ^1^H-^1^H DQF-COSY spectrum of compound **3** (expanded part 2); Figure S54: HSQC spectrum of compound **3**; Figure S55: HSQC spectrum of compound **3** (expanded part 1); Figure S56: ^1^H-^1^H DQF-COSY spectrum of compound **3** (expanded part 2); Figure S57: ^1^H-^13^C HMBC spectrum of compound **3**; Figure S58: ^1^H-^13^C HMBC spectrum of compound **3** (expanded part 1); Figure S59: ^1^H-^13^C HMBC spectrum of compound **3** (expanded part 2); Figure S60: ^1^H-^13^C HMBC spectrum of compound **3** (expanded part 3); Figure S61: ^1^H-^13^C HMBC spectrum of compound **3** (expanded part 4); Table S1: Spectroscopic values of myxochelin B-succinate (**1**) acquired in CD_3_OD at 500 MHz; Table S2: Spectroscopic values of myxochelin B (**2**) acquired in CD_3_OD at 500 MHz; Table S3: Spectroscopic values of myxochelin A (**3**) acquired in CD_3_OD at 500 MHz; Table S4: Predicted functions of the encoded proteins by the Myxochelin BGC from MSr12020 (44350 bp).
