# Supplementary material for: Discovery, Biosynthesis and Biological Activity of a Succinylated Myxochelin from the Myxobacterial Strain MSr12020
Source: Microorganisms. 2022 Sep 30;10(10):1959. doi: 10.3390/microorganisms10101959 (PMC9611931; doi:10.3390/microorganisms10101959)
Supplement: Supplementary file 1 [file microorganisms-10-01959-s001.zip › microorganisms-1934079-supplementary.pdf]

Supplementary Information

Discovery, Biosynthesis and Biological Activity of a Succinylated Myxochelin from the Myxobacterial strain MSr12020

Dorothy A. Okoth [1,2,3,4,5+], Joachim J. Hug [1,2,3,4+], Ronald Garcia [1,2,3,4] and Rolf Müller [1,2,3,4*]

[1] Helmholtz-Institute for Pharmaceutical Research Saarland (HIPS), Helmholtz Centre for Infection Research (HZI), Department of Microbial Natural Products, Saarland University, Campus E8 1, 66123 Saarbrücken, Germany

[2] Department of Pharmacy, Saarland University, 66123 Saarbrücken, Germany

[3] German Center for Infection Research (DZIF), Partner Site Hannover-Braunschweig, 38124 Braunschweig, Germany

[4] Helmholtz International Labs, Department of Microbial Natural Products, Saarland University, Campus E8 1, 66123 Saarbrücken, Germany

[5] Department of Chemistry, School of Physical and Biological Sciences, Main campus, Maseno University, Maseno P.O. Box 333-40105, Kenya

[*] Correspondence and primary affiliation: rolf.mueller@helmholtz-hips.de; Tel.: +49-681-98806-3000, Helmholtz-Institute for Pharmaceutical Research Saarland (HIPS), Helmholtz Centre for Infection Research (HZI), Department of Microbial Natural Products, Saarland University, Campus E8 1, 66123 Saarbrücken, Germany

[+] These authors contributed equally to this work.

Table of Contents

1. Analytical data 3

  1.1 Partial ESI-MS spectra and MS^2 spectra 3

  1.2 Proposed fragmentation pattern of 1 10

  1.3 CD spectrum of myxochelin-B-succinate (1) 11

2. NMR spectroscopic data 12

3. Biosynthetic *in silico* investigation 15

4. Metabolome database search of 1–3 17

5. ^{1}H and ^{13}C NMR spectra 19

6. References 66

1. Analytical data

1.1 Partial ESI-MS spectra and MS^2 spectra

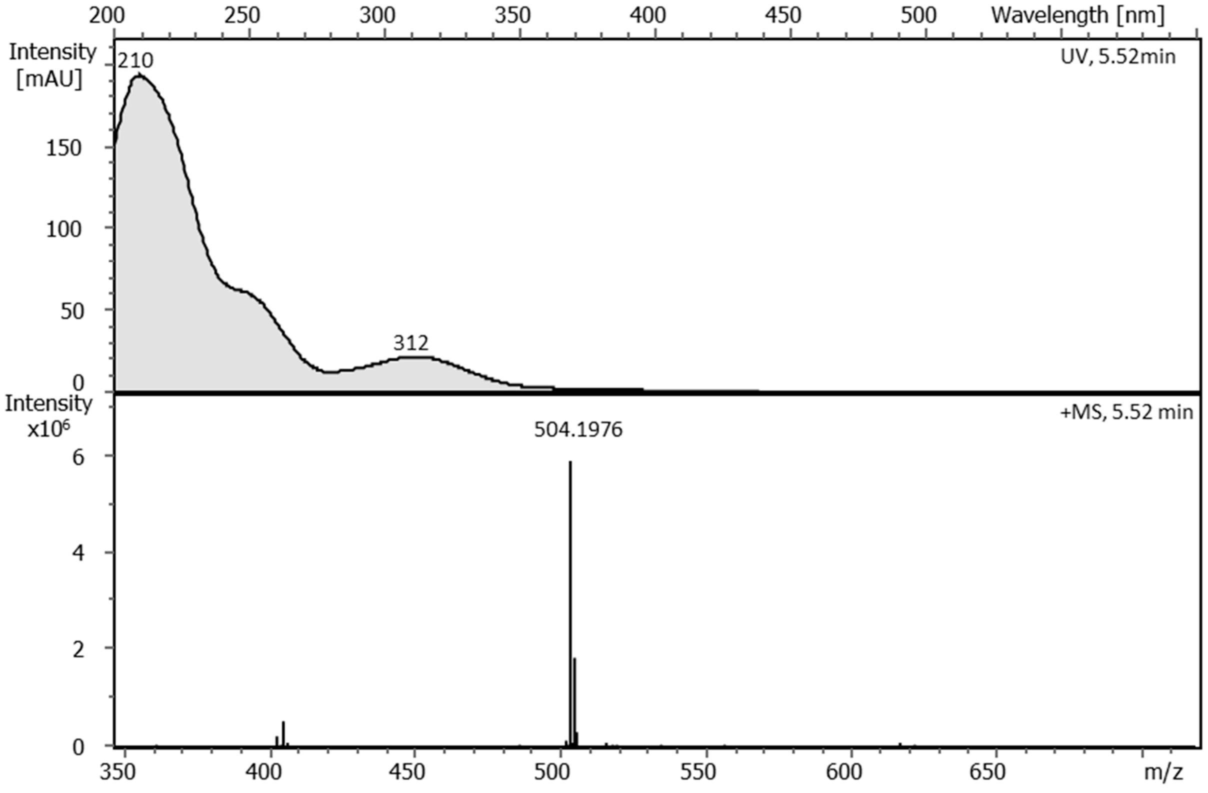

Figure S1. LC MS UV-VIS spectrum of myxochelin B-succinate (**1**).

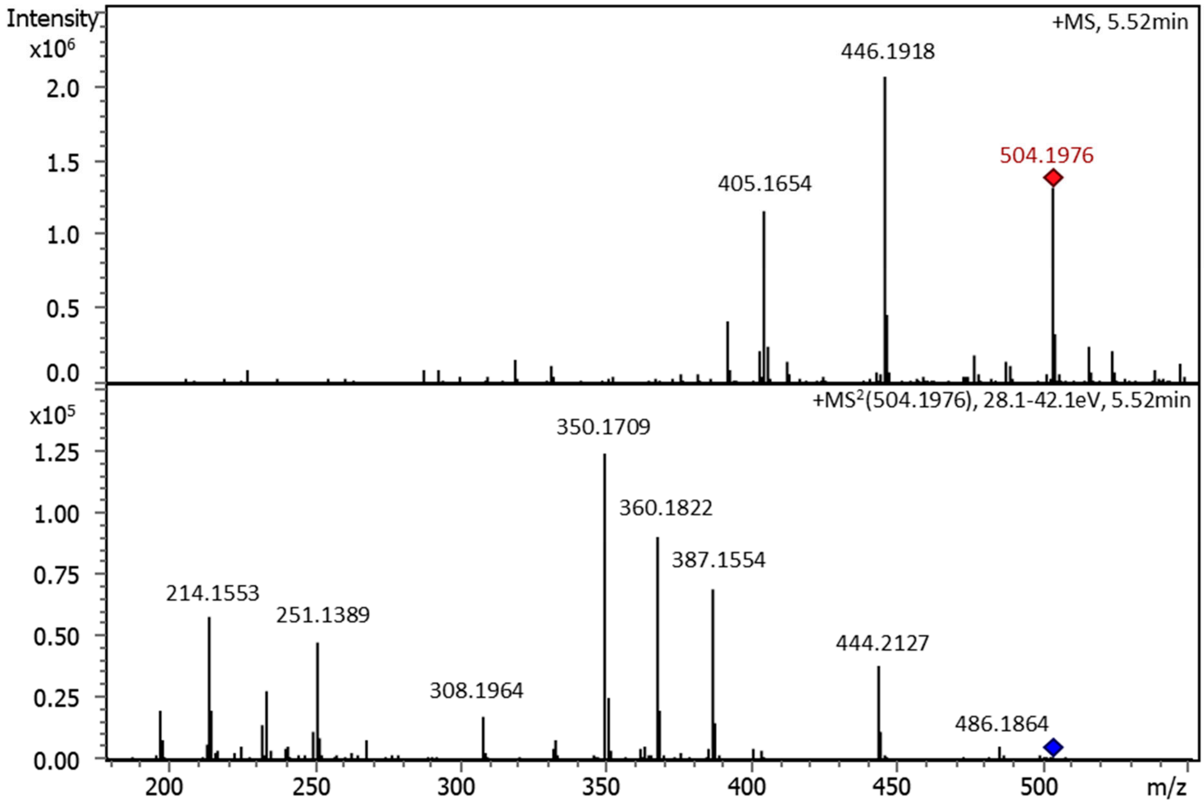

Figure S2. MS^2 fragmentation of myxochelin B-succinate (**1**).

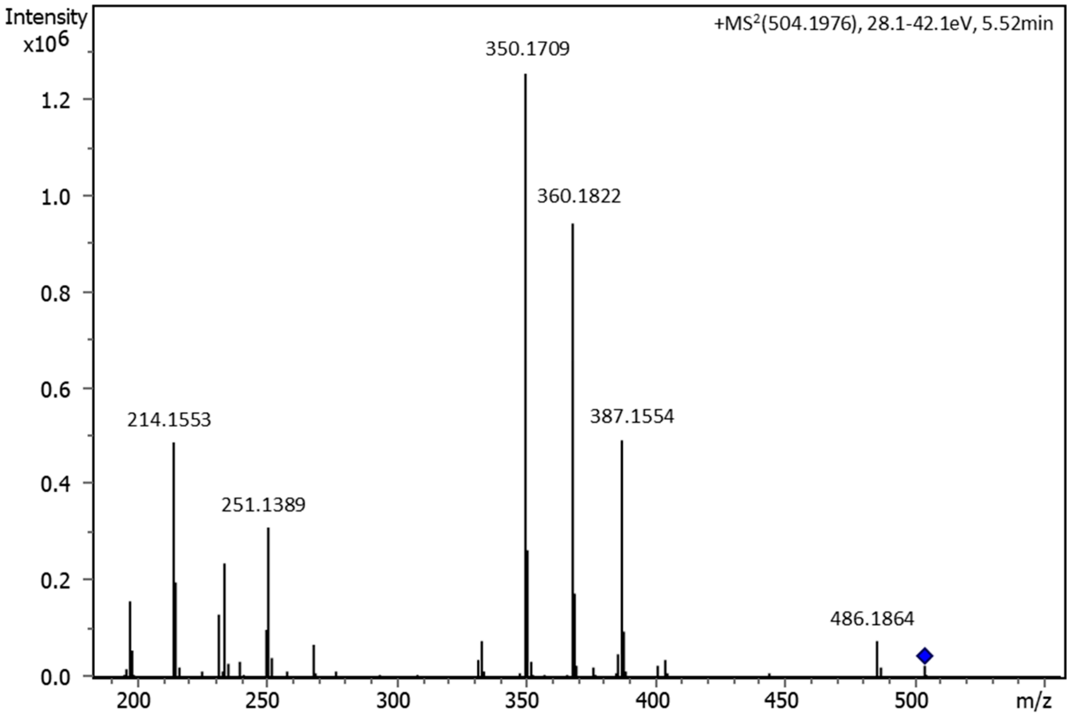

Figure S3. MS^2 fragmentation of myxochelin-B succinate (**1**).

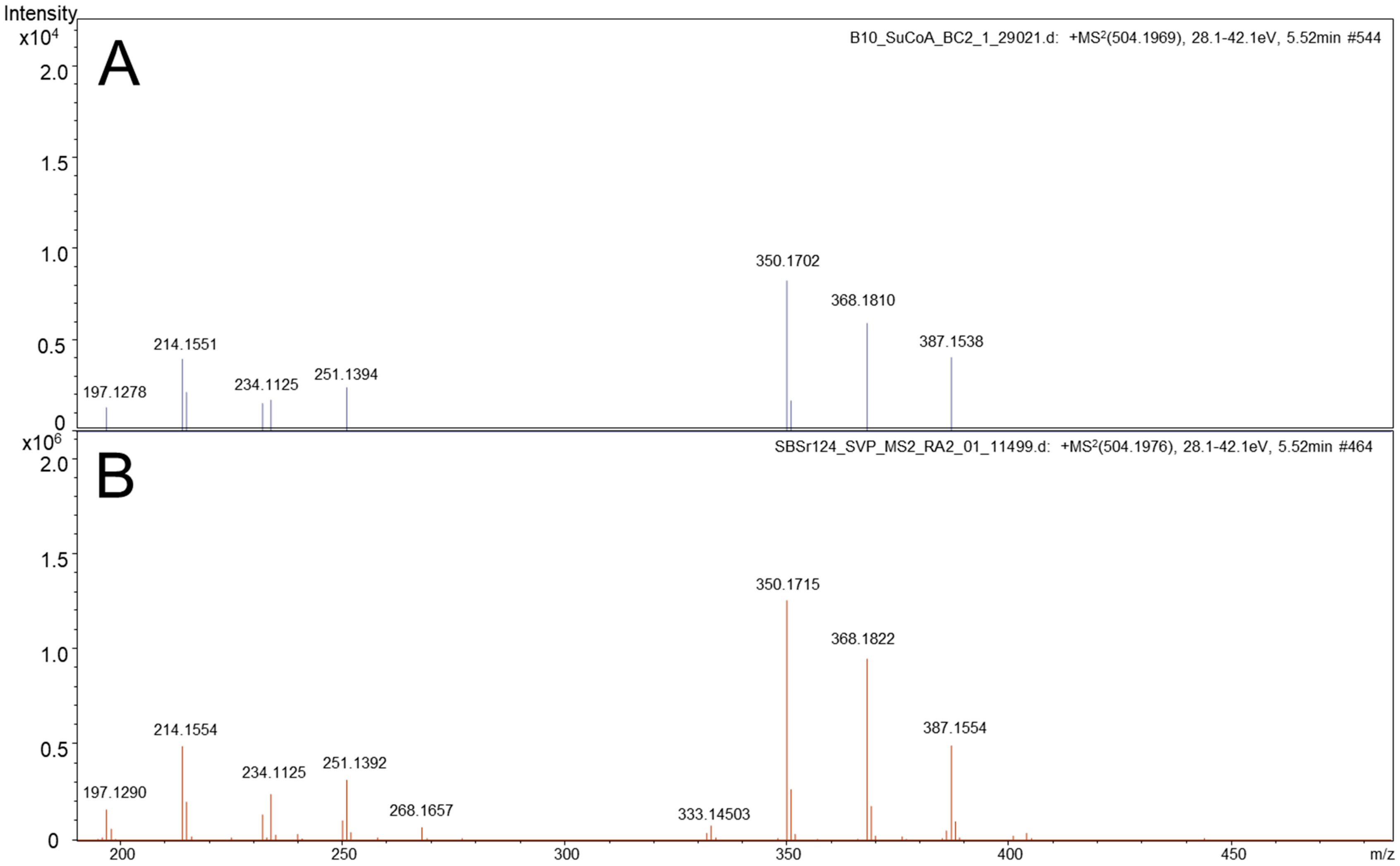

Figure S4. MS^2 spectrum of *in vitro* produced **1** (**A**) and authentic **1** (**B**). *In vitro* produced **1** ($[M+H]^+$ calcd for $C_{24}H_{30}N_3O_9$, 504.1977; found 504.1969; Δ 1.6 ppm) featured a slightly increased mass difference (Δ 1.6 ppm) than authentically produced **1** (Δ 0.2 ppm). Nevertheless, the identical MS^2 fragmentation pattern and retention time confirm that authentic and *in vitro* produced **1** are identical.

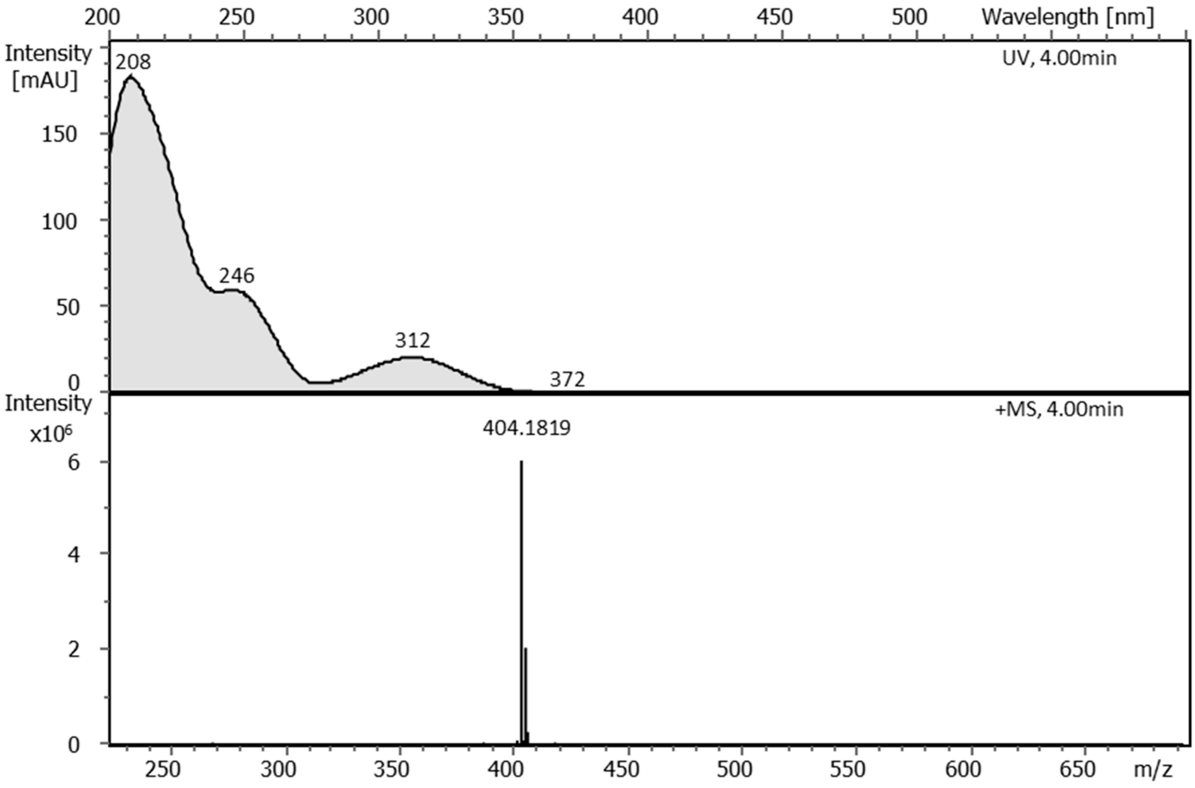

Figure S5. LC MS UV-VIS spectrum of myxochelin B (**2**).

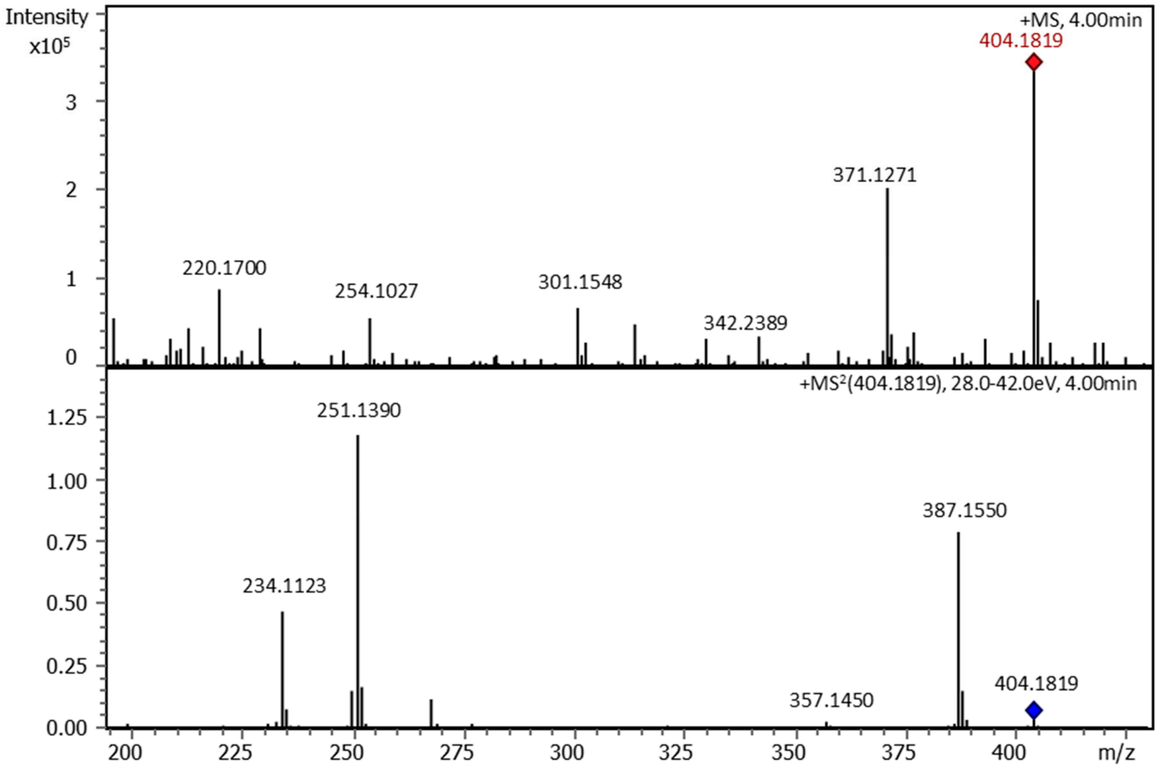

Figure S6. MS^2 fragmentation of myxochelin B (**2**).

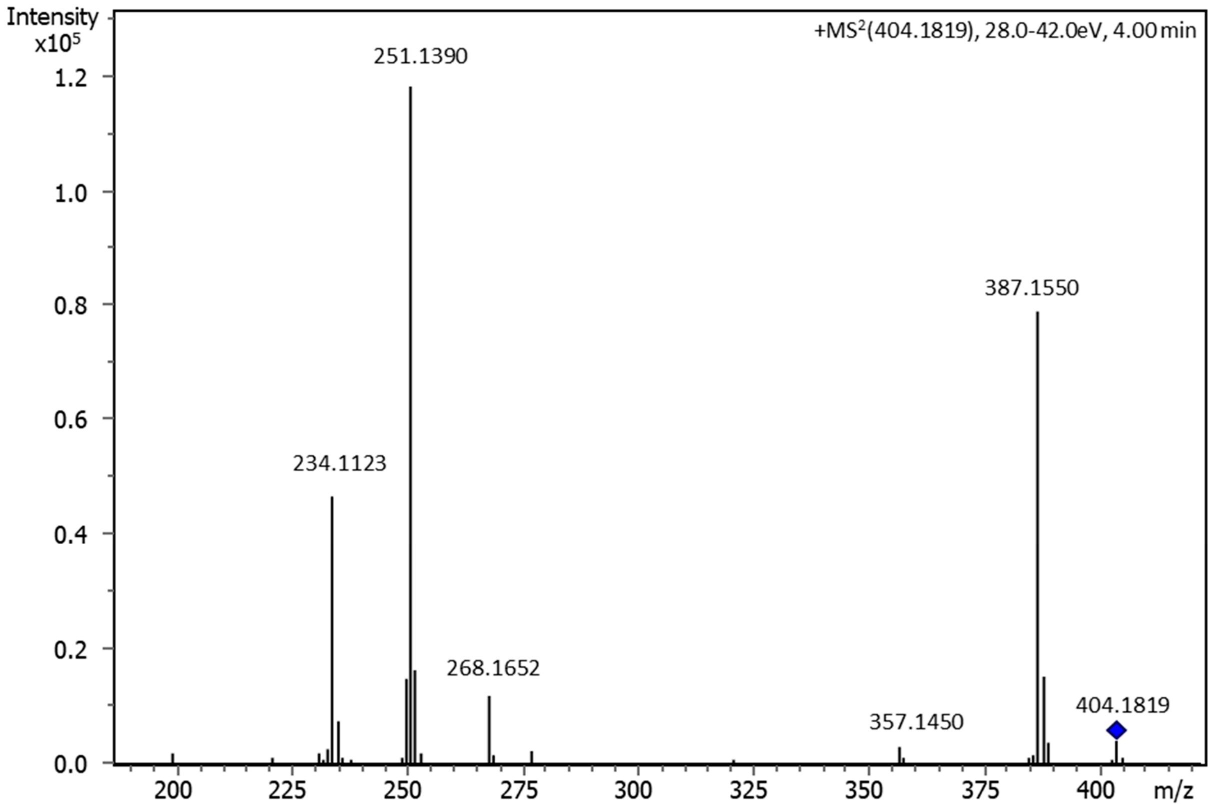

Figure S7. MS^2 spectrum of myxochelin B (**2**).

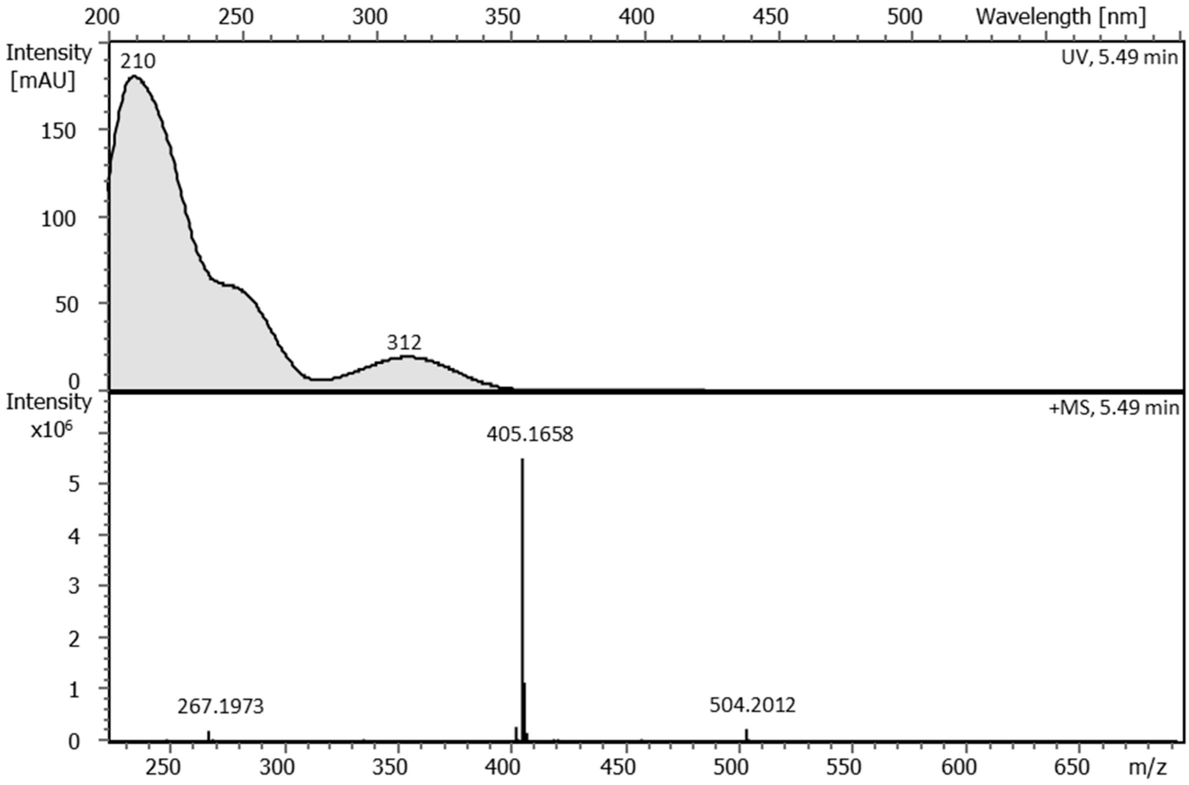

Figure S8. LC MS UV-VIS spectrum of myxochelin A (**3**).

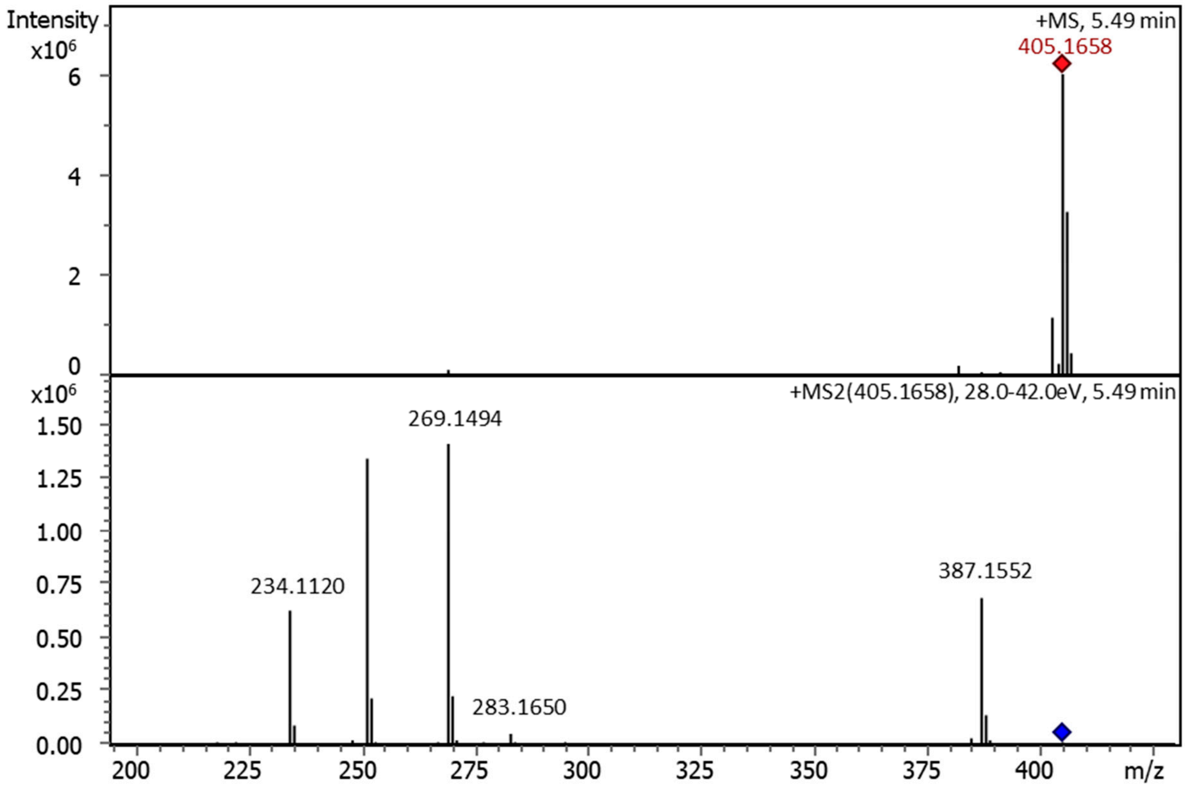

Figure S9. MS^2 fragmentation of myxochelin A (**3**).

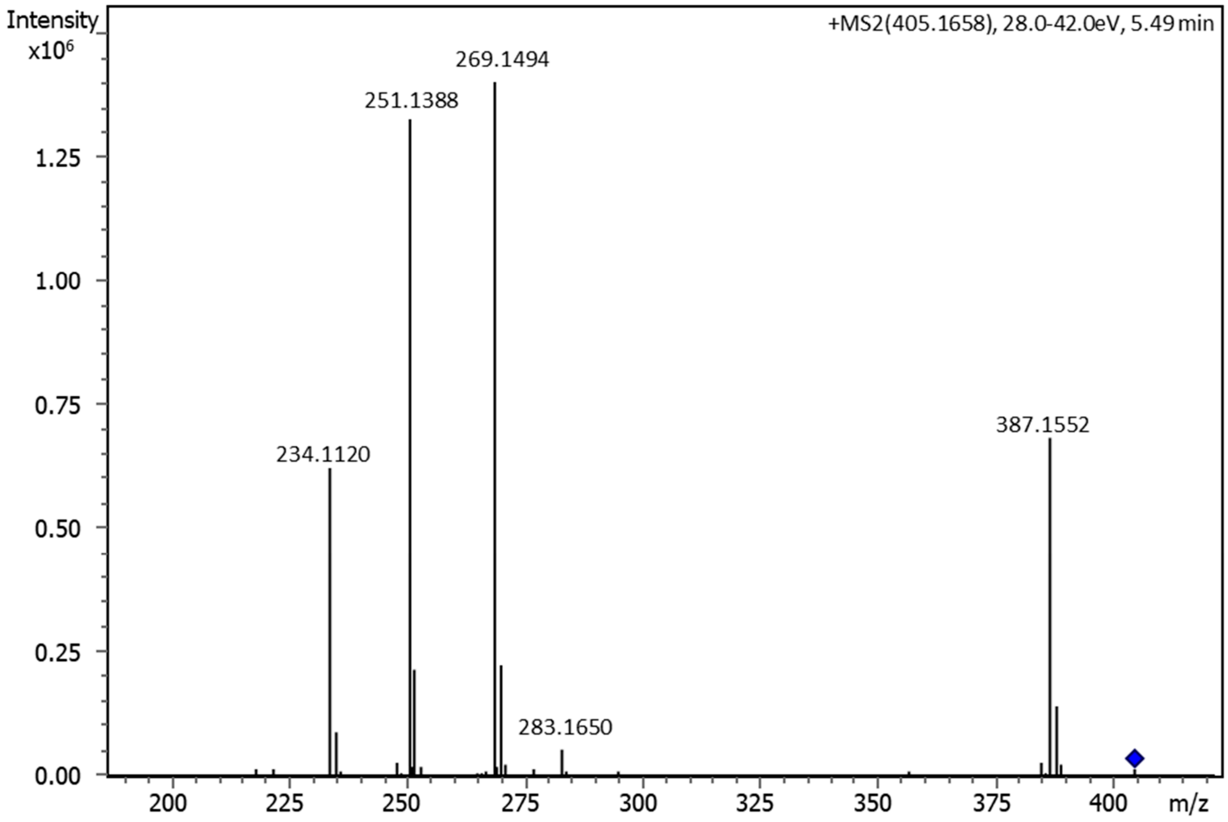

Figure S10. MS^2 fragmentation of myxochelin A (**3**).

1.2 Proposed fragmentation pattern of 1

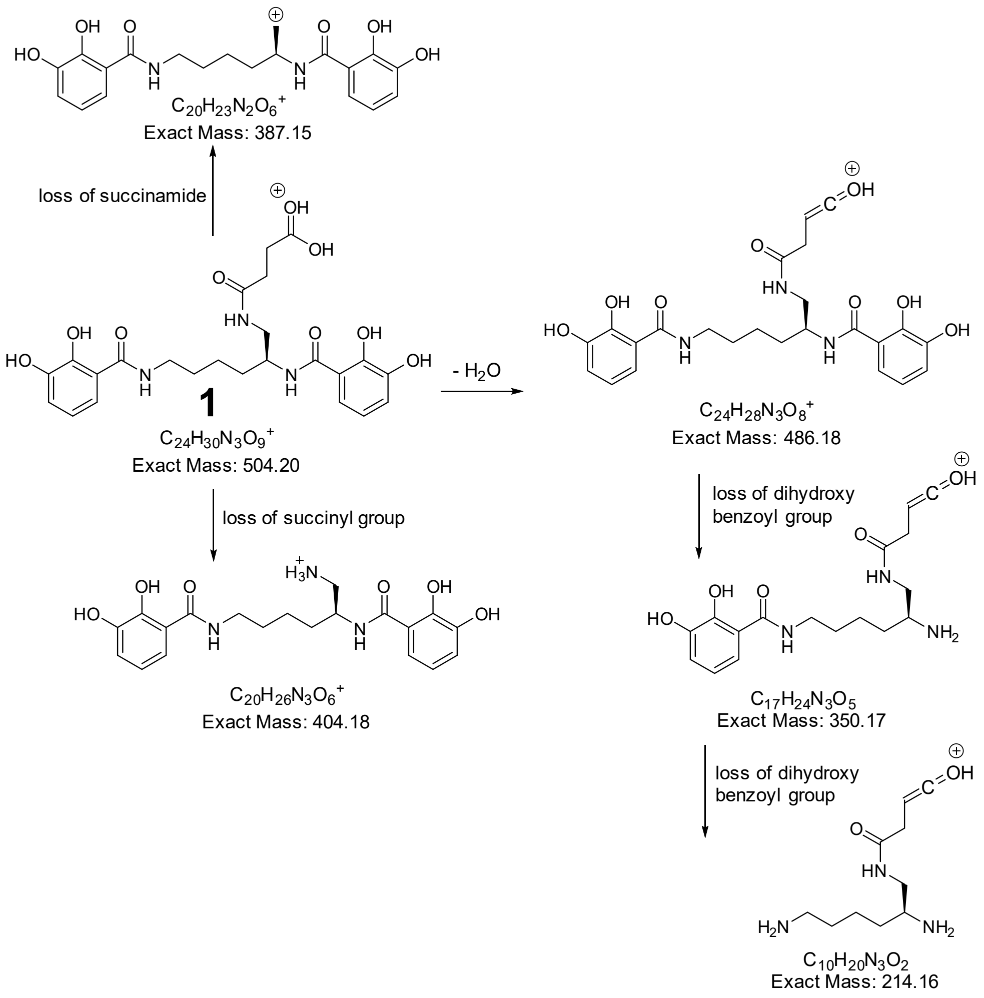

Figure S11. MS^2 fragmentation scheme of **1** and proposed structure of identified key ions.

1.3 CD spectrum of myxochelin-B-succinate (1)

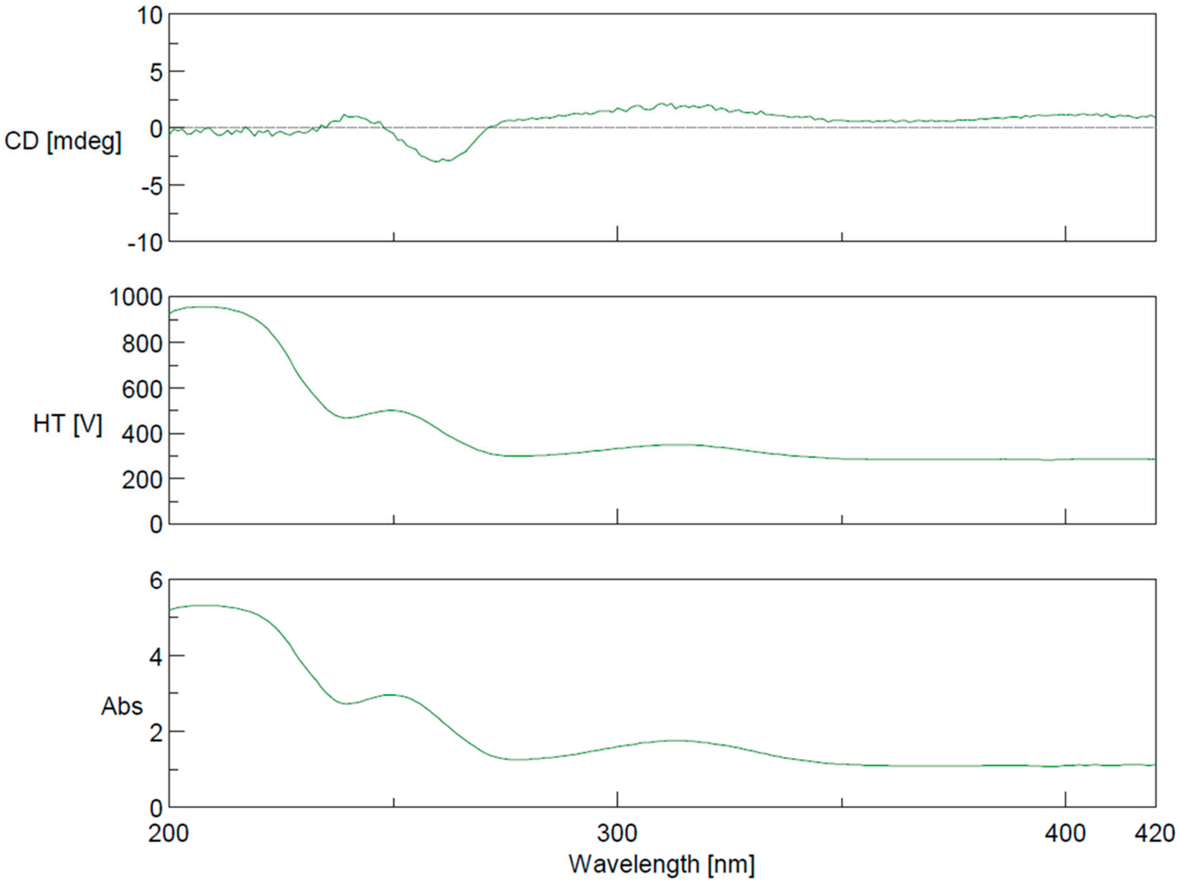

Figure S12. CD spectrum of **1** at a concentration of 0.5 mg/mL in MeOH in the area 190–400 nm.

2. NMR spectroscopic data

Table S1. Spectroscopic values of myxochelin B-succinate (**1**) acquired in CD_3OD at 500 MHz.

Myxochelin B-succinate (**1**)				
**Position**	**δ_C**	**δ_H, [m, *J* (Hz)]**	**^1H-^1H COSY**	**^1H-^{13}C HMBC**
1	44.1	3.44 1H, dd (4.85, 13.8)<br>3.29, 1H, dd (7.3, 13.8)	H-2	C-1, C-2, C-3, C-1'''
2	51.2	4.21, 1H, m	H-1, H-3	C-1, C-3, C-4, 7'
3	32.6	1.67, 2H, m	H-2, H-4	C-1, C-2, C-4, C-5
4	24.6	1.48, 2H, m	H-3, H-5	C-2,C-3,C-5, C-6
5	30.3	1.67, 2H, m	H-4, H-6	C-3, C-4, C-6
6	40.4	3.38, 2H, t (7.0)	H-5	C-4, C-5,C-7''
1'	116.9	-	-	
2'	150.4	-	-	
3'	147.5	-	-	
4'	119.7	6.92, 1H, dd (1.45, 7.9)	H-5'	C-1', C-2', C-3'C-6'
5'	119.7	6.70, 1H, dd (7.9, 8.0)	H-4', H-6'	C-1', C-2', C-3', C-6'
6'	118.7	7.18, 1H, dd (1.45, 8.0)	H-5'	C-1', C-2', C-3', C-4', C-5', C-7'
7'	171.7	-	-	
1''	116.9	-	-	
2''	150.5	-	-	
3''	147.4	-	-	
4''	119.7	6.90. 1H, dd (1.45, 8.0)	H-5''	C-1'', C-2'', C-3'',C-6''
5''	119.7	6.68, 1H, dd (7.86, 8.0)	H-4'',H-6''	C-1'', C-2'', C-3'', C-6''
6''	118.9	7.21, 1H, dd (1.45, 7.85)	H-5''	C-1'', C-2'', C-3'', C-4'', C-5'', C-7''
7''	171.8	-	-	
1'''	175.5	-	-	
2'''	31.9	2.44, 2H, m	H-3'''	C-1''', C-3''', C-4'''
3'''	30.7	2.55, 2H, t (6.85)	-	C-1''', C-2''', C-4'''
4'''	176.8	-	-	

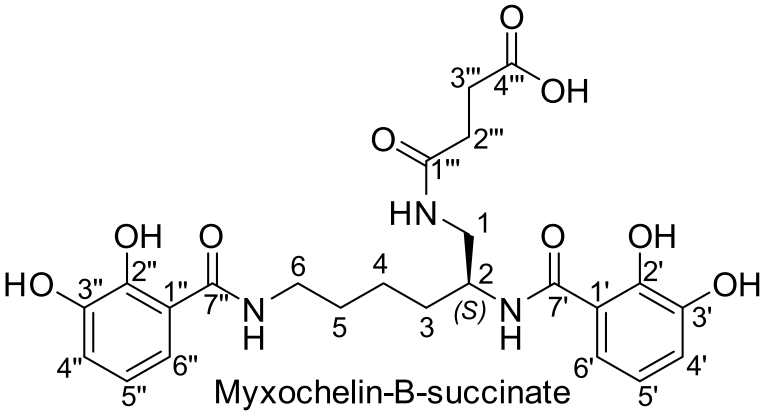

Myxochelin-B-succinate
1

Table S2. Spectroscopic values of myxochelin B (**2**) acquired in CD_3OD at 500 MHz.

Myxochelin B (**2**)				
**Position**	**δC**	**δH, [m, *J* (Hz)]**	**#δC**	**#δH, [m, *J* (Hz)]**
1	45.1	3.19, 1H, brs<br>3.04, 1H, brs	44.0	3.22, 1H, dd (3.9, 13.0)<br>3.07, 1H, ddd (9.8,3.9, 13.0)
2	49.6	4.38, 1H, brs	48.7	4.41, 2H (m)
3	32.7	1.72, 2H, m	31.3	1.70, 2H, t (m)
4	24.4	1.50, 2H, m	22.9	1.56, 2H, (m)
5	30.1	1.69, 2H, m	28.7	1.77, 2H, t (9.0)
6	40.2	3.38, 2H, t (6.05)	38.6	3.43, 2H, t (6.8)
1'	116.9	-	115.7	-
2'	150.3	-	151.6	-
3'	147.4	-	147.0	-
4'	118.8	6.92, 1H, d (7.7)	117.8	6.95, 1H, dd (1.5, 7.8)
5'	119.7	6.69 1H, t (7.7)	116.0	6.76, 1H, t (8.0)
6'	118.7	7.16 1H, d, (7.7)	114.1	7.19, 1H, dd (1.5, 8.1)
7'	171.7	-	169.3	-
1''	116.8	-	115.7	-
2''	150.4	-	153.6	-
3''	147.5	-	147.6	-
4''	120.8	6.92, 1H, d (7.7)	118.4	6.98, 1H ,dd (1.5, 8.1)
5''	119.7	6.69, 1H, d (7.7)	117.2	6.72, 1H, t, (8.0)
6''	119.2	7.20, 1H, d (7.7)	115.5	7.33, 1H ,dd (1.5, 8.1)
7''	172.7	-	169.9	-

#; NMR data acquired by Ambrosi et al. [1]; ^{13}C NMR (75 MHz DMSO-d_6), ^{1}H NMR (300 MHz DMSO-d_6),

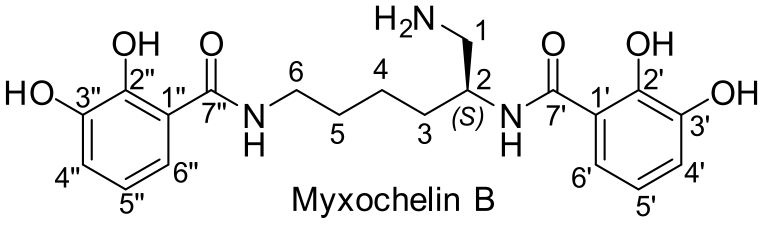

Myxochelin B
2

Table S3. Spectroscopic values of myxochelin A (**3**) acquired in CD_3OD at 500 MHz.

Myxochelin A (**3**)				
**Position**	**δC**	**δH, [m, *J* (Hz)]**	#δC	#**δH, [m, *J* (Hz)]**
1	65.2	3.62 ,2H, dd (2.5, 5.31)	63.1	3.47,1H, dd (5.9,10.8)<br>3.43,1H, dd (5.9,10.8)
2	52.8	4.16,1H, m	51.2	4.00, 2H, (m)
3	31.8	1.76, 1H, m<br>1.64, 1H, m	30.1	1.54, 2H, m
4	24.7	1.50, 2H m	23.2	1.34, 2H, (m)
5	30.4	1.70, 2H, m	28.8	1.55,2H, (m)
6	40.5	3.38, 2H, t (7.0)	48.6	3.27, 2H, (m)
1’	116.9	-	115.3	-
2’	150.4	-	149.5	-
3’	147.5	-	146.1	-
4’	119.7	6.91,1H, t (8.0)	118.7	6.91, 1H, d (7.8)
5’	119.7	6.69, 1H, q, (8.0)	117.8	6.68, 1H, t (7.8)
6’	118.7	7.16, 1H, d (8.0)	117.5	7.36, 1H, d (8.0)
7’	171.6	-	169.5	-
1’’	117.2	-	114.9	-
2’’	150.2	-	149.7	-
3’’	147.5	-	146.2	-
4’’	119.7	6.91,1H, t (8.0)	118.7	6.90, 1H, d (7.8)
5’’	119.7	6.69, 1H, q, (8.0)	117.7	6.66, 1H, t (7.8)
6’’	119.1	7.28, 1H, dd (1.15,8.0)	117.1	7.26, 1H, d (8.0)
7’’	171.5	-	169.7	-

#; NMR data acquired by Ambrosi et al. [1]; ^{13}C NMR (75 MHz DMSO-d_6), ^{1}H NMR (300 MHz DMSO-d_6),

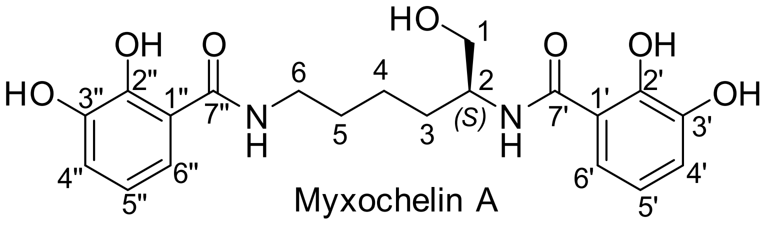

Myxochelin A
3

3. Biosynthetic *in silico* investigation

Table S4. Predicted functions of the encoded proteins by the Myxochelin BGC from MSr12020 (44350 bp).

Gene	Size (aa)	Deduced function	Closest homolog	Coverage/ Identity (%)	Sg a15 homolog[1]	So ce56 homolog[1]
orf1	283	Acetoacetate decarboxylase family protein	MCA9695171.1	86/62	---	---
orf2	345	Hypothetical protein	WP_136924859.1	100/87	---	---
orf3	200	Glutathione S-transferase *N*-terminal domain-containing protein	WP_061606175.1	100/88	---	---
orf4	370	Peptide-*N*-glycosidase	WP_136924836.1	87/89	---	---
orf5	261	Hypothetical protein	WP_043395572.1	98/38	---	---
orf6	216	Hypothetical protein	WP_169508602.1	97/91	---	---
mxcK	399	Myxochelin export MFS transporter MxcK	UQA63142.1	100/80	75/64	54/36
$aroA_{A5}$	486	3-deoxy-7-phosphoheptulonate synthase	WP_153824289.1	99/86	61/44	---
mxcD	415	Isochorismate synthase DhbC	WP_153824288.1	96/80	65/52	73/58
mxcE	554	(2,3-dihydroxybenzoyl)adenylate synthase	WP_136928082.1	97/84	78/66	84/76
mxcF	307	Isochorismatase family protein	WP_136919899.1	100/84	71/53	72/61
mxcC	258	2,3-dihydro-2,3-dihydroxybenzoate dehydrogenase	WP_153824285.1	100/88	77/66	85/77
mxcO	324	PepSY domain-containing protein	WP_153824284.1	100/83	---	83/69
mxcJ	407	Hypothetical protein	WP_153824283.1	100/83	---	76/66
mxcH	847	TonB-dependent siderophore myxochelin receptor MxcH	WP_136928077.1	100/85	66/52	72/61
mxcL	439	Aminotransferase class III-fold pyridoxal phosphate-dependent enzyme	WP_136928076.1	100/88	77/62	87/79
mxcG	1449	Myxochelin NRPS MxcG	WP_136928075.1	100/81	74/62	77/68
orf8	259	SDR family oxidoreductase	WP_093287969.1	100/67	---	---
orf9	296	LysR family transcriptional regulator	WP_114828546.1	98/75	---	---
orf10	236	Siderophore-interacting protein	WP_130432947.1	91/70	---	---
orf11	156	DNA-binding MarR family transcriptional regulator	RZT94937.1	85/63	---	---
orf12	1109	Hypothetical protein	KYF73658.1	86/53	---	---
orf13	465	Ig-like domain-containing protein	WP_169508603.1	100/83	---	---
orf14	319	Alpha/beta hydrolase	WP_136929219.1	99/83	---	---
orf15	225	Coq4 family protein	WP_136924851.1	100/82	---	---
orf16	200	TetR/AcrR family transcriptional regulator	WP_136924852.1	96/88	---	---
orf17	358	Hypothetical protein	WP_136923881.1	82/84	---	---
orf18	377	VWA domain-containing protein	UQA60575.1	100/76	---	---
orf19	766	Immune inhibitor A	WP_136923883.1	100/74	---	---
orf20	409	Cytochrome P450	WP_136924847.1	100/89	---	---
orf21	399	Chitinase	WP_136924846.1	100/83	---	---
orf22	377	Gfo/Idh/MocA family oxidoreductase	WP_169508601.1	99/85	---	---

[1] Pairwise Positive (BLSM62)/Pairwise Identity

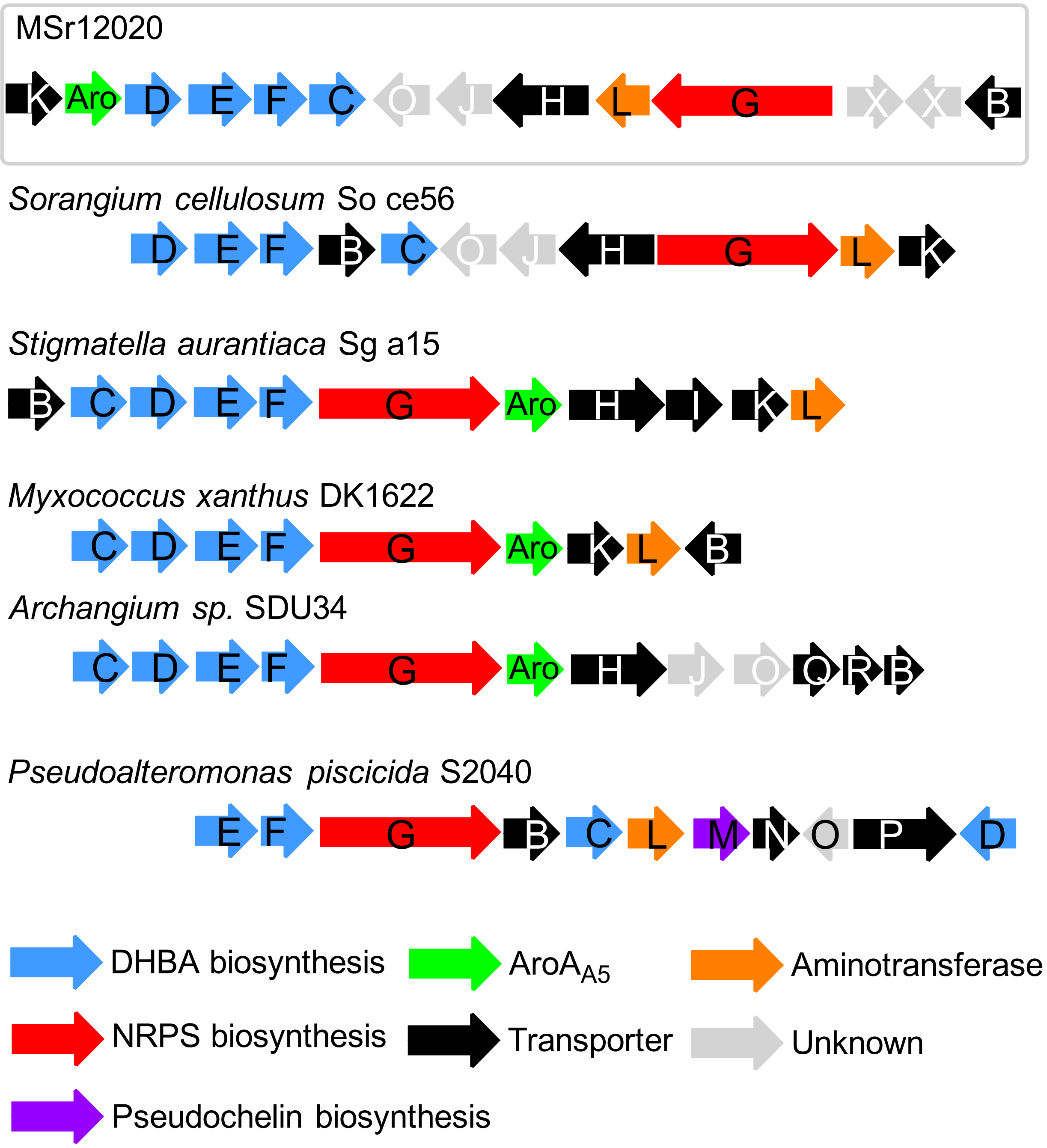

Figure S13. Comparison of *mxc* BGCs that have been characterized to produce different myxochelins in different myxobacterial strains and *Pseudomonasalteromonas piscicida*. Genes were depicted in different colors according to biological functions.

4. Metabolome database search of 1–3

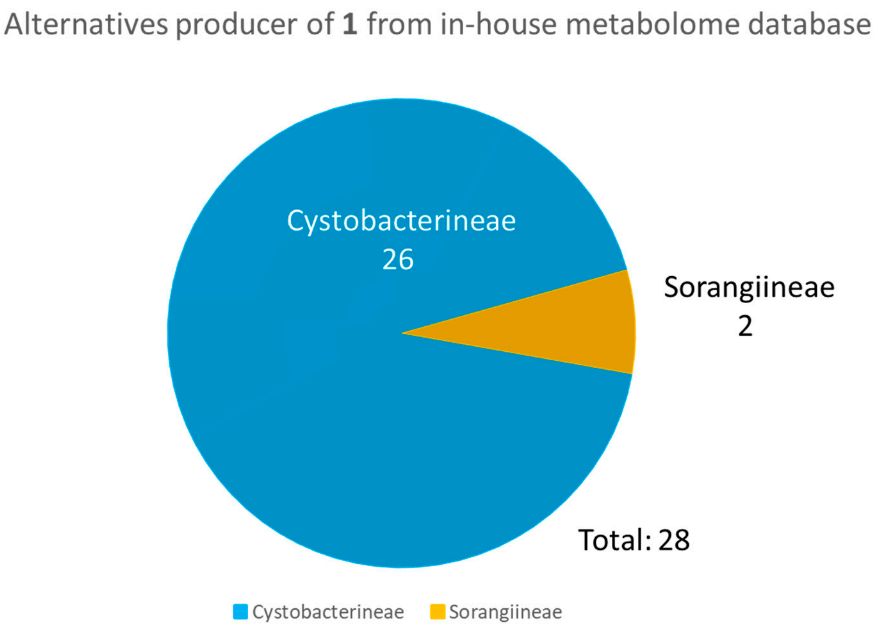

Figure S14. Alternative producers of **1** from our in-house metabolome database sorted by suborder. Search parameters: exact mass deviation <5ppm, retention time deviation <0.3 min, area threshold $3*10^4$. The production of **1** was always co-occurring with the production of **2** and **3**.

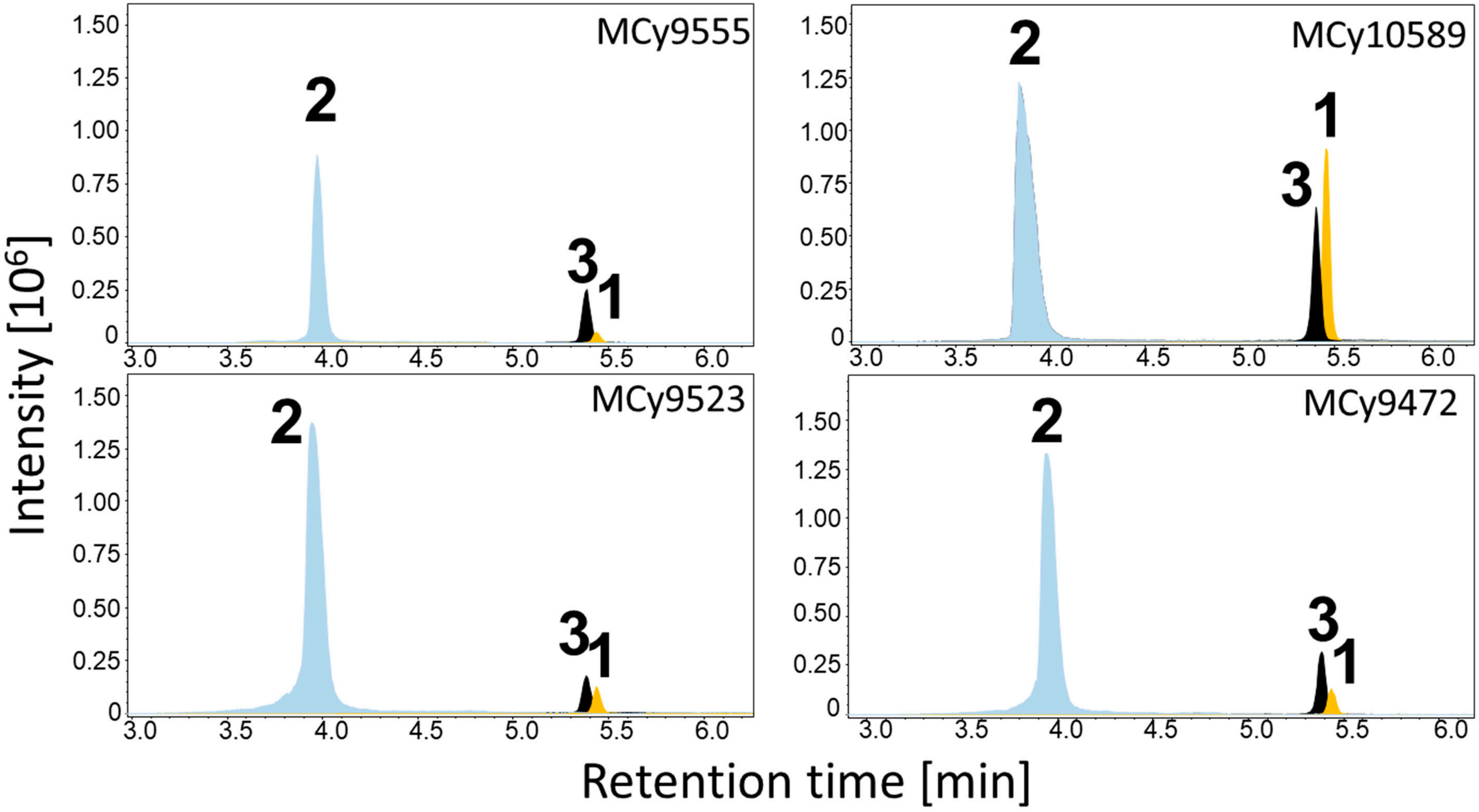

Figure S15. High performance liquid chromatography–mass spectrometry extracted ion chromatograms (HPLC–MS EIC) of **1** orange ($[M+H]^+$ 504.1976 m/z, (orange), **2** ($[M+H]^+$ 404.1819 m/z, blue), and **3** ($[M+H]^+$ 405.1658 m/z, black) from the myxobacterial crude extracts of MCy9555, MCy10589, MCy9523 and MCy9472.

5. ^{1}H and ^{13}C NMR spectra

microorganisms

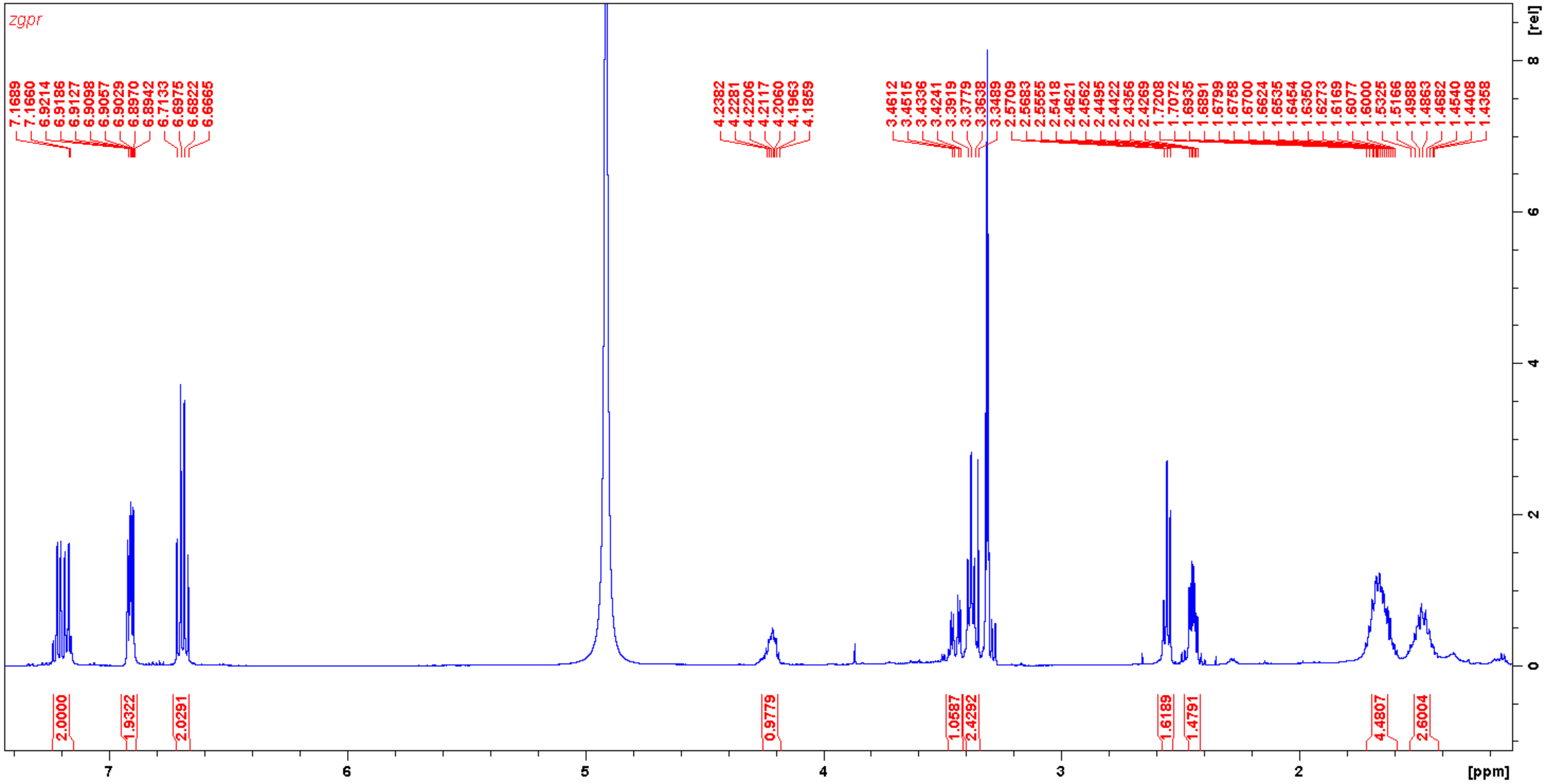

Figure S16. ^{1}H NMR of compound **1**.

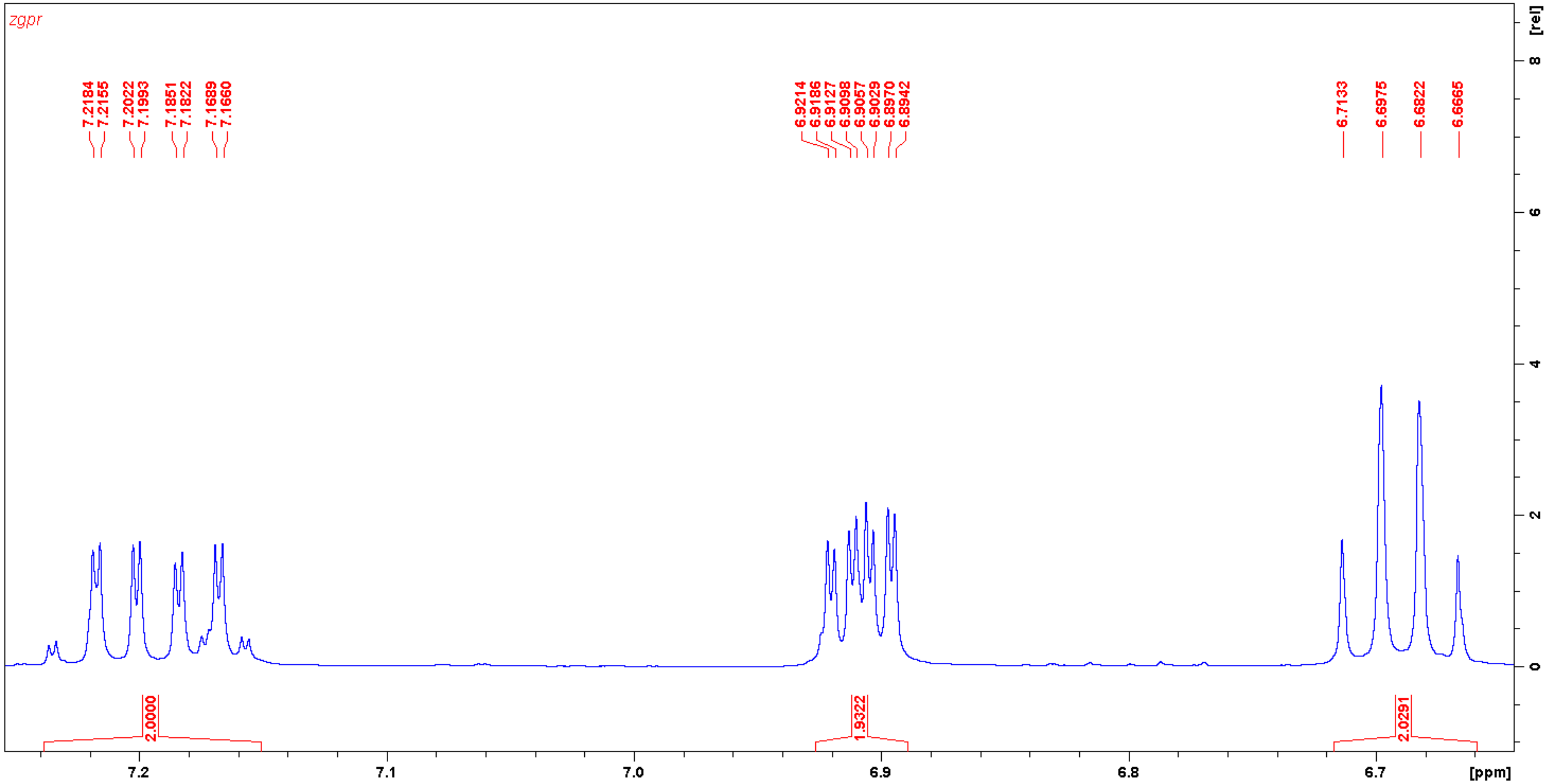

Figure S17. ^{1}H NMR spectrum of compound **1** (expanded part 1).

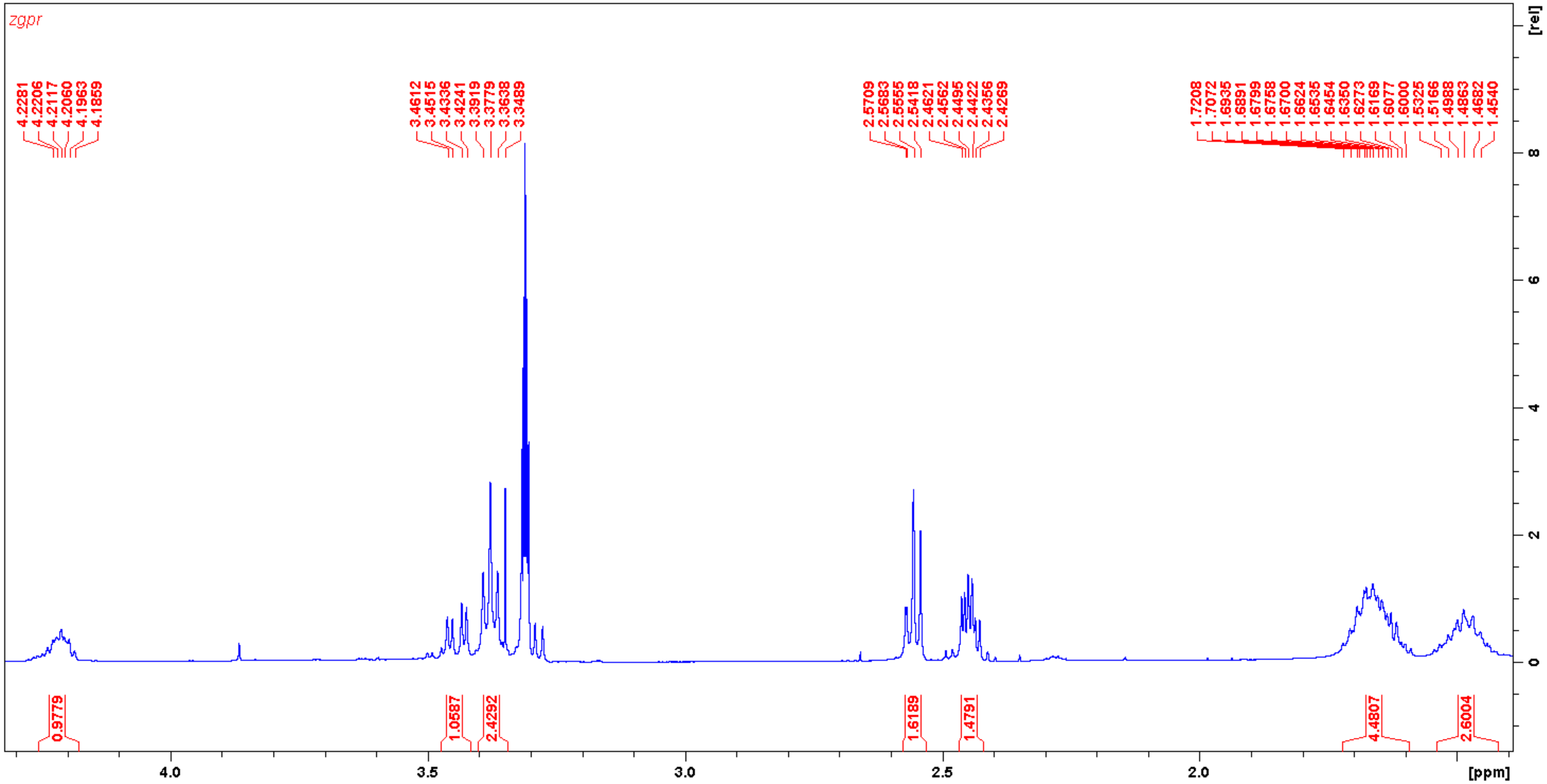

Figure S18. ^{1}H NMR spectrum of compound **1** (expanded part 2).

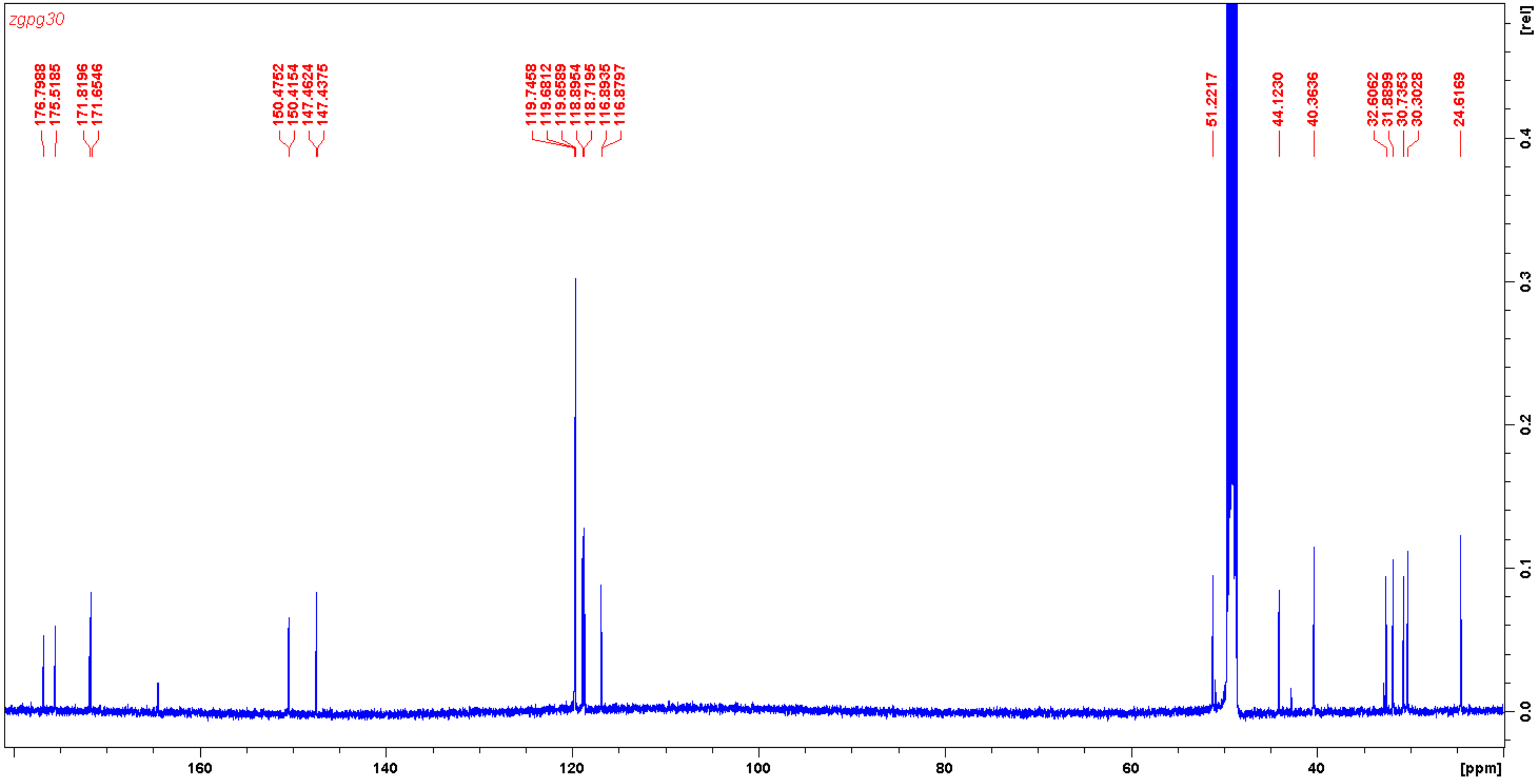

Figure S19. ^{13}C NMR spectrum of compound **1**.

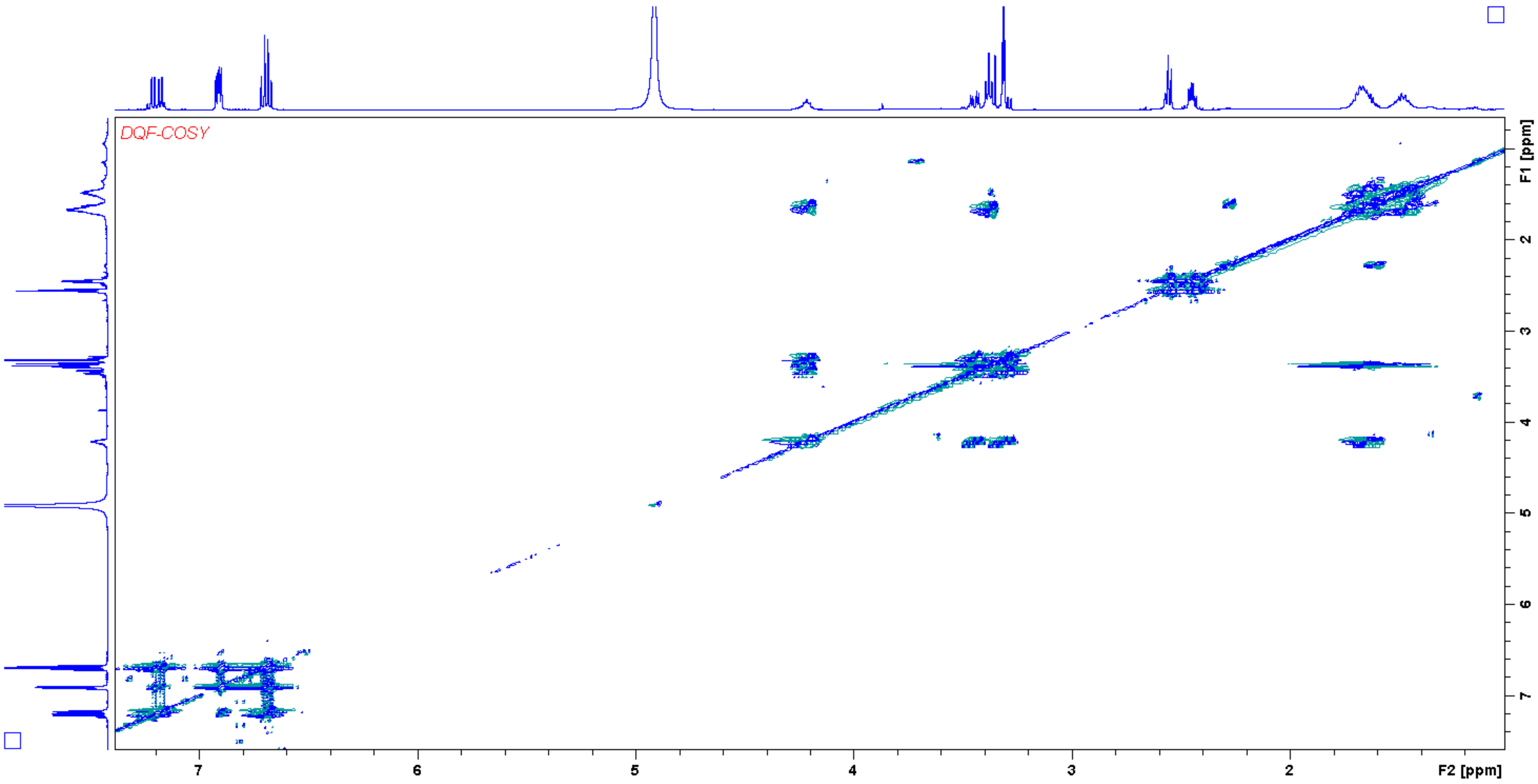

Figure S20. ^{1}H-^{1}H DQF-COSY spectrum of compound **1**.

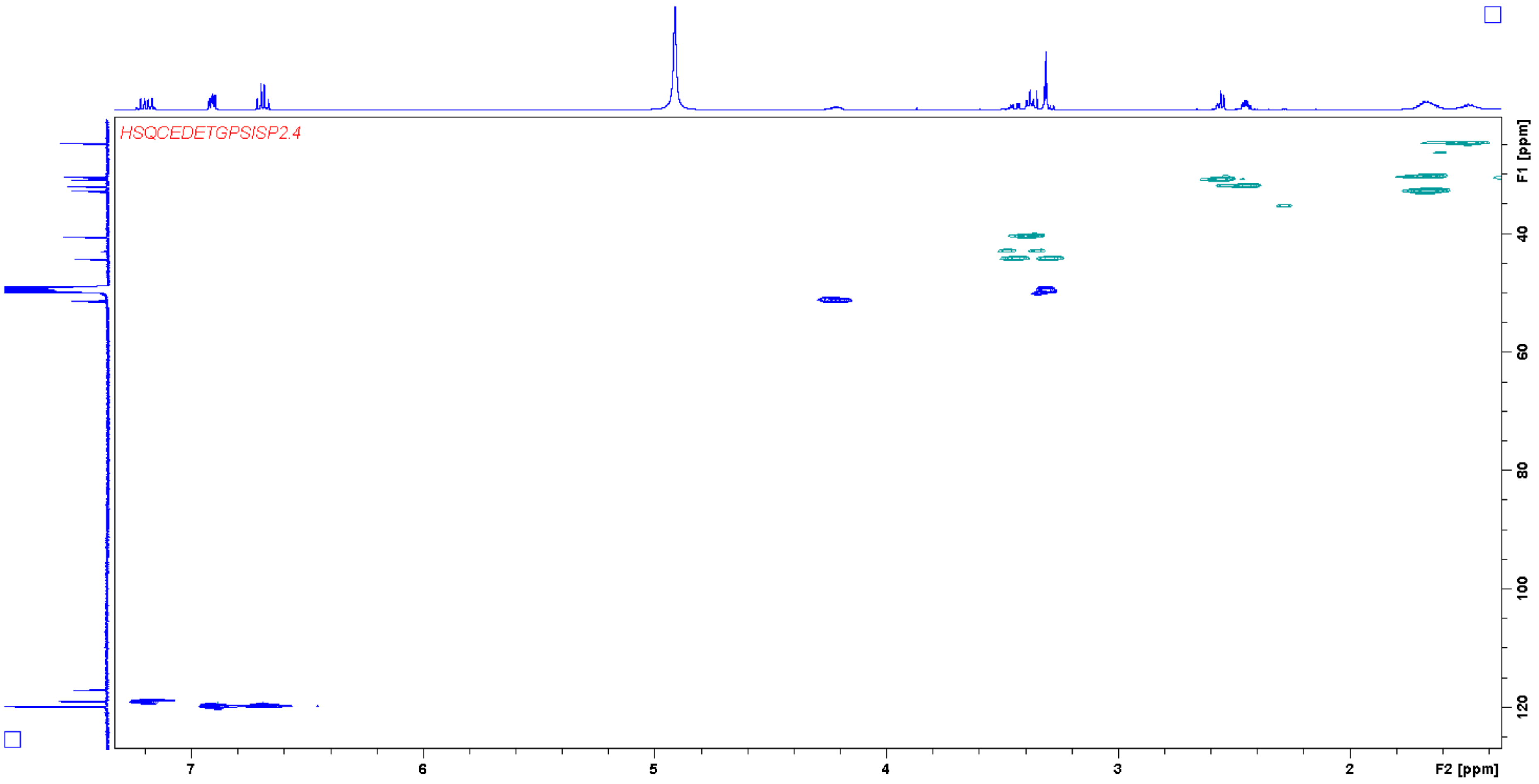

Figure S21. HSQC spectrum of compound **1**.

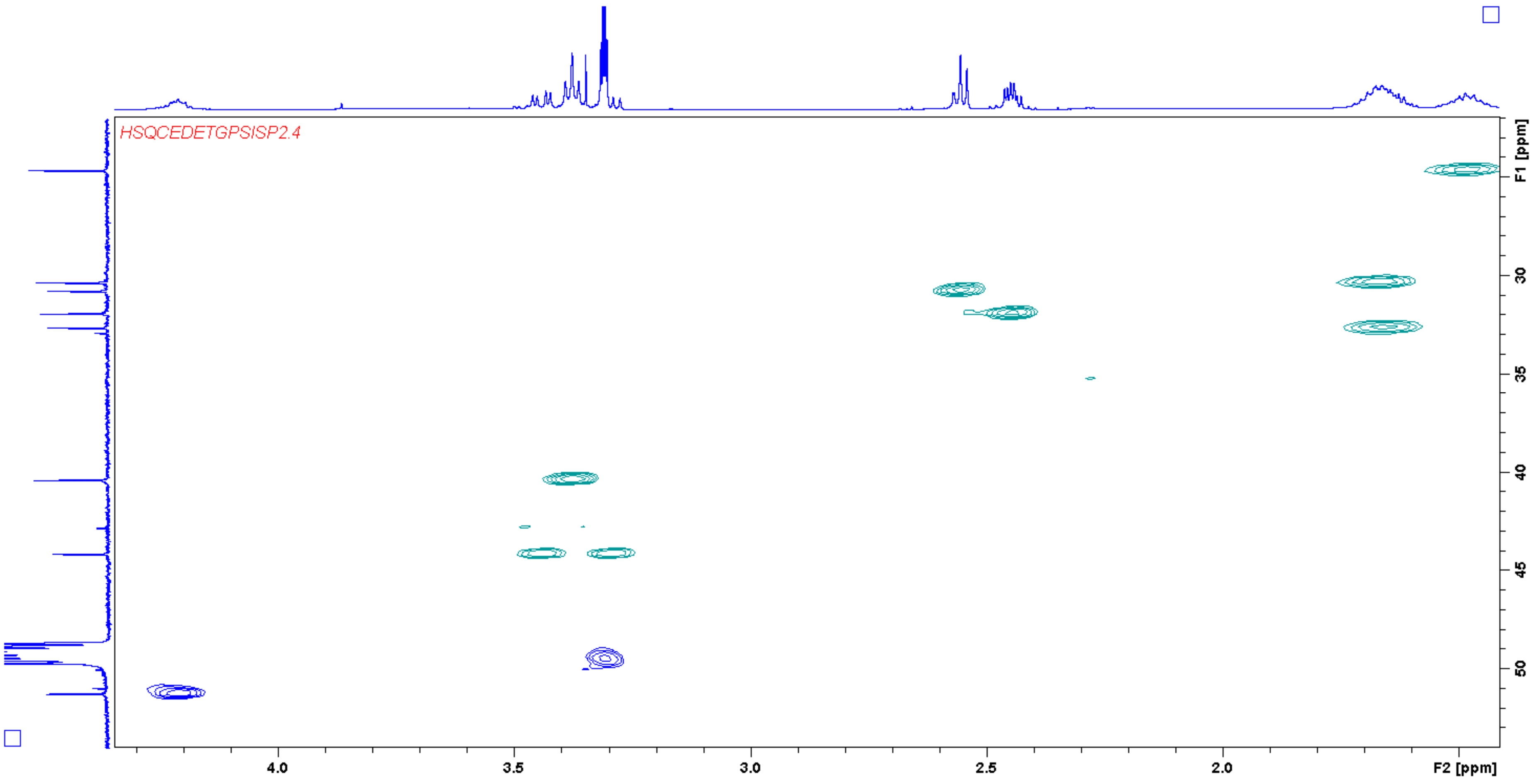

Figure S22. HSQC spectrum of compound **1** (expanded).

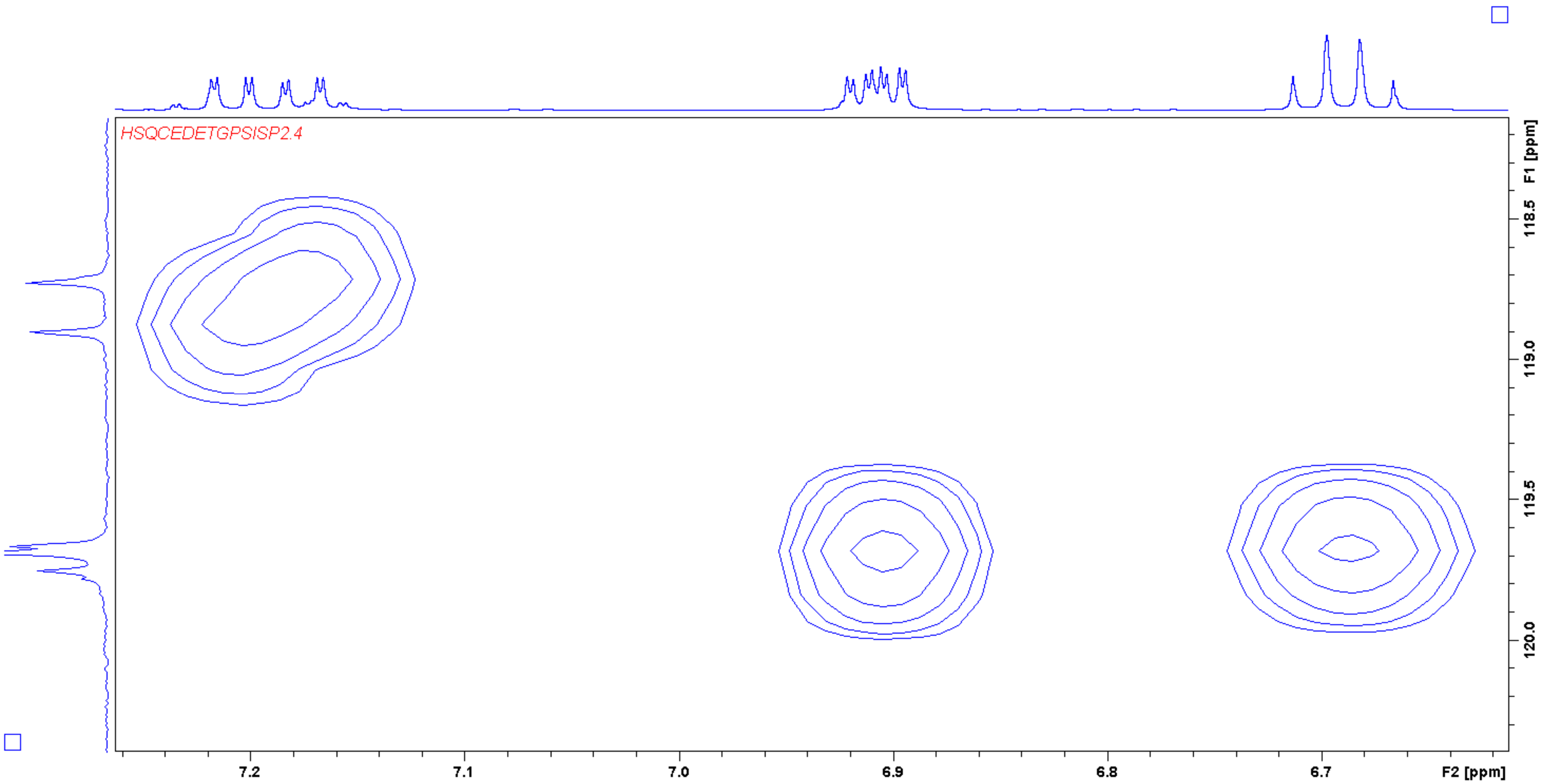

Figure S23. HSQC spectrum of compound **1** (expanded).

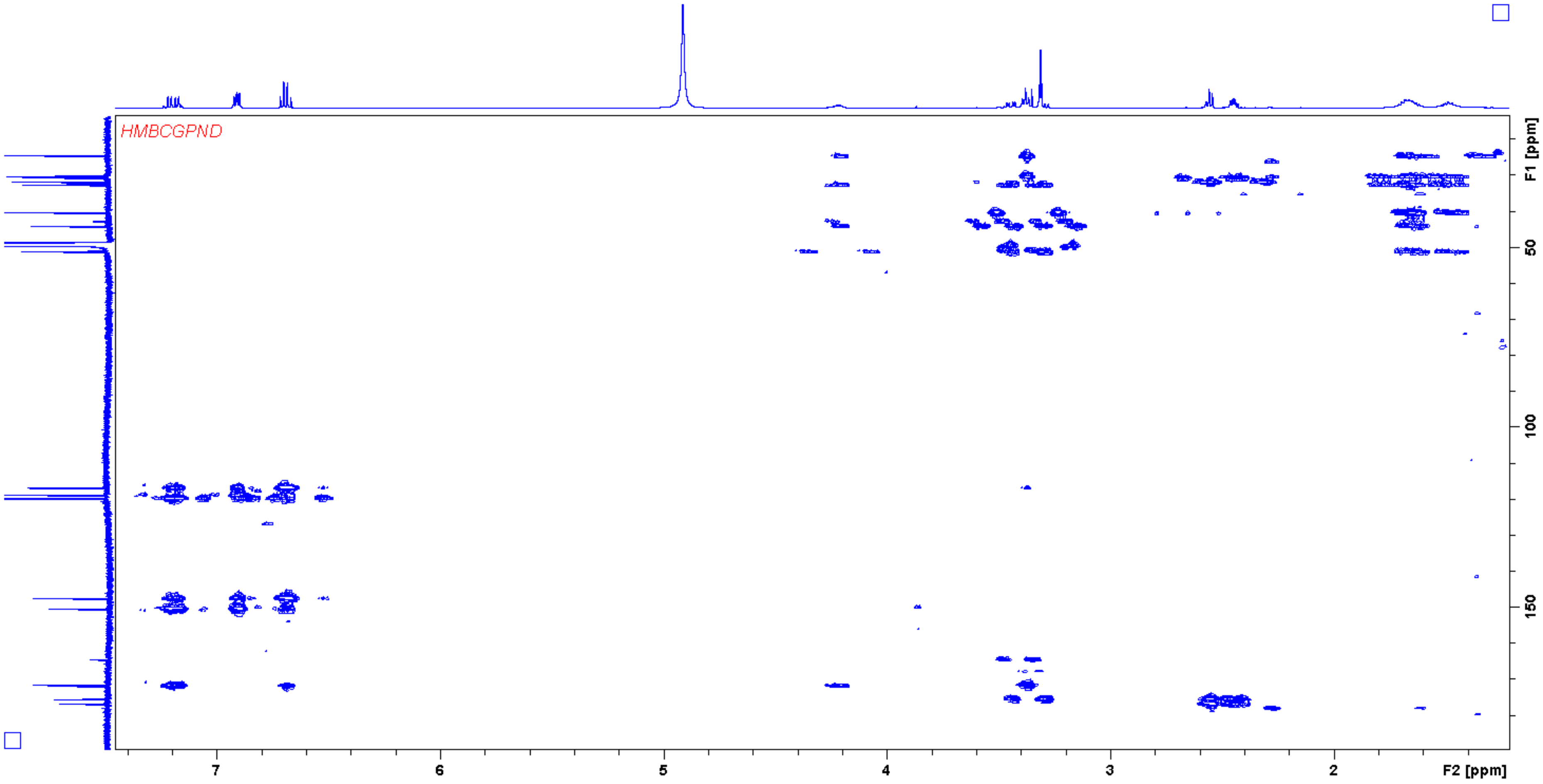

Figure S24. ^{1}H-^{13}C HMBC spectrum of compound **1**.

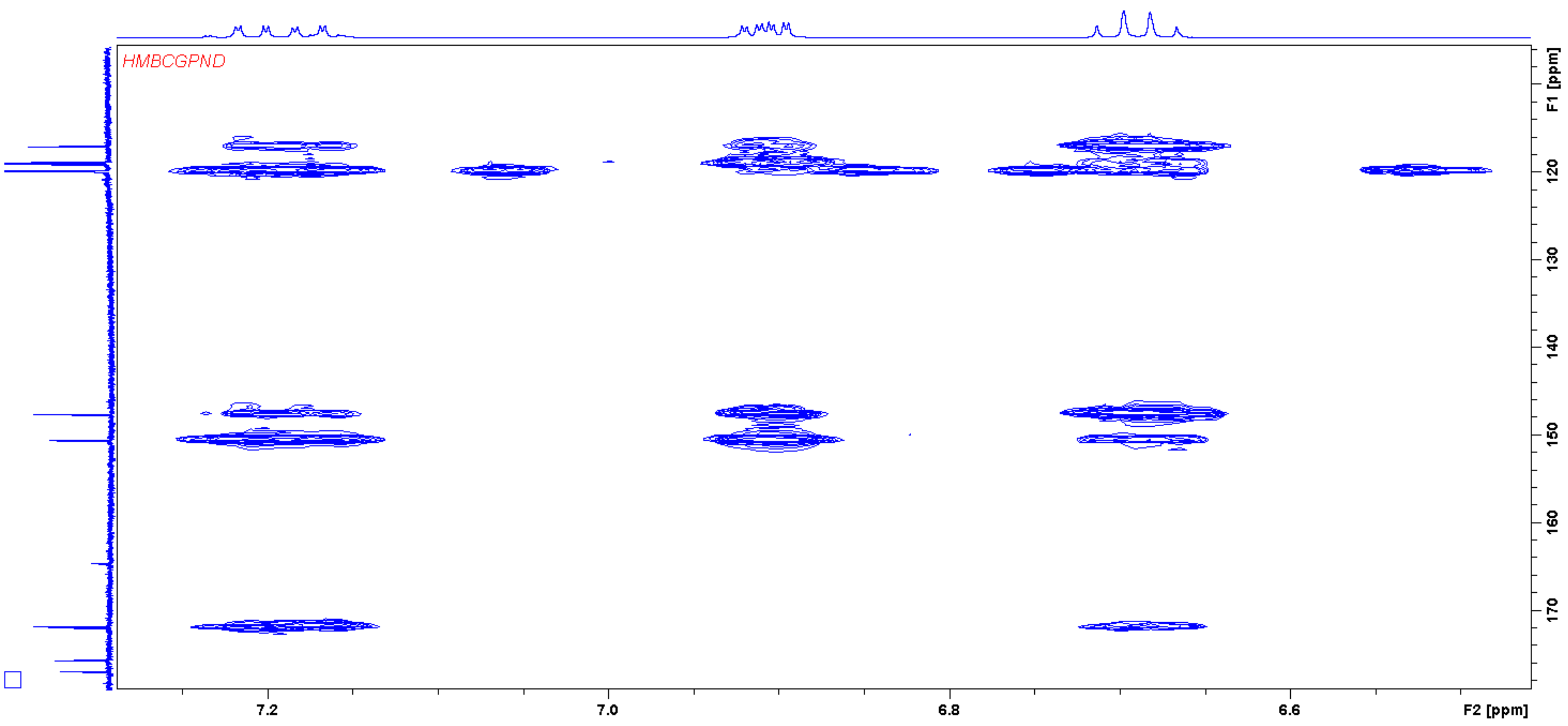

Figure S25. ^{1}H-^{13}C HMBC spectrum of compound **1** (expanded part 1).

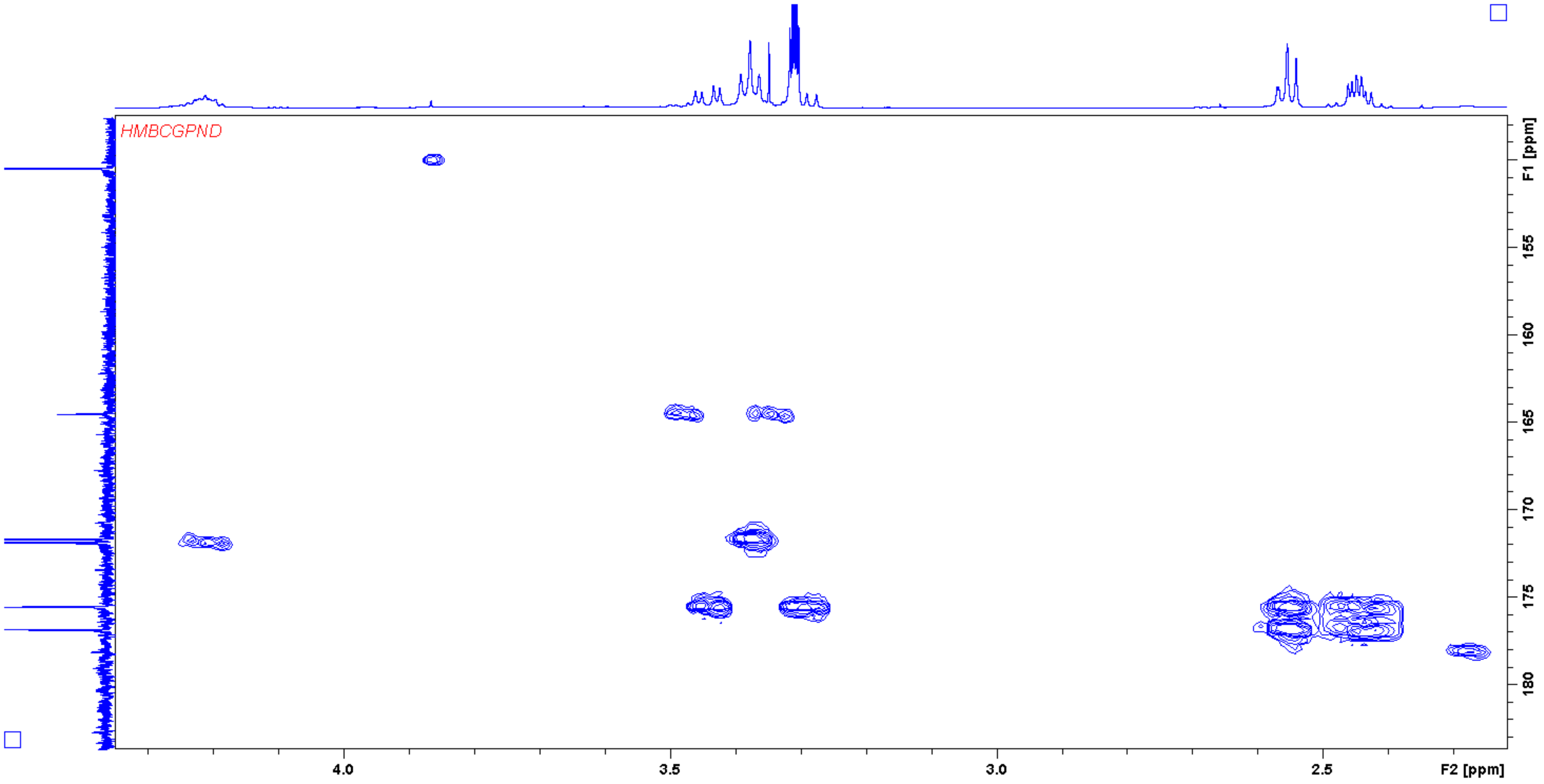

Figure S26. ^{1}H-^{13}C HMBC spectrum of compound **1** (expanded part 2).

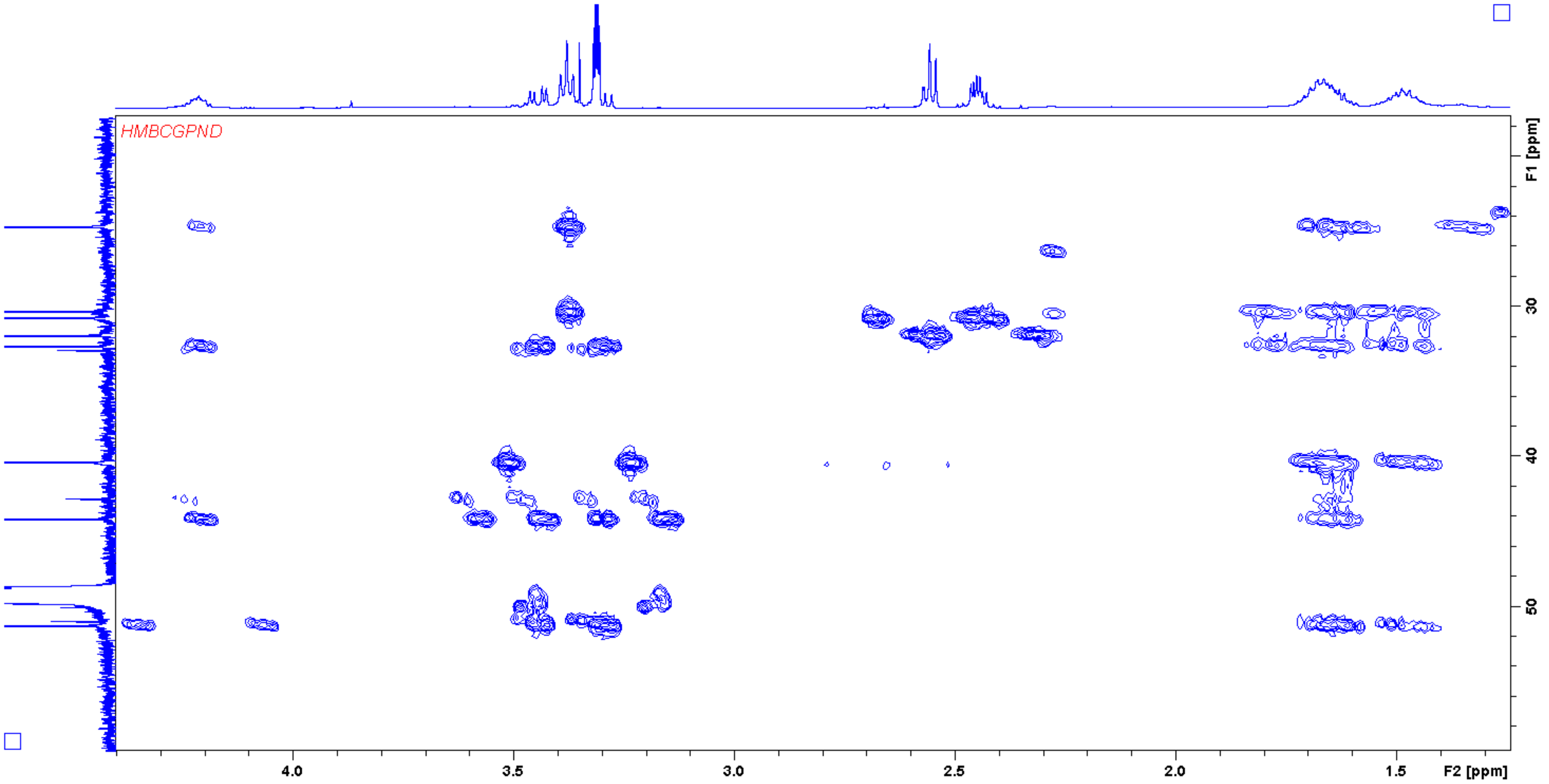

Figure S27. ^{1}H-^{13}C HMBC spectrum of compound **1** (expanded part 3).

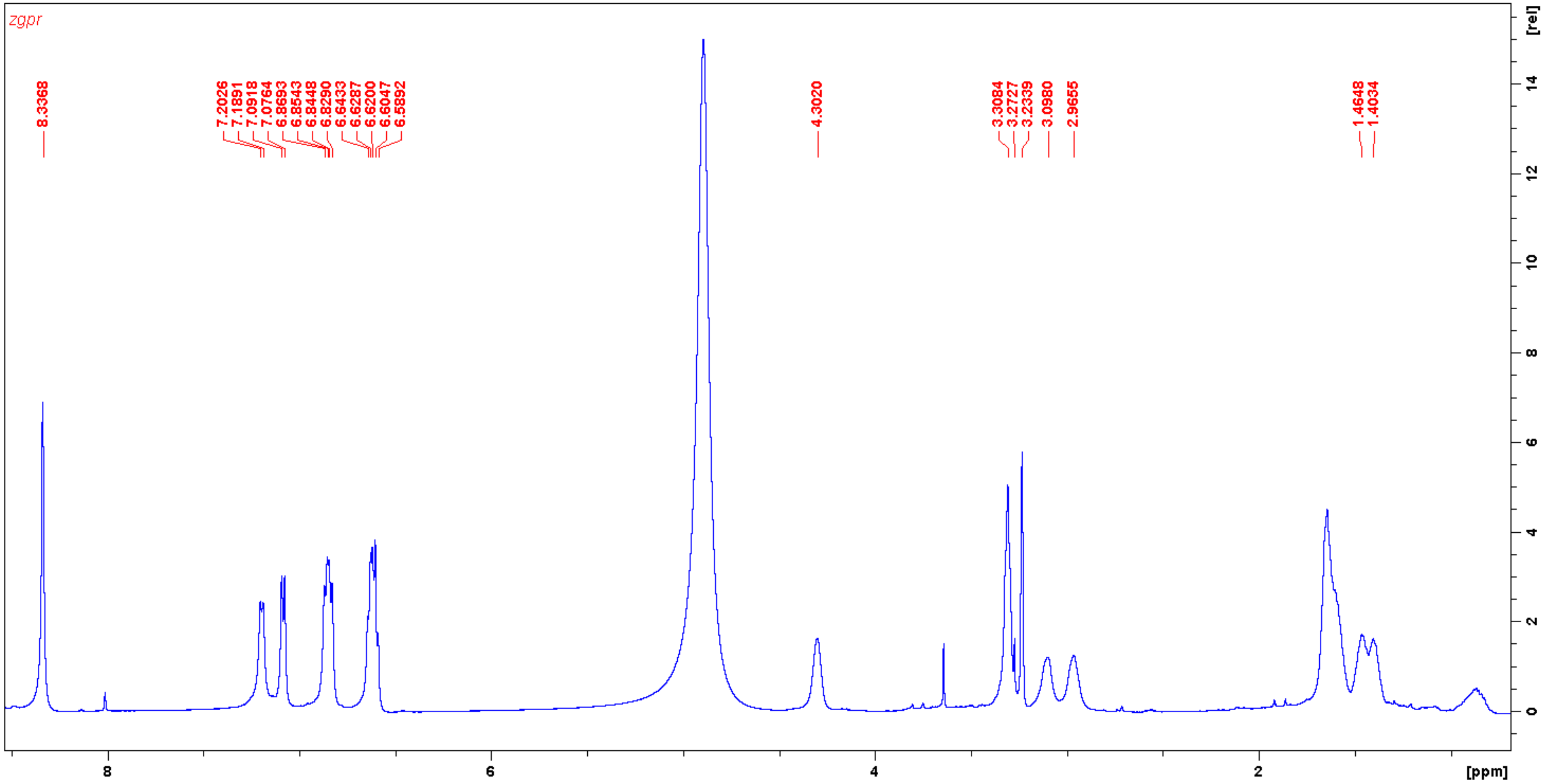

Figure S28. ^{1}H NMR spectrum of compound **2**.

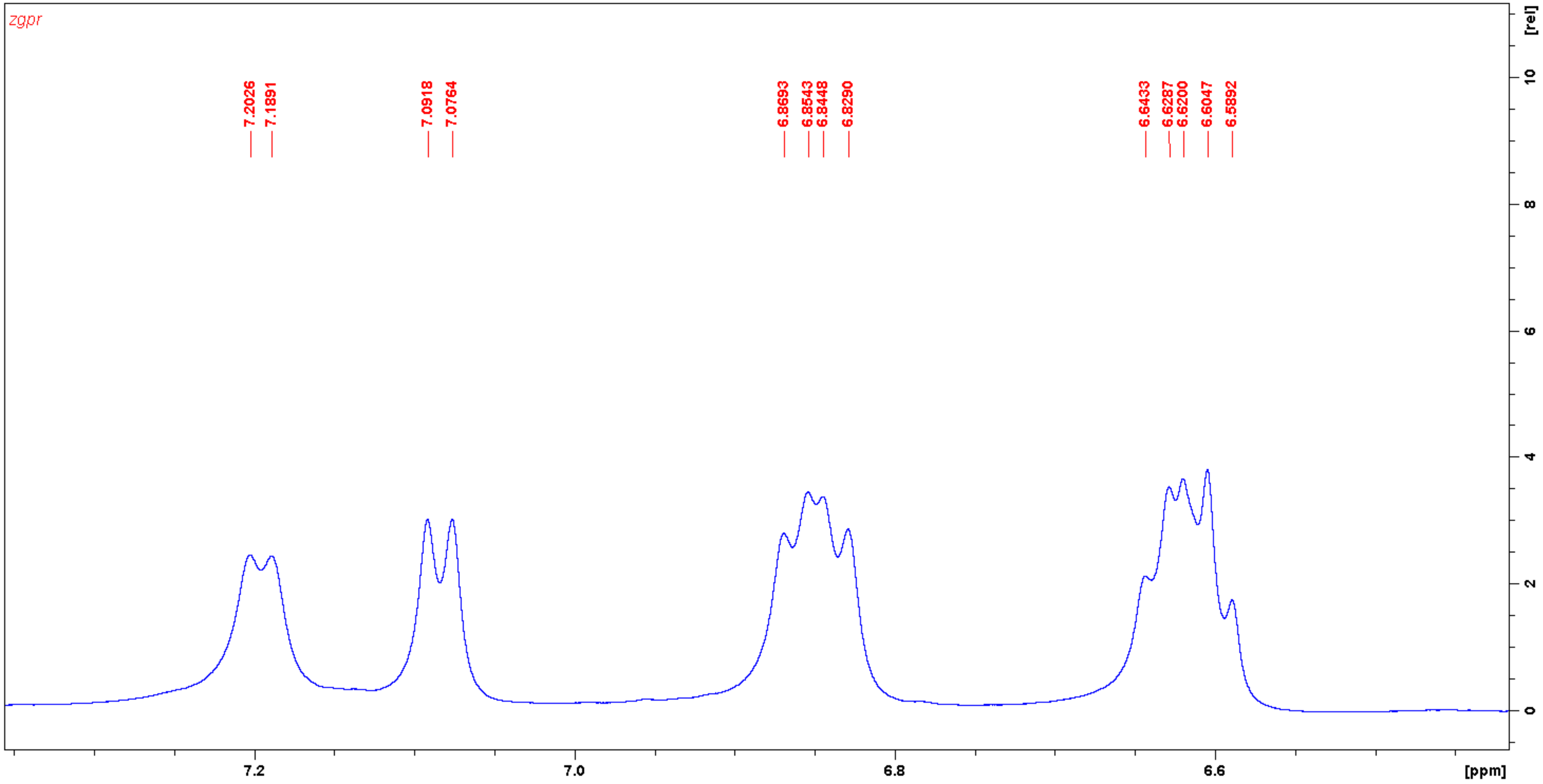

Figure S29. ^{1}H NMR spectrum of compound **2** (expanded part 1).

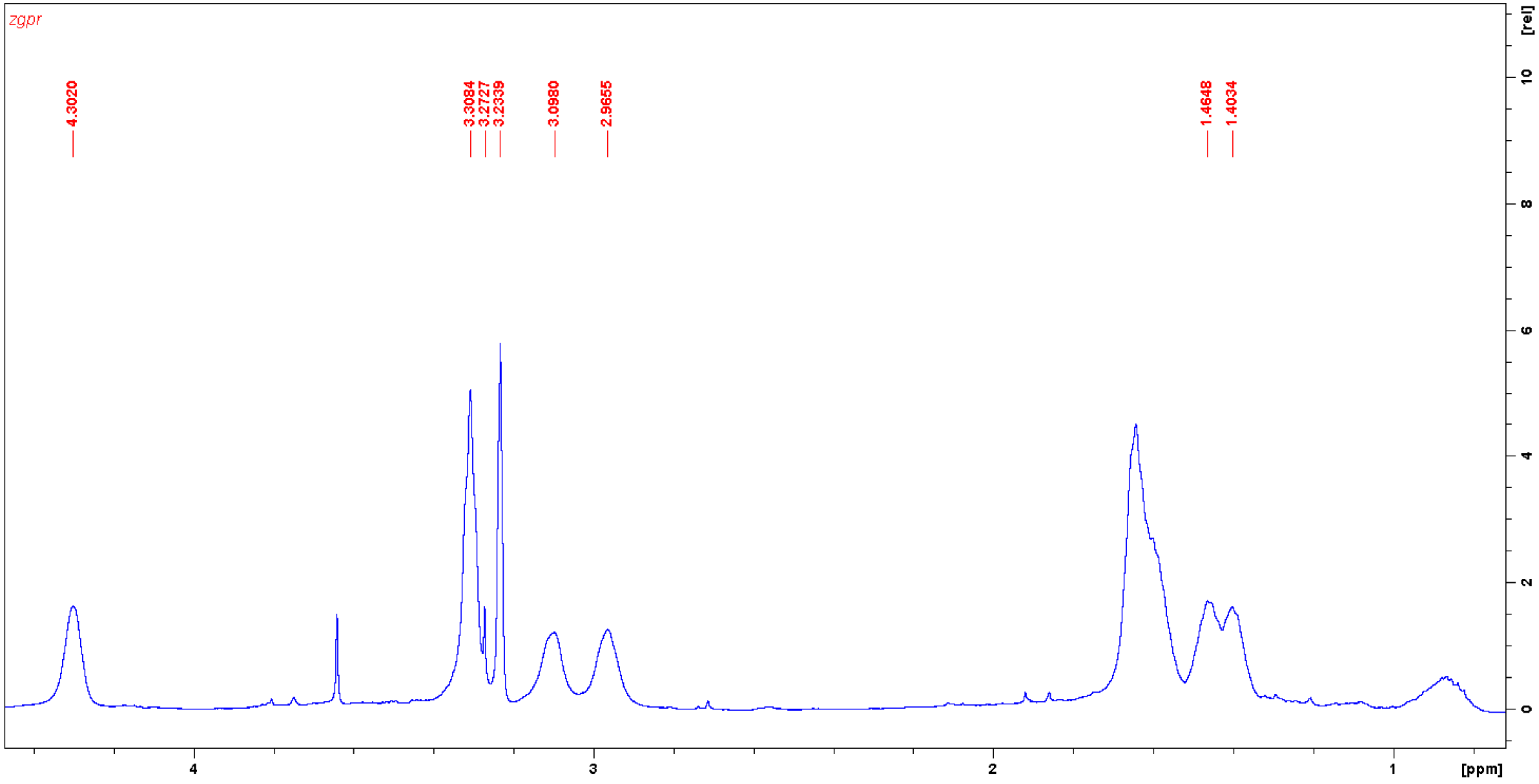

Figure S30. ^{1}H NMR spectrum of compound **2** (expanded part 2).

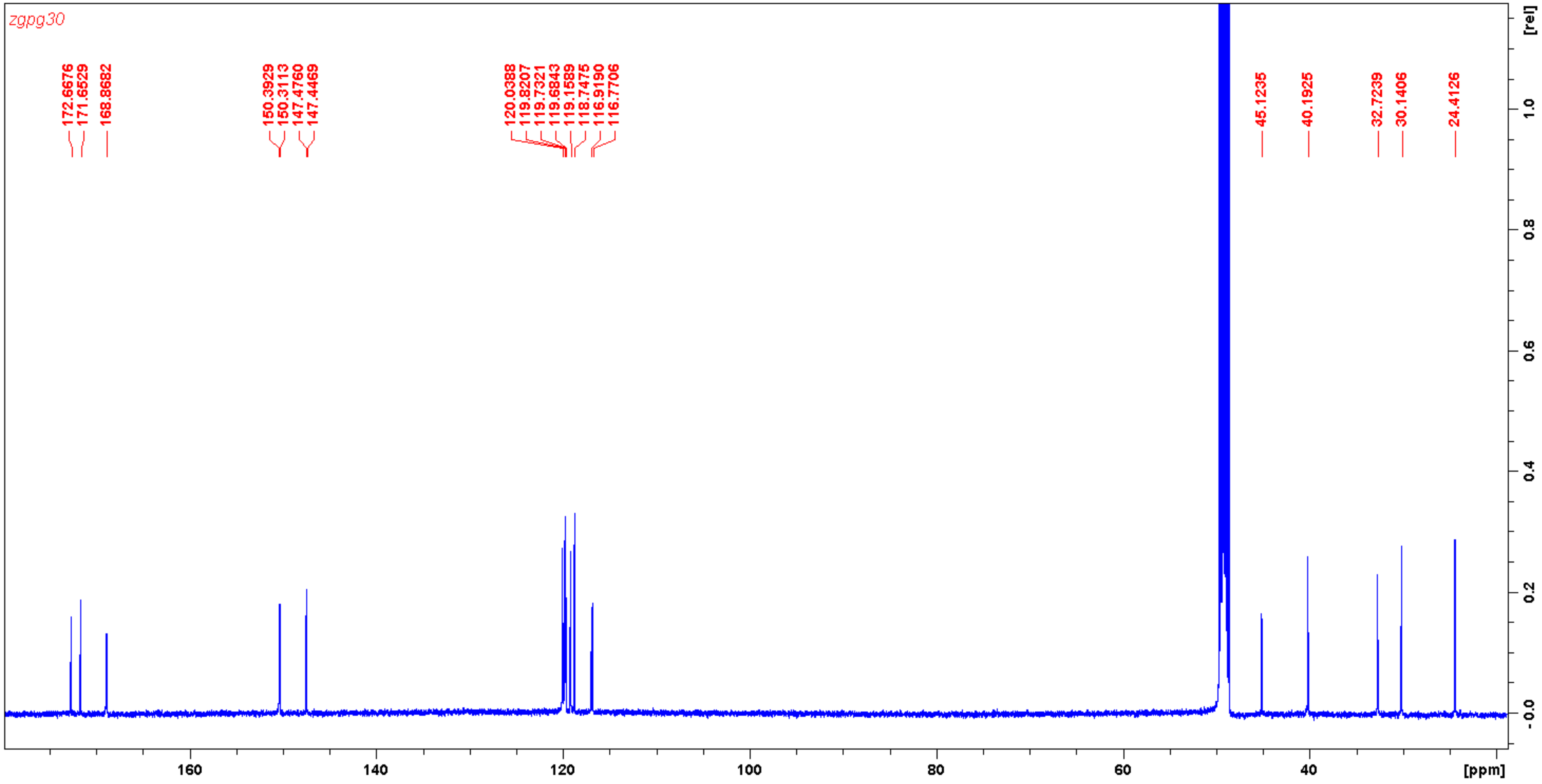

Figure S31. ^{13}C NMR spectrum of compound **2** in MeOD.

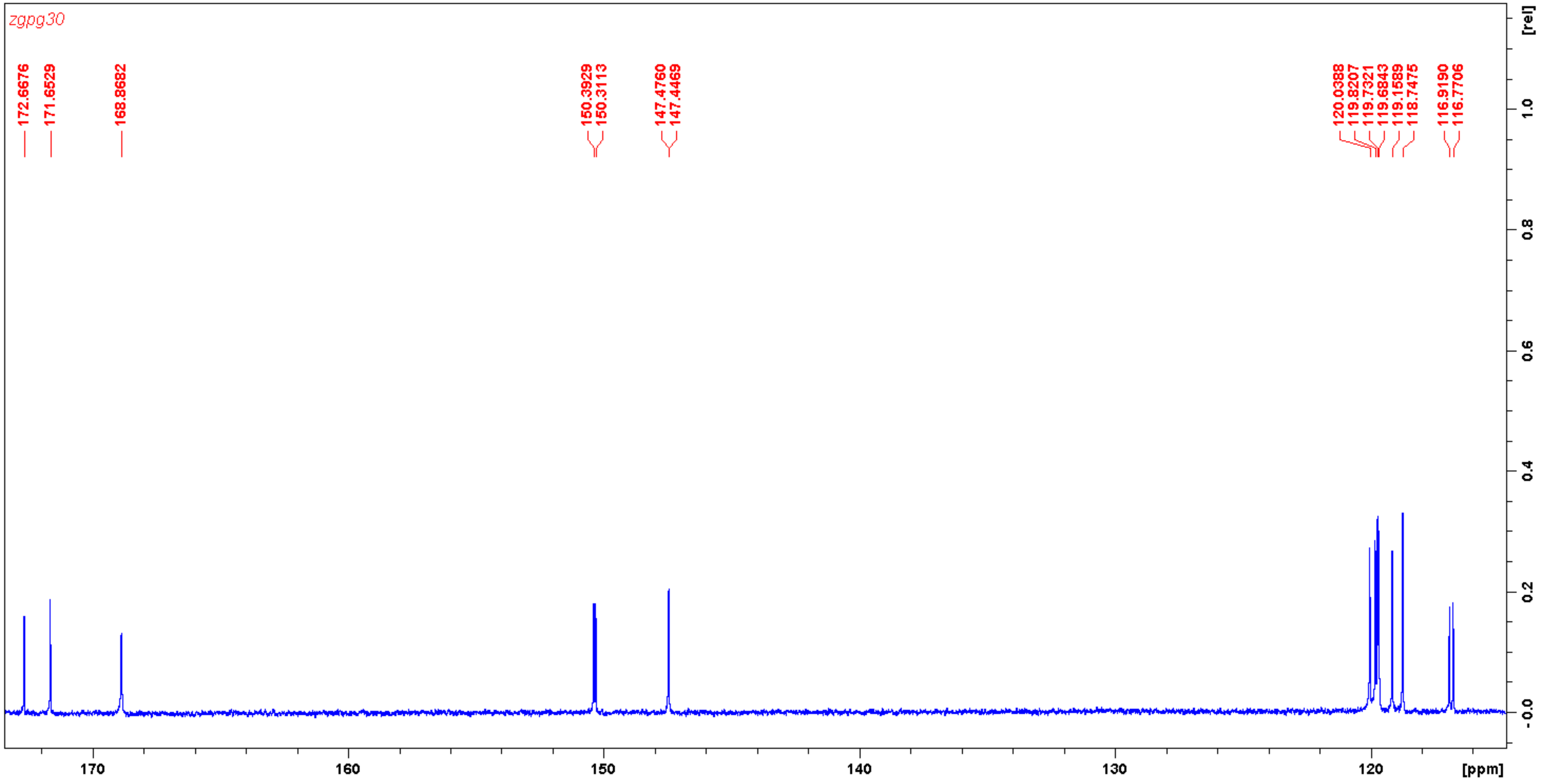

Figure S32. ^{13}C NMR spectrum of compound **2** (expanded part 1).

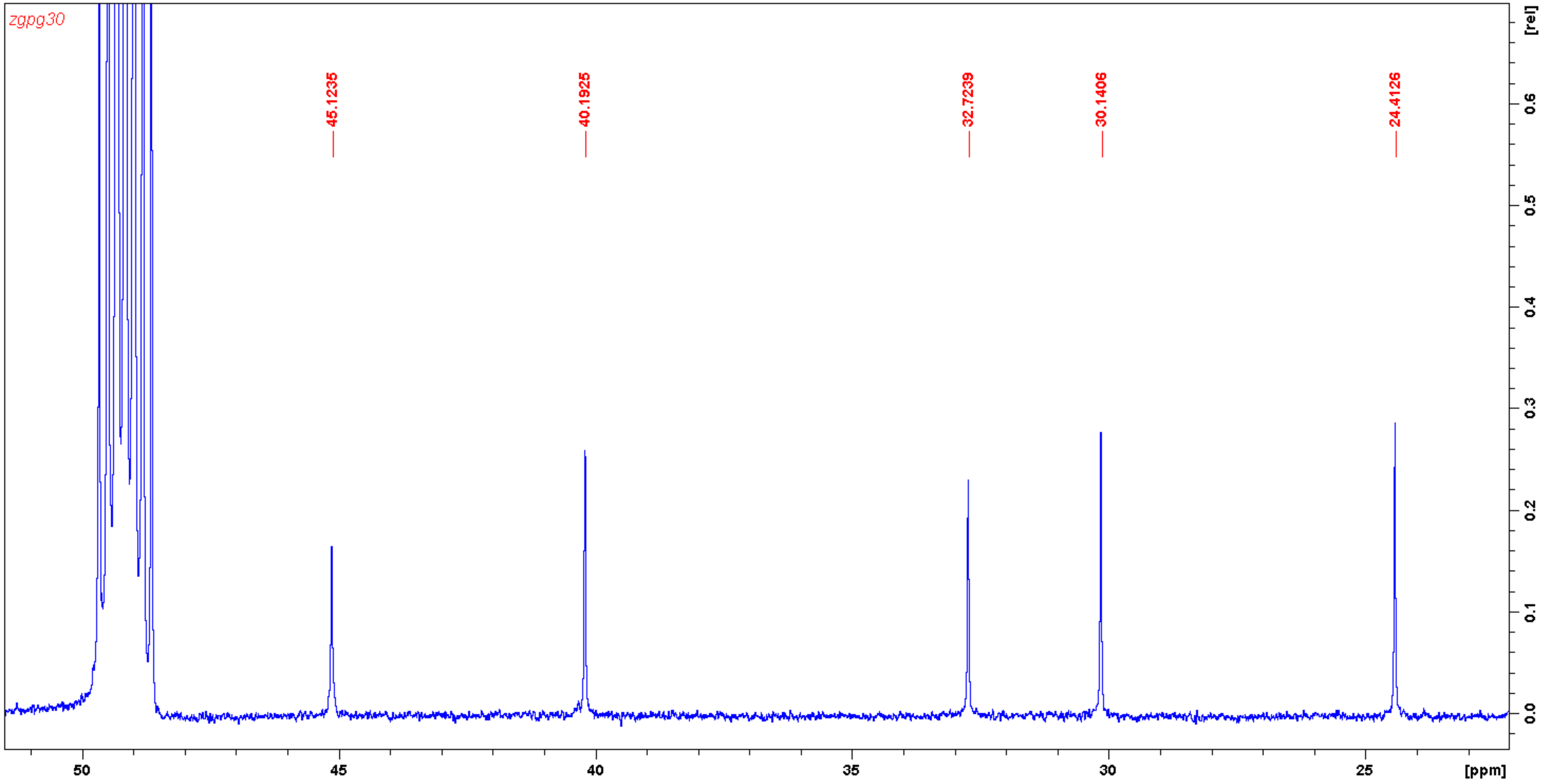

Figure S33. ^{13}C NMR spectrum of compound **2** (expanded part 2).

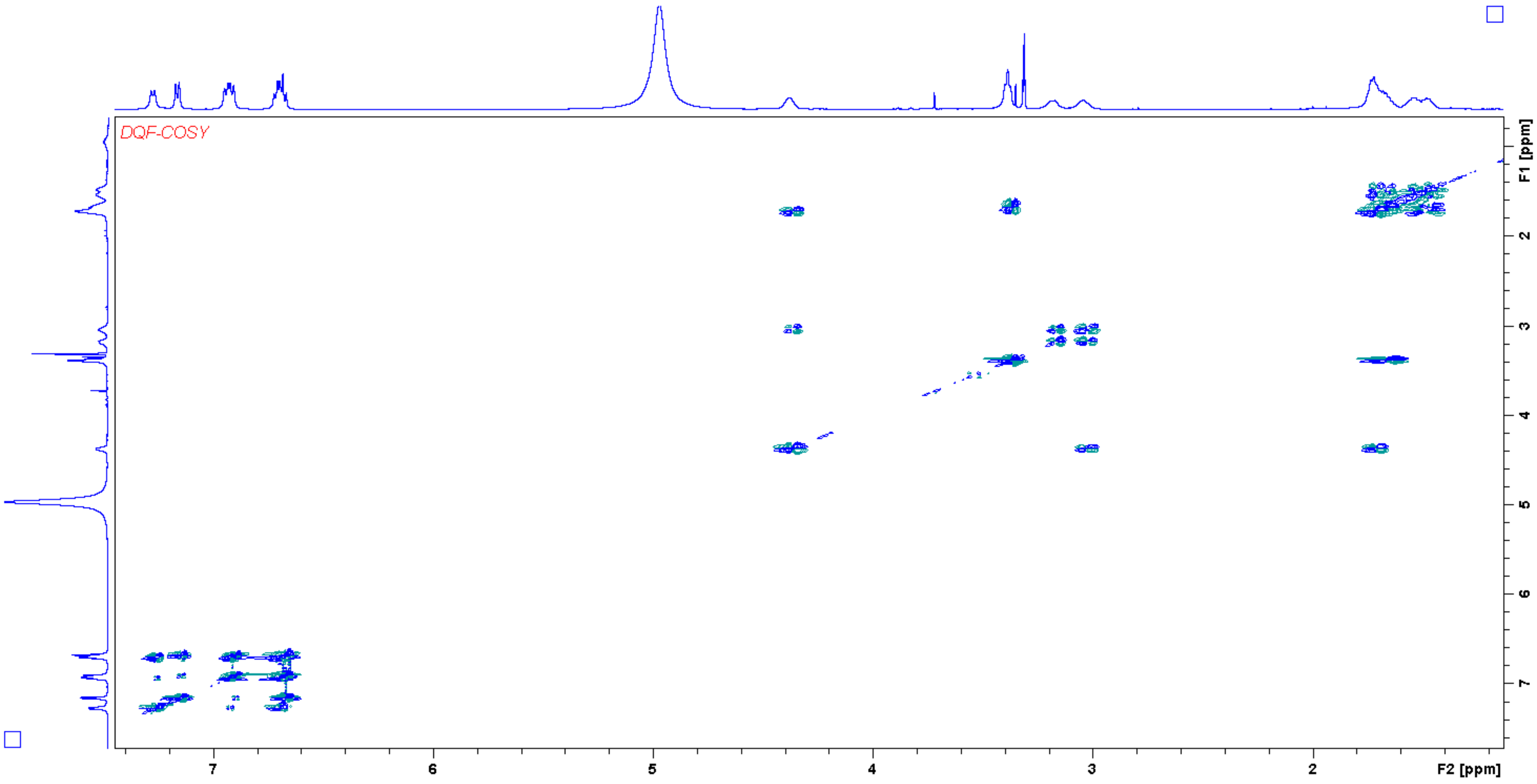

Figure S34. ^{1}H-^{1}H DQF-COSY spectrum of compound **2**.

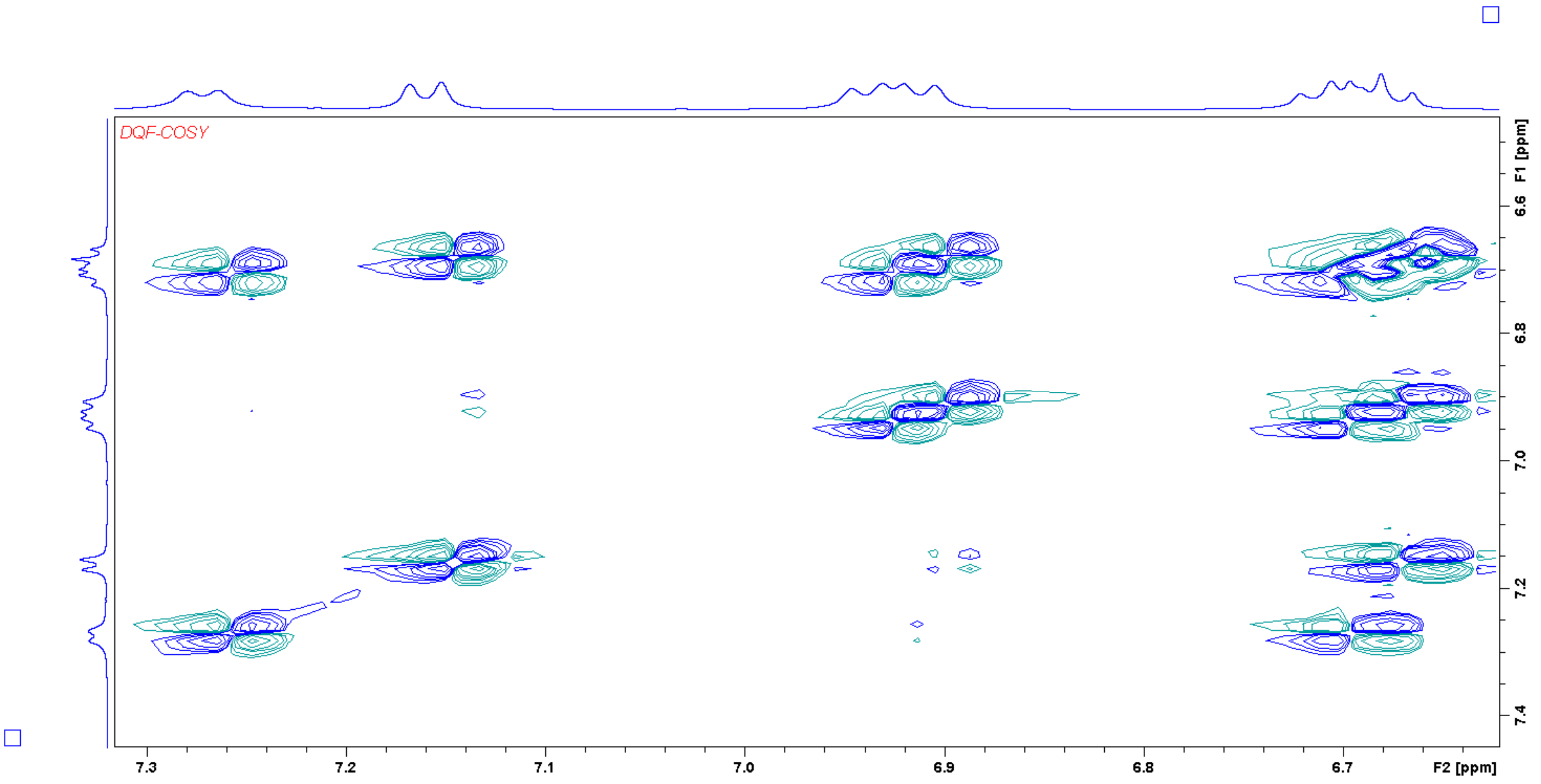

Figure S35. ^{1}H-^{1}H DQF-COSY spectrum of compound **2**.

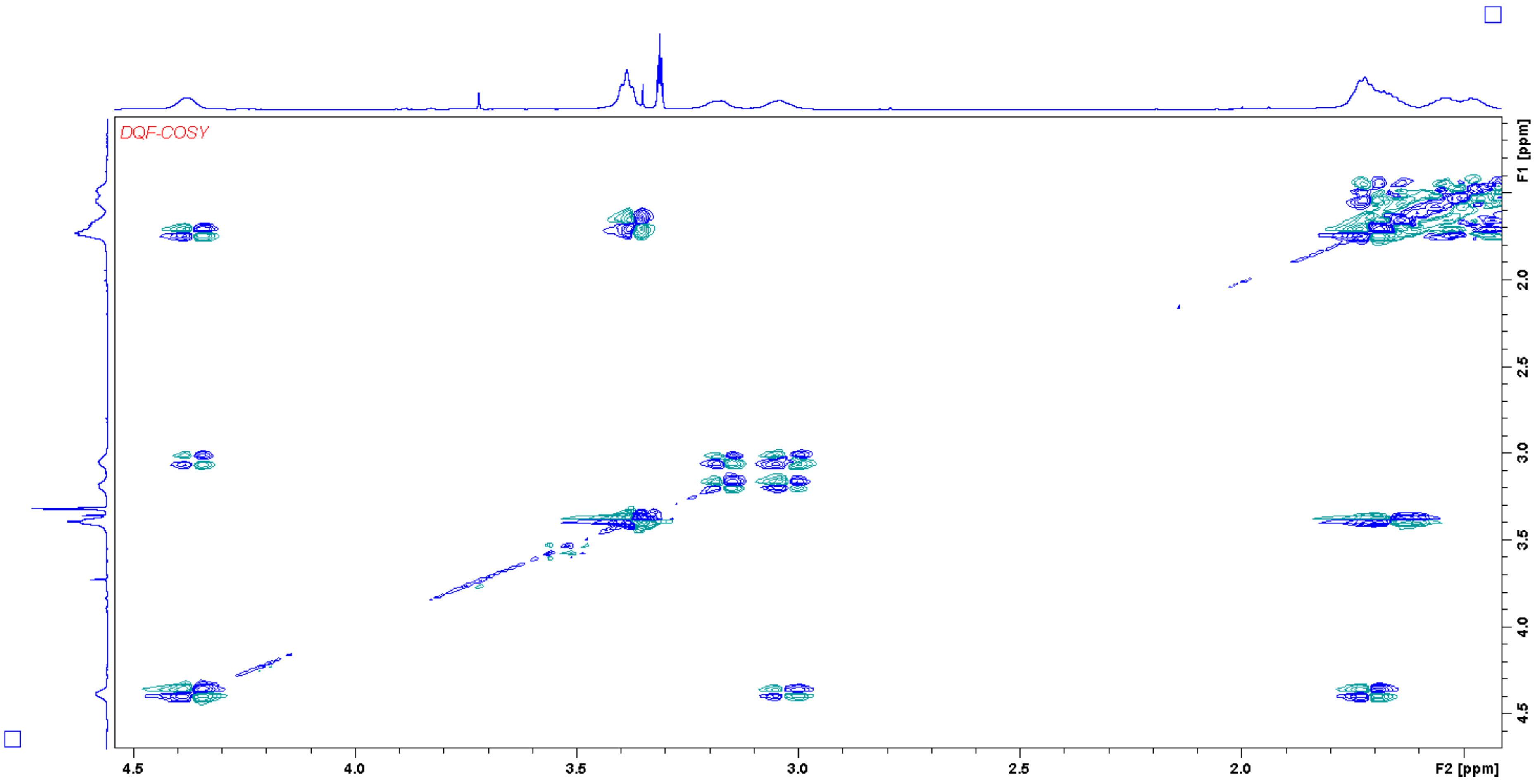

Figure S36. ^{1}H-^{1}H DQF-COSY spectrum of compound **2** (expanded).

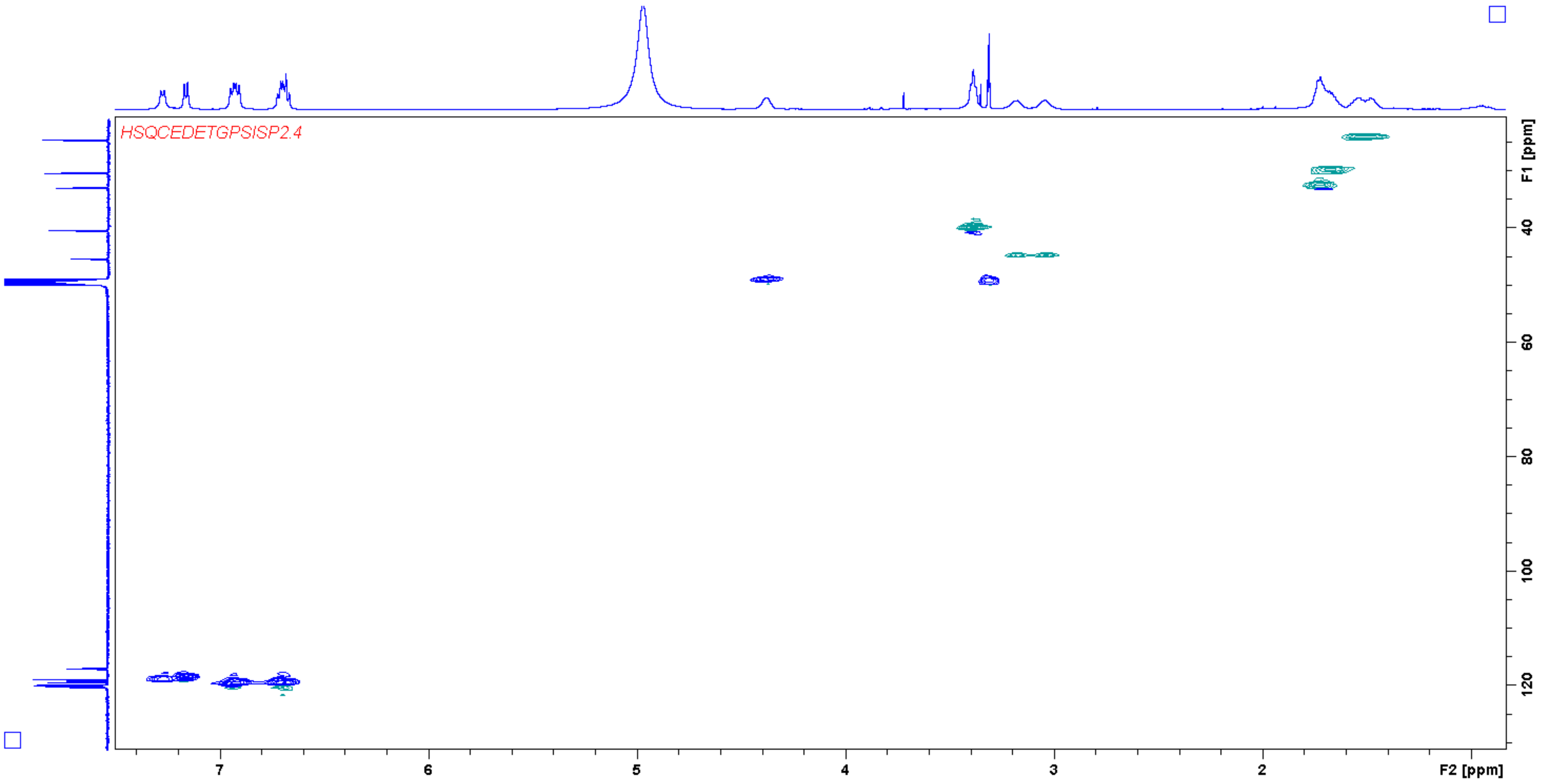

Figure S37. HSQC spectrum of compound **2**.

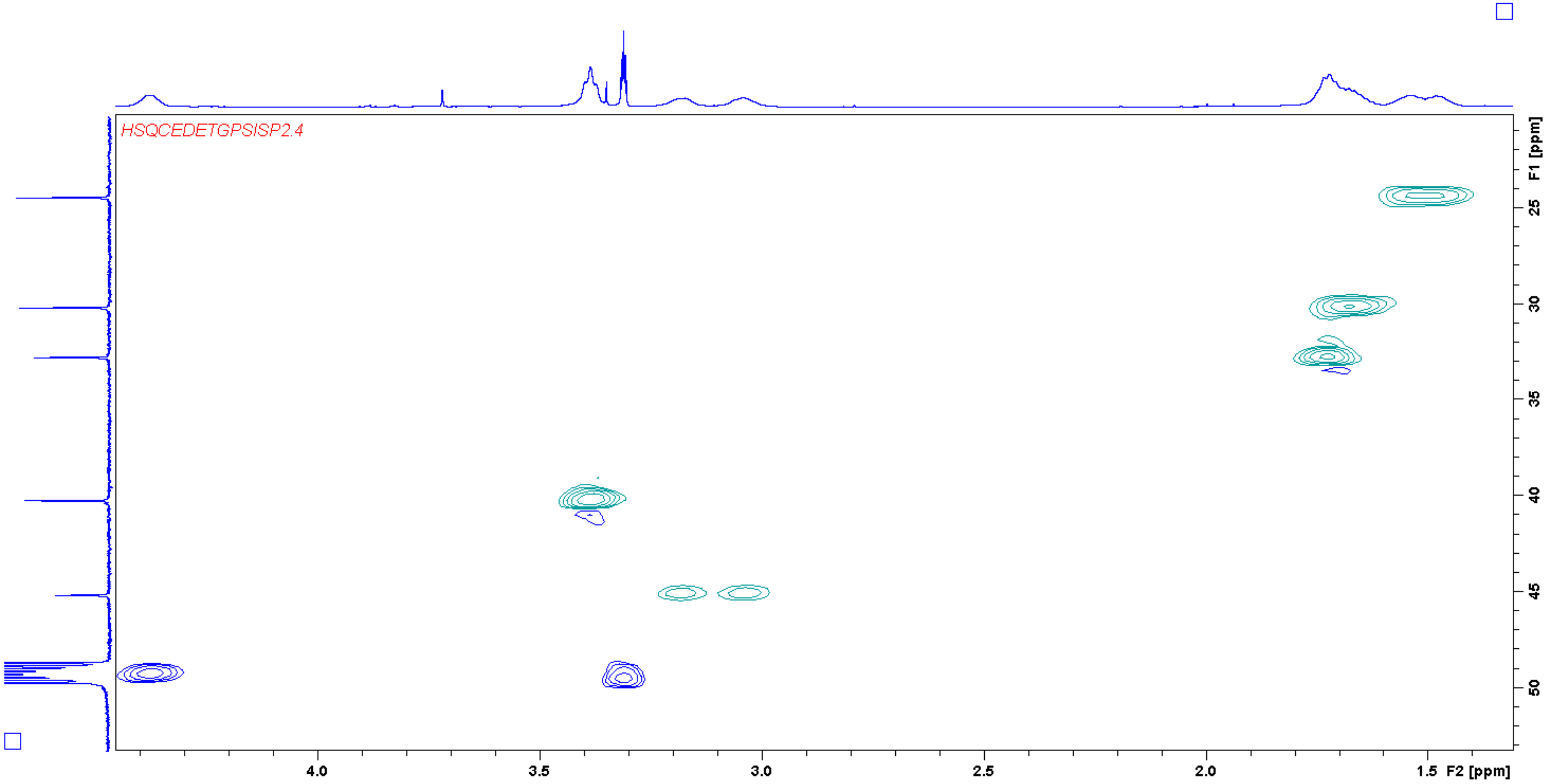

Figure S38. HSQC spectrum of compound **2** (expanded part 1).

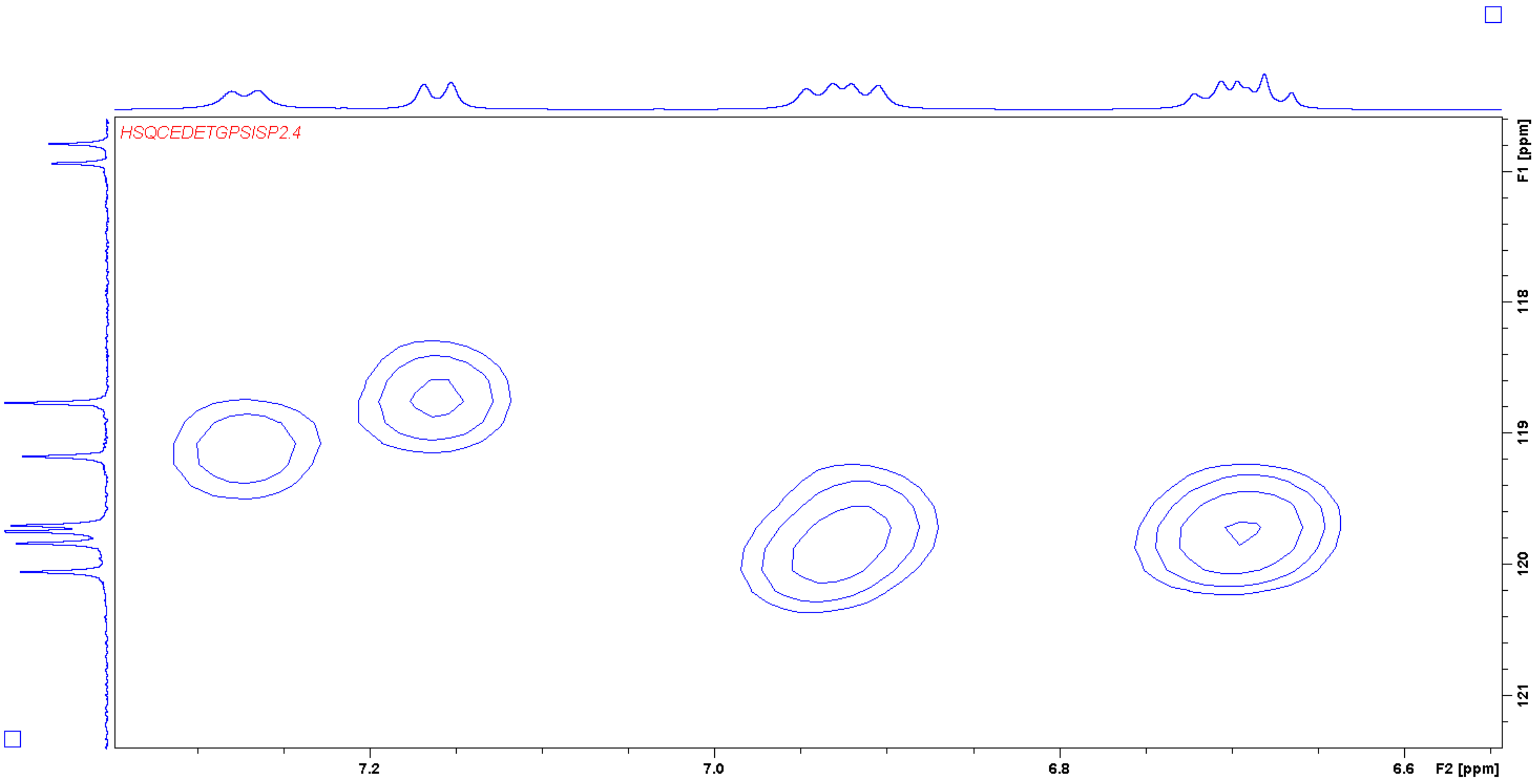

Figure S39. HSQC spectrum of compound **2** (expanded part 2).

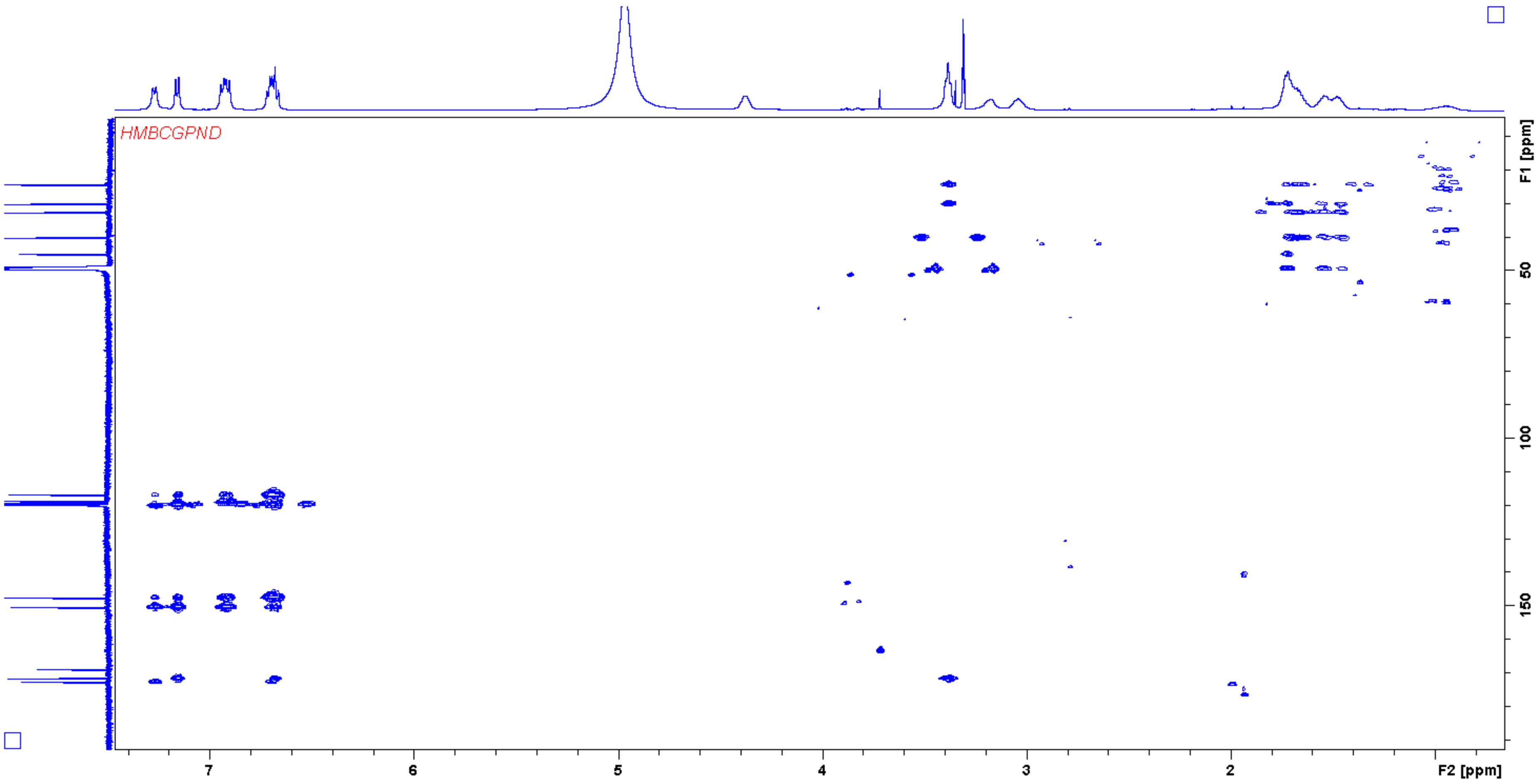

Figure S40. ^{1}H-^{13}C HMBC spectrum of compound **2**.

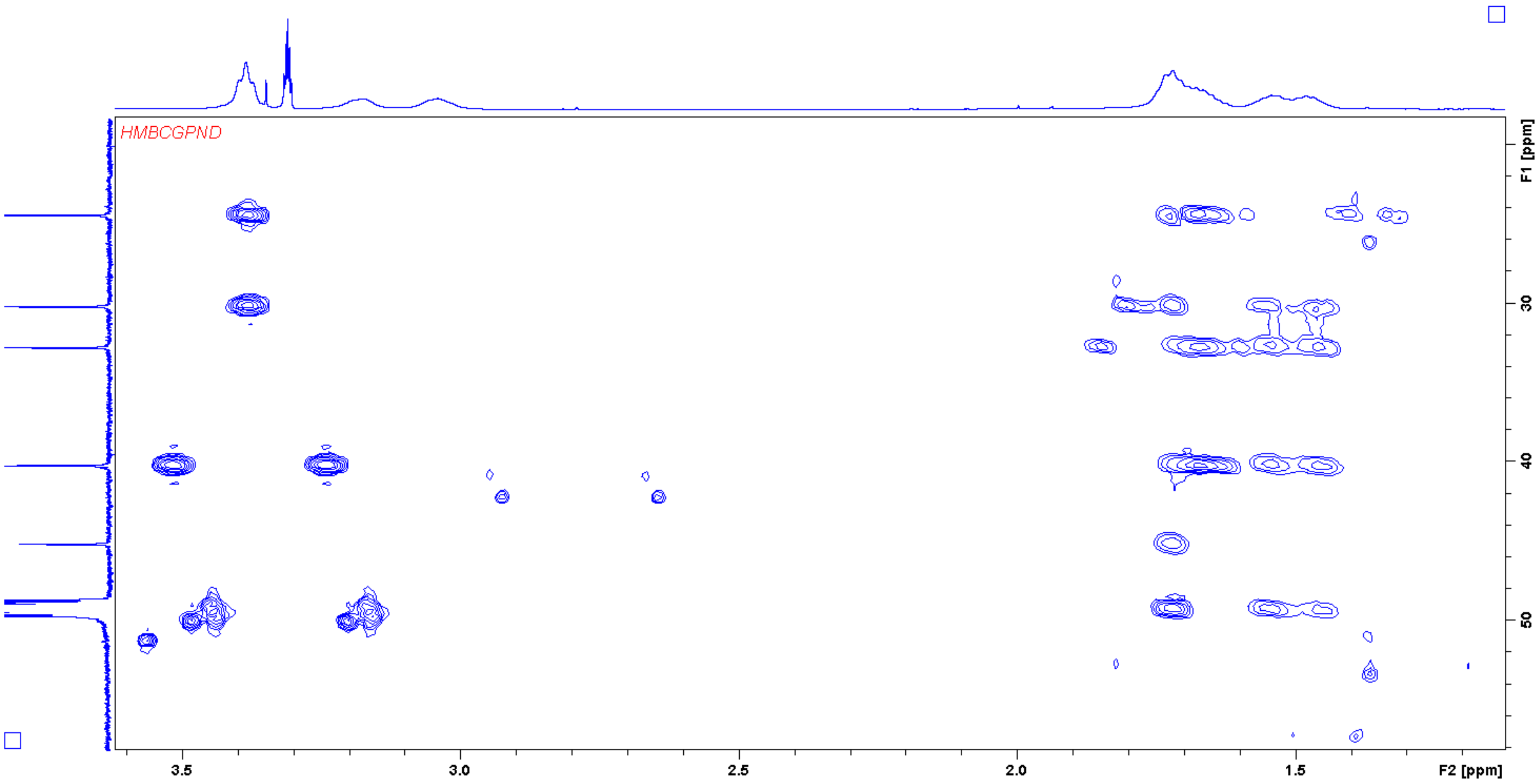

Figure S41. ^{1}H-^{13}C HMBC spectrum of compound **2** (expanded part 1).

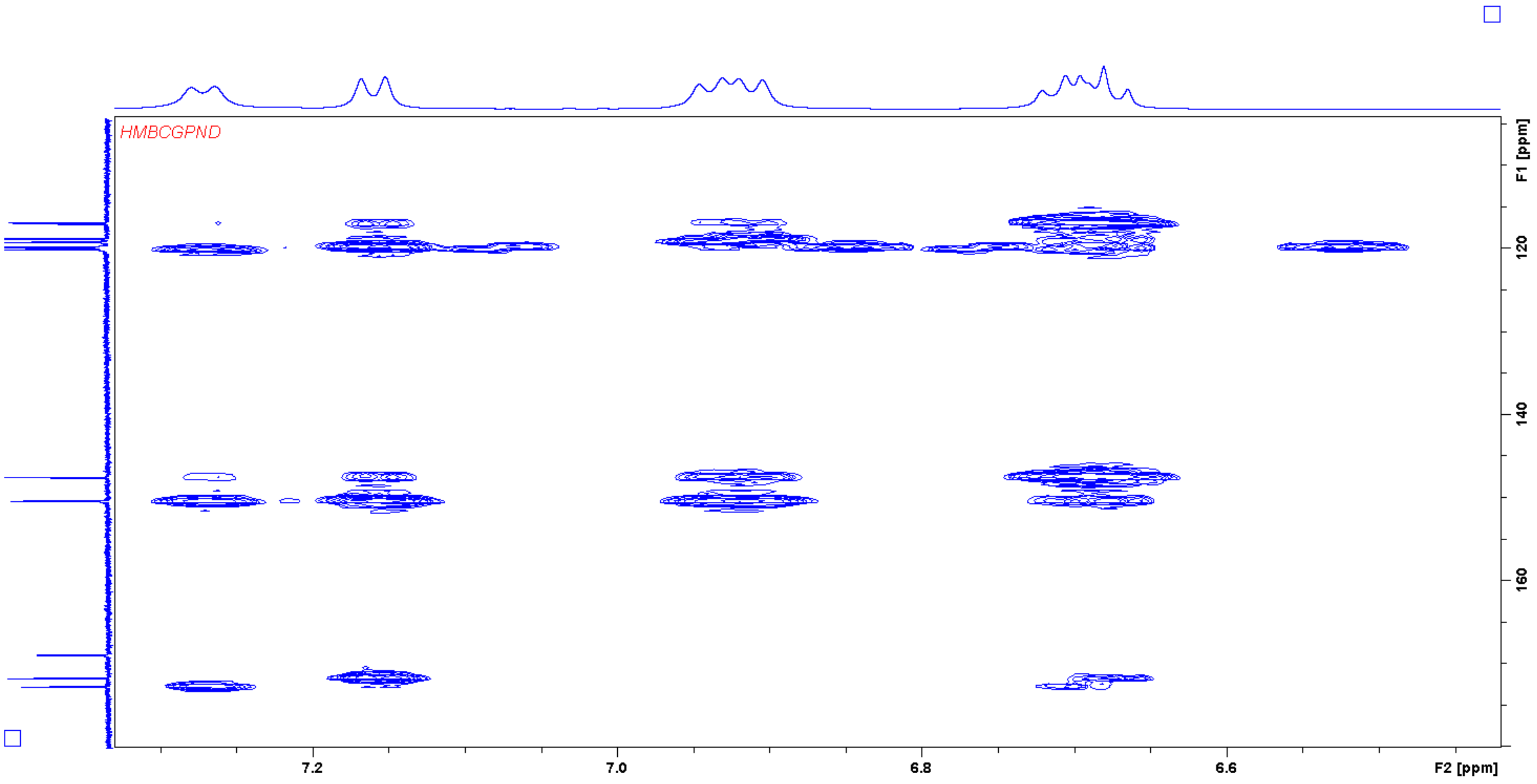

Figure S42. ^{1}H-^{13}C HMBC spectrum of compound **2** (expanded part 2).

microorganisms

MDPI

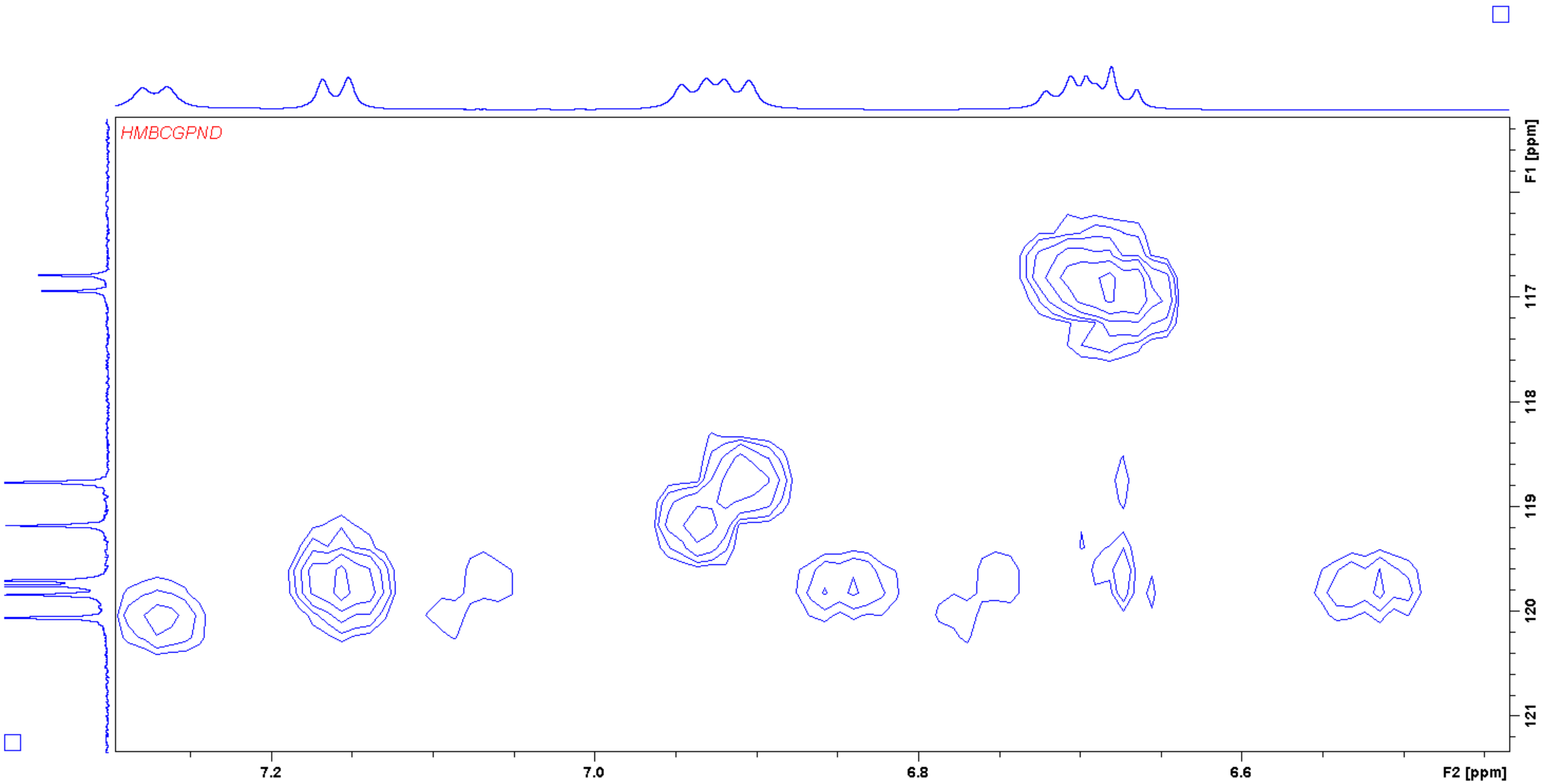

Figure S43. ^{1}H-^{13}C HMBC spectrum of compound **2** (expanded part 3).

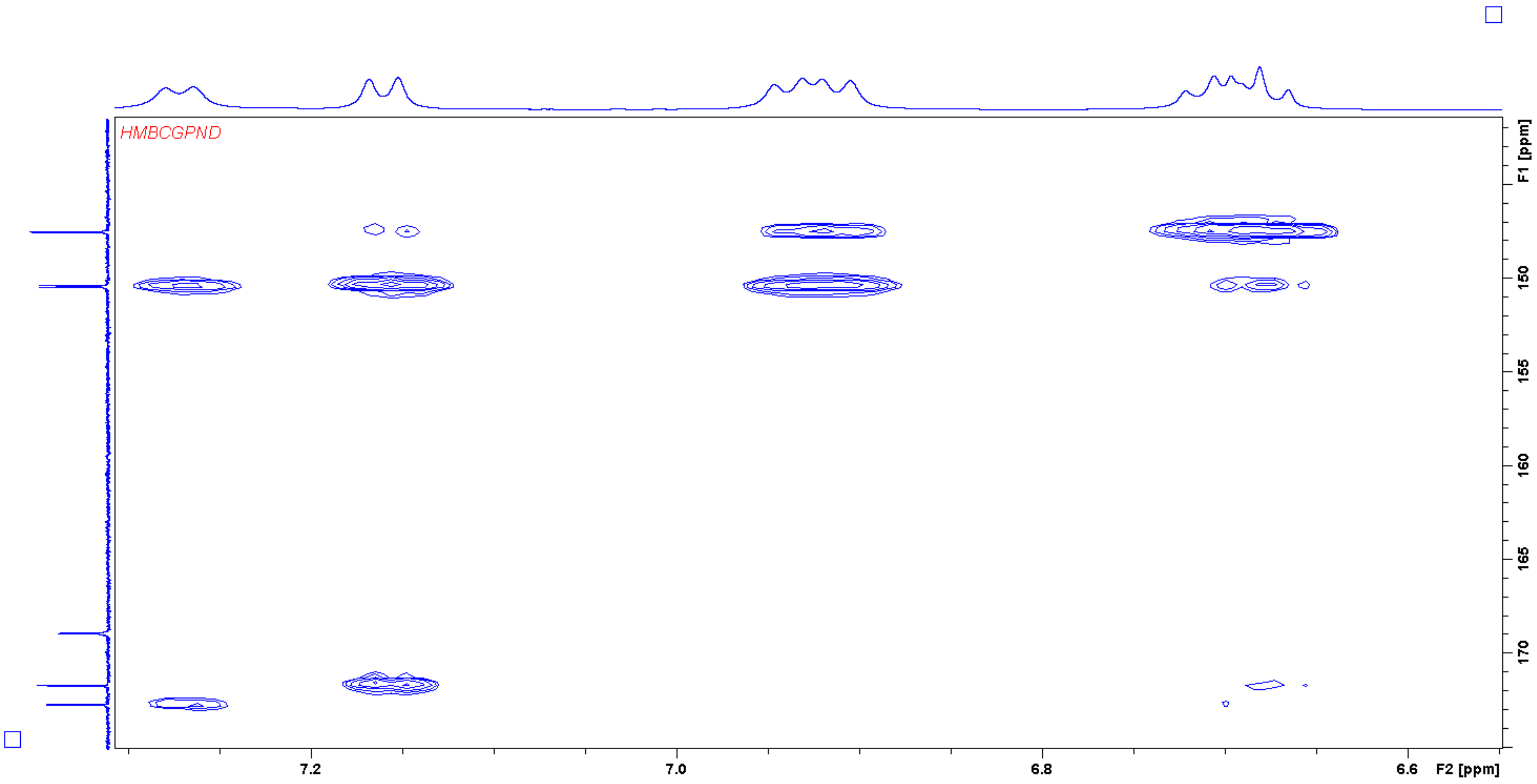

Figure S44. ^{1}H-^{13}C HMBC spectrum of compound **2** (expanded part 4).

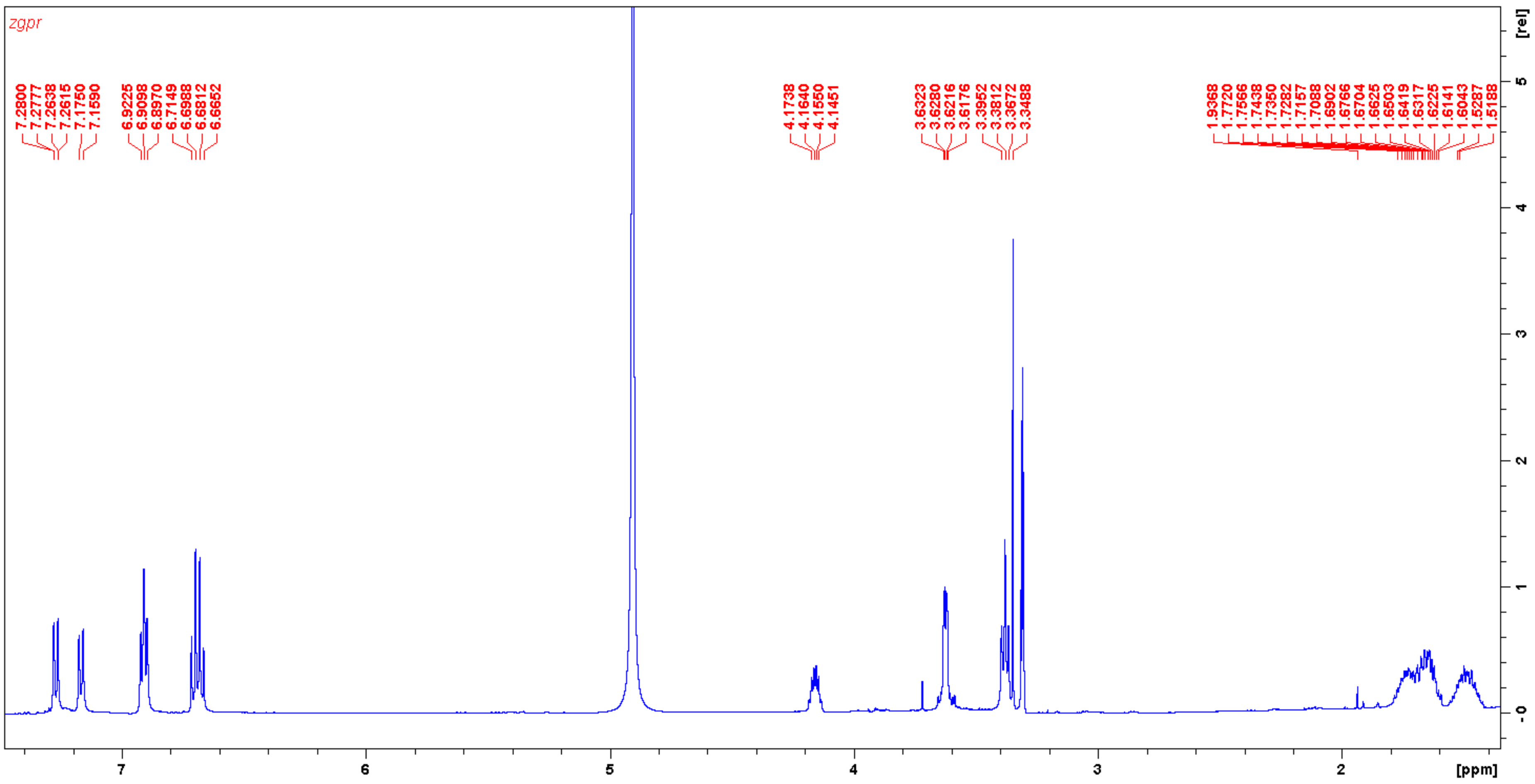

Figure S45: ¹H NMR spectrum of compound **3**.

microorganisms

MDPI

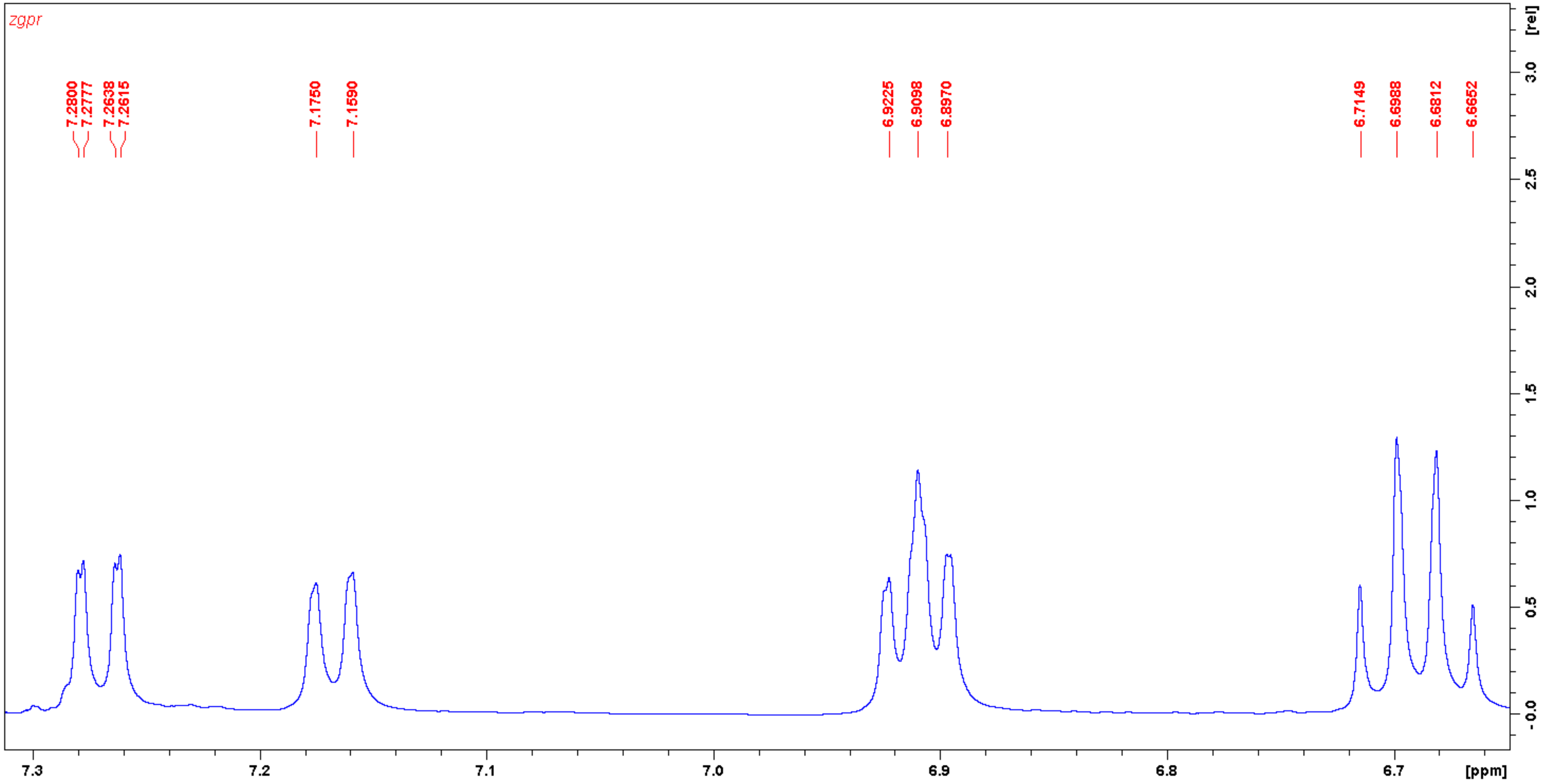

Figure S46. ^{1}H NMR spectrum of compound **3** (expanded part 1).

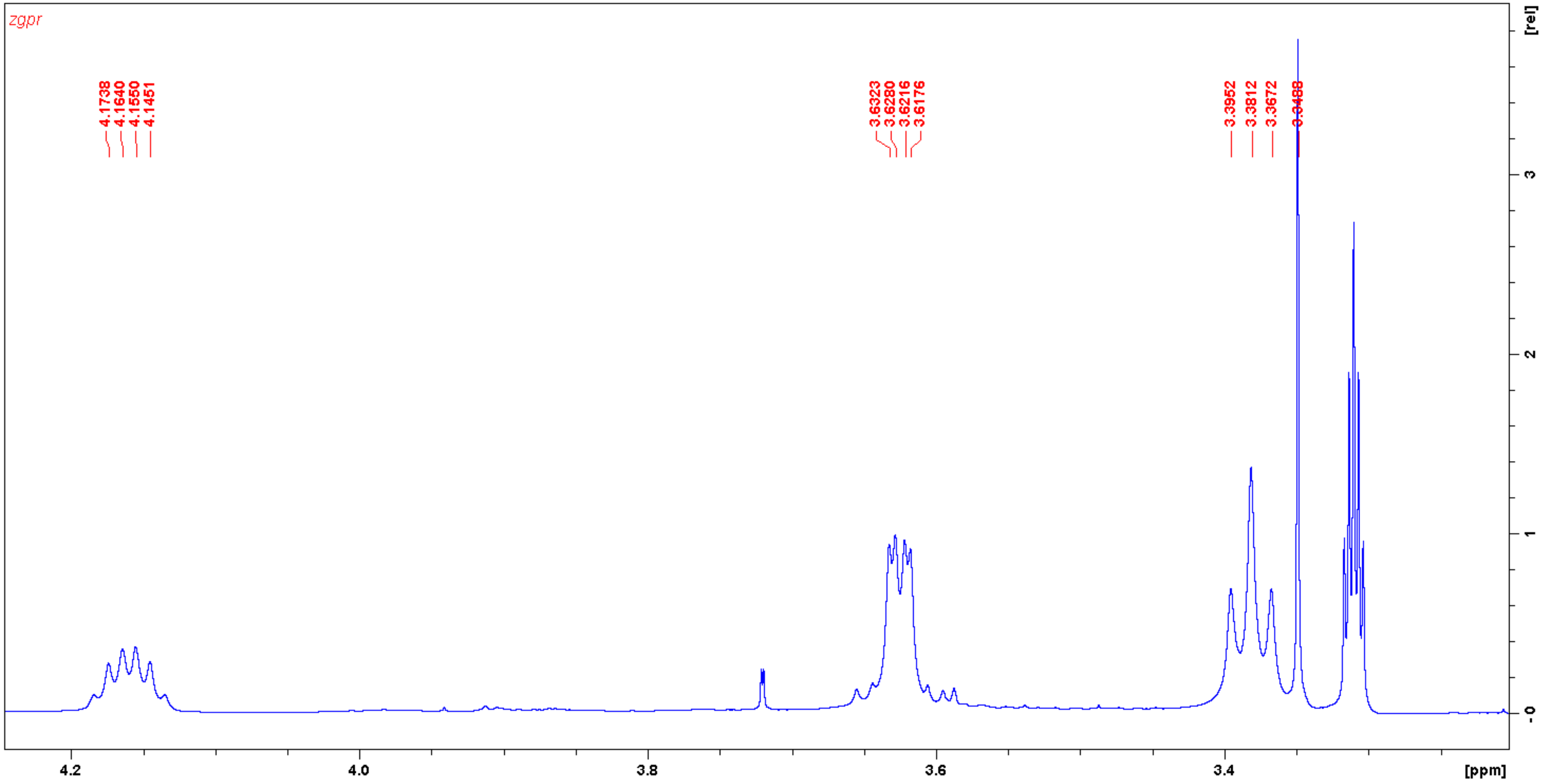

Figure S47. ^{1}H NMR spectrum of compound **3** (expanded part 2).

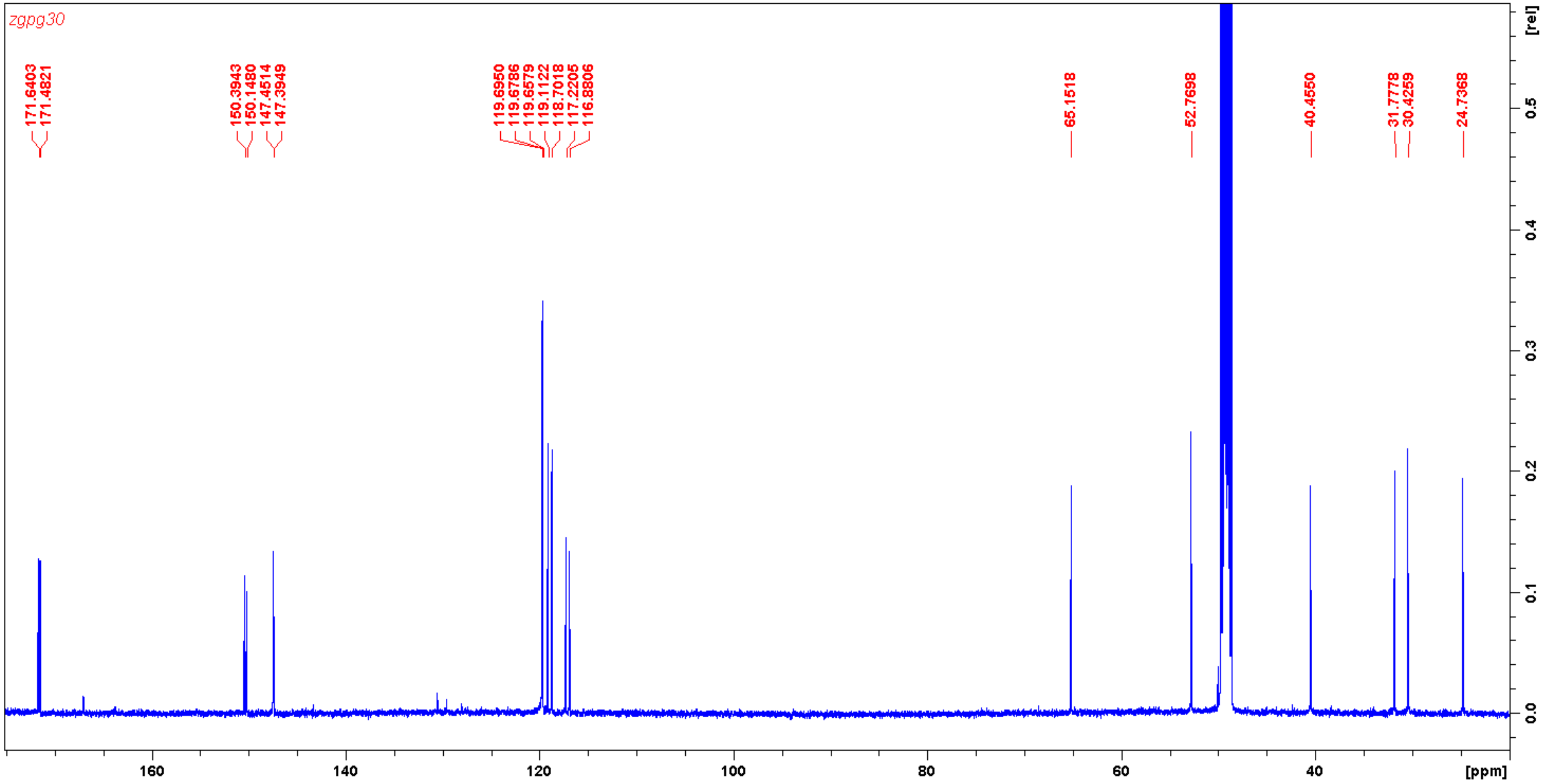

Figure S48. ^{13}C NMR spectrum of compound **3**.

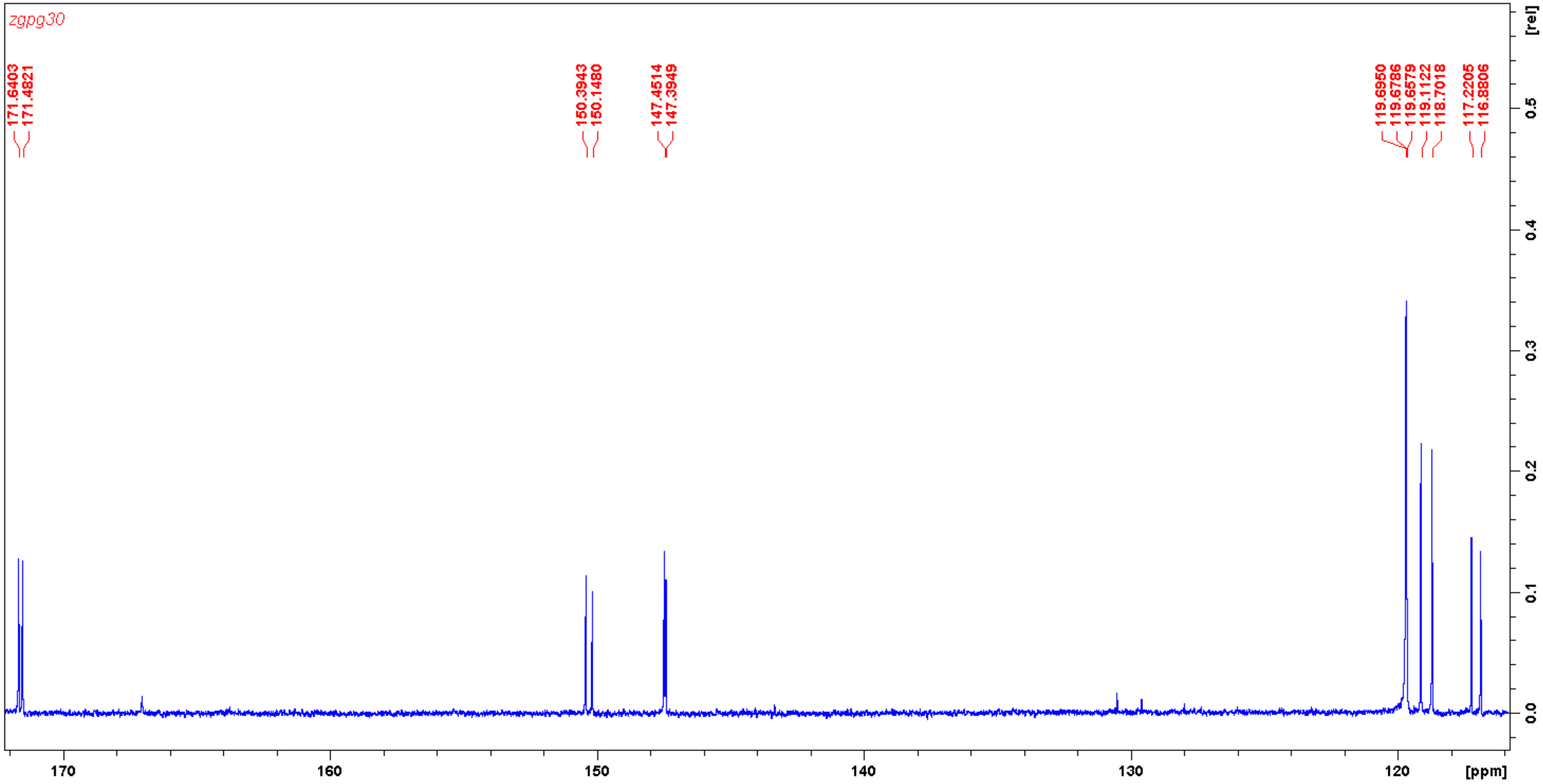

Figure S49. ^{13}C NMR spectrum of compound **3** (expanded part 1).

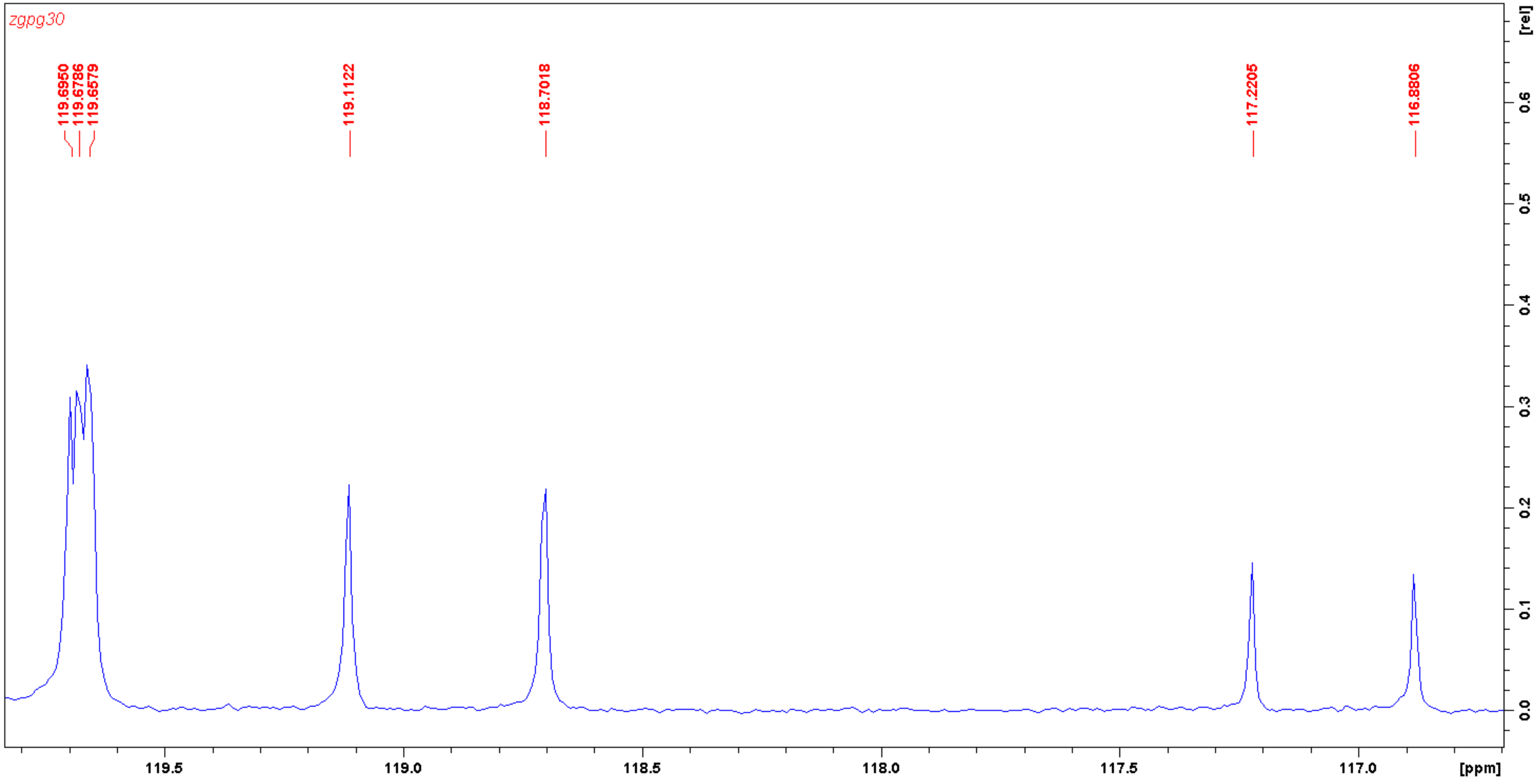

Figure S50. ^{13}C NMR spectrum of compound **3** (expanded part 2).

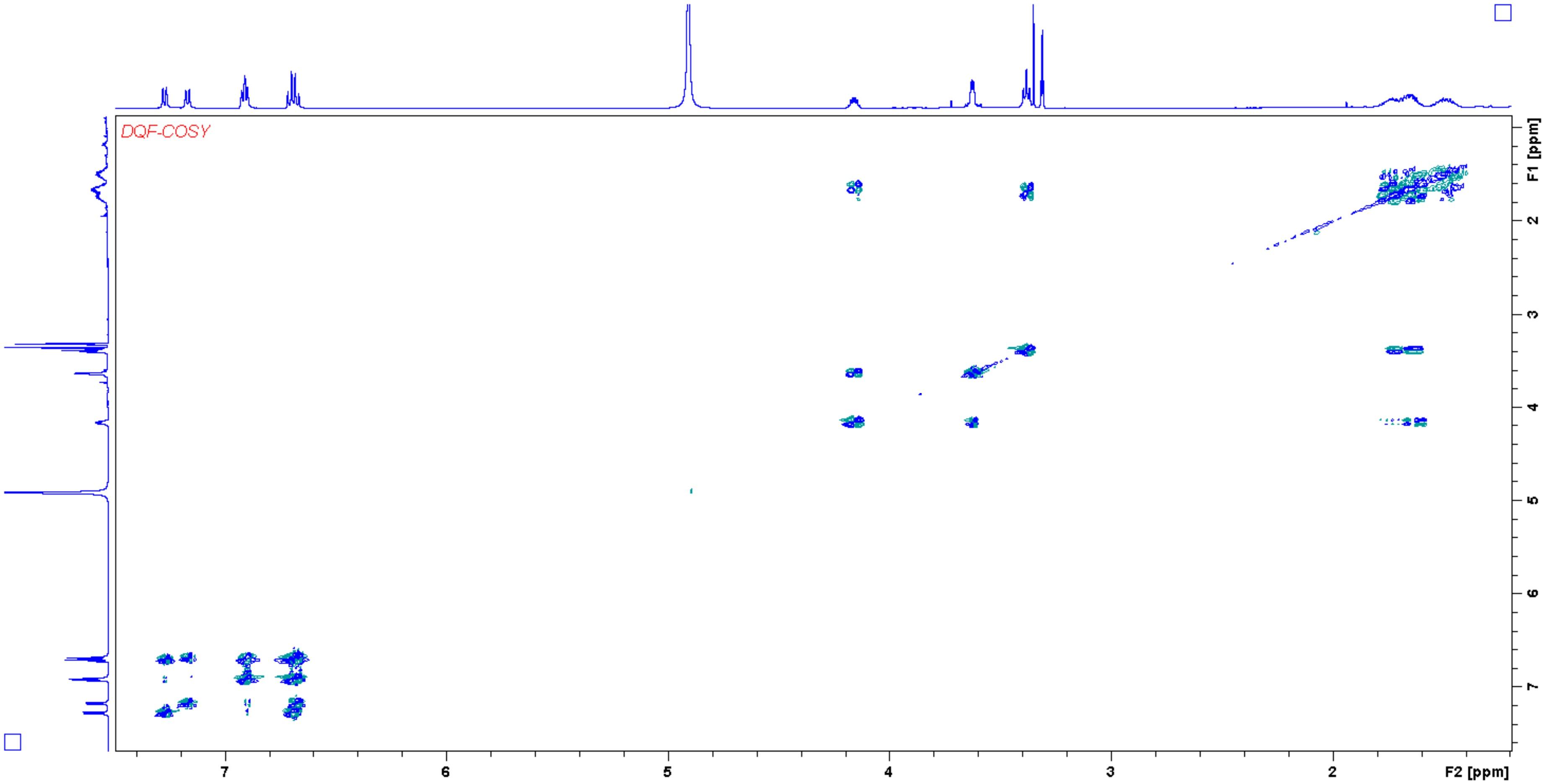

Figure S51. ^{1}H-^{1}H DQF-COSY spectrum of compound **3.**

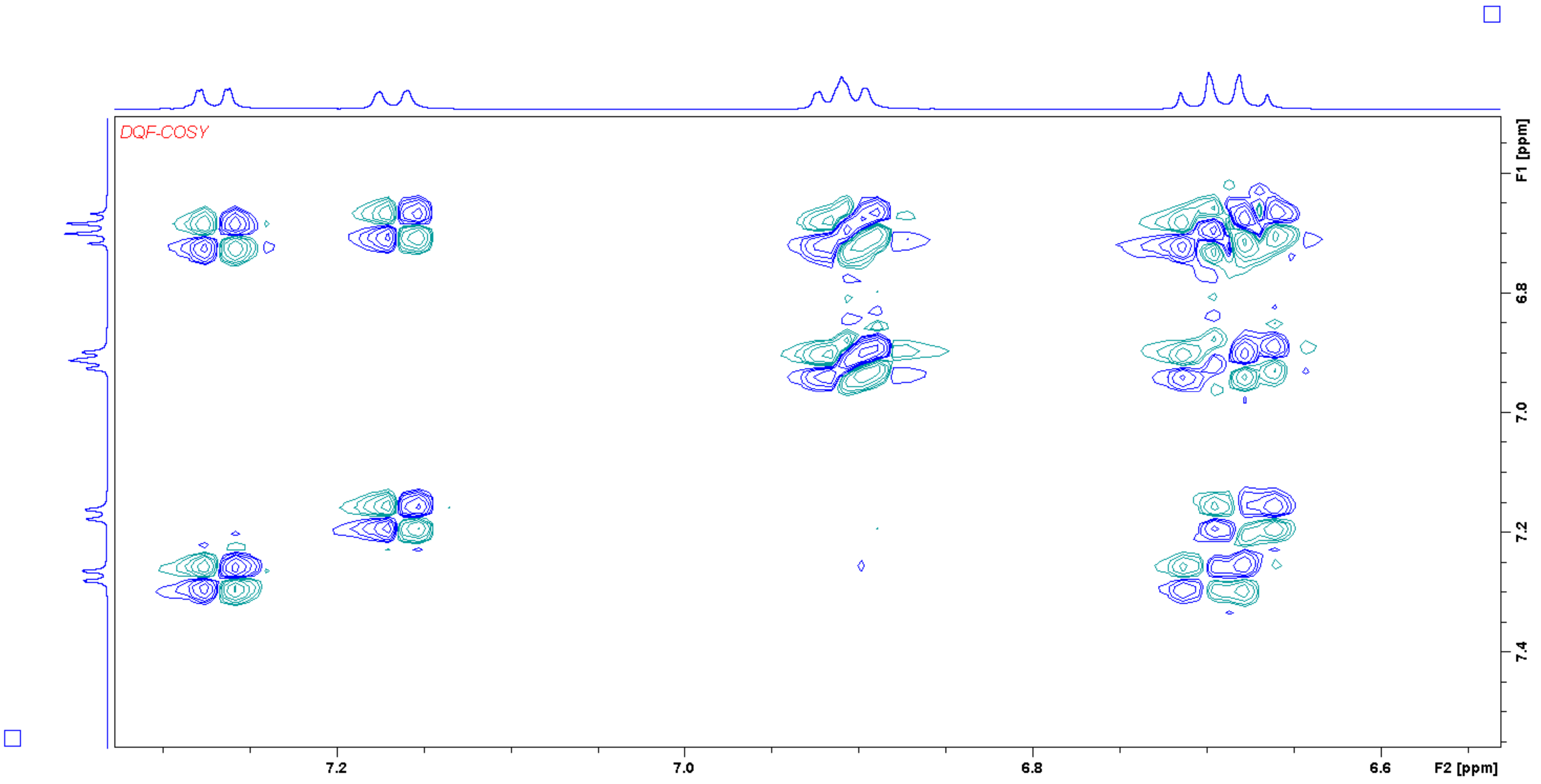

Figure S52. ^{1}H-^{1}H DQF-COSY spectrum of compound **3** (expanded part 1).

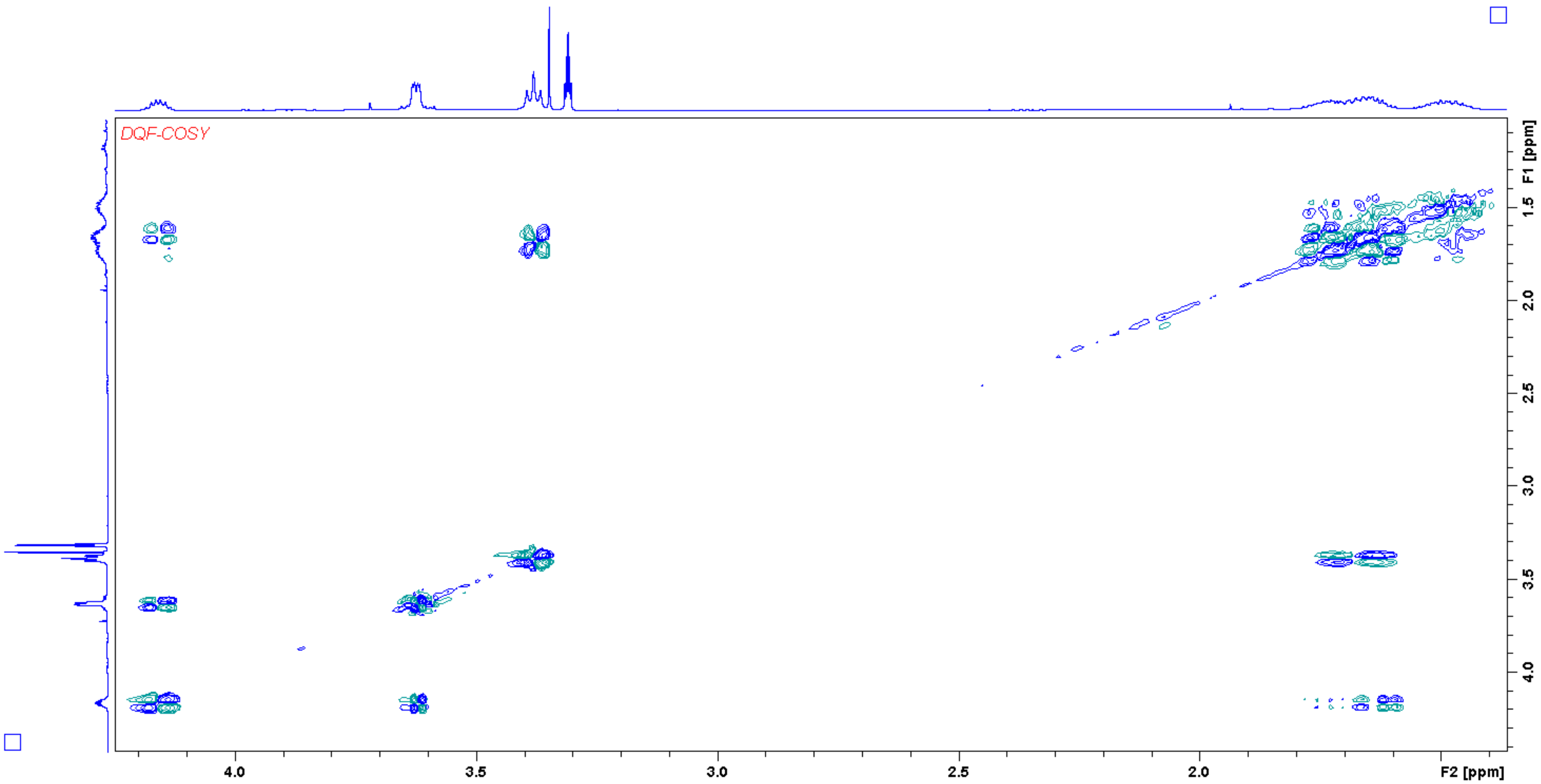

Figure S53. ^{1}H-^{1}H DQF-COSY spectrum of compound **3** (expanded part 2).

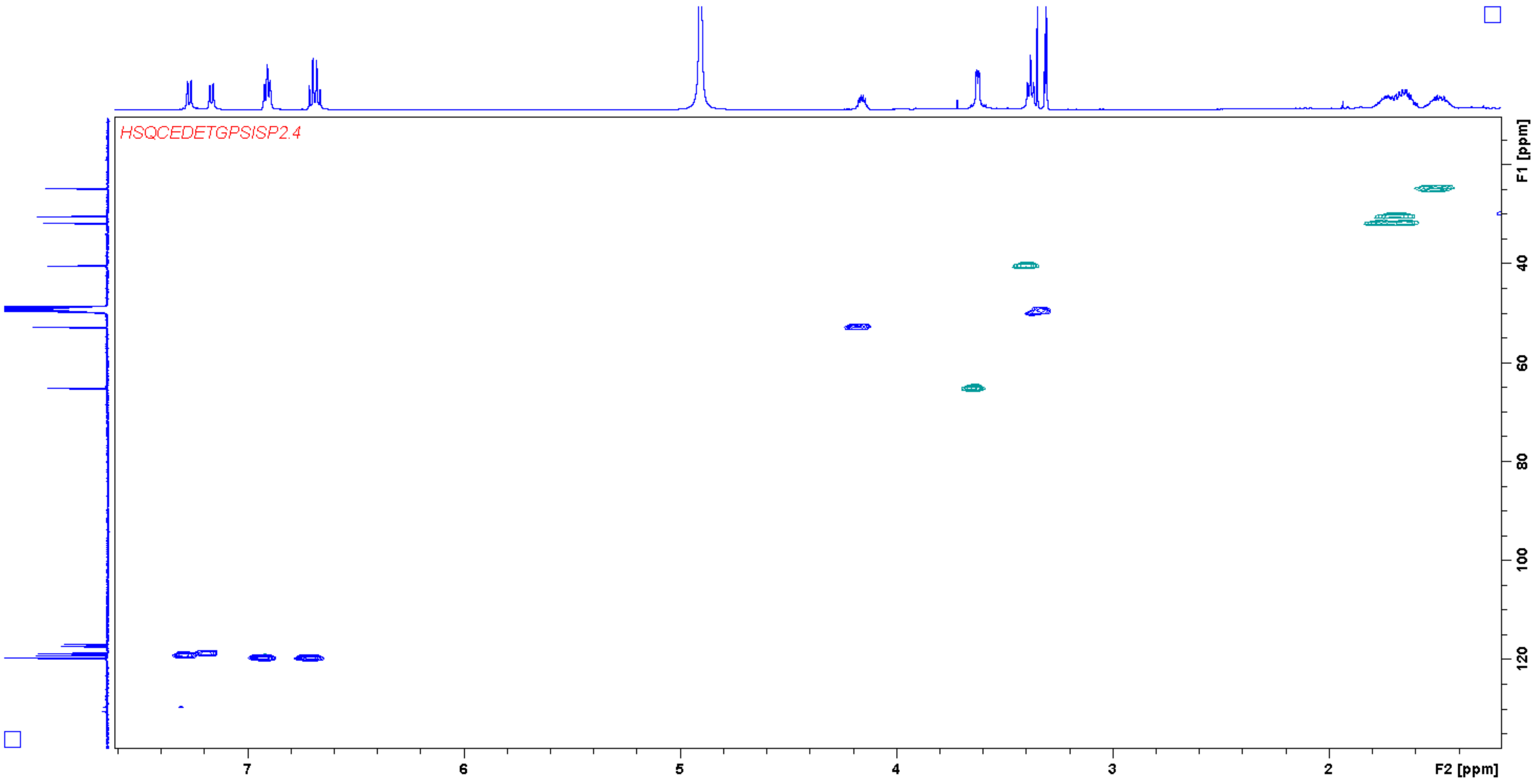

Figure S54. HSQC spectrum of compound **3**.

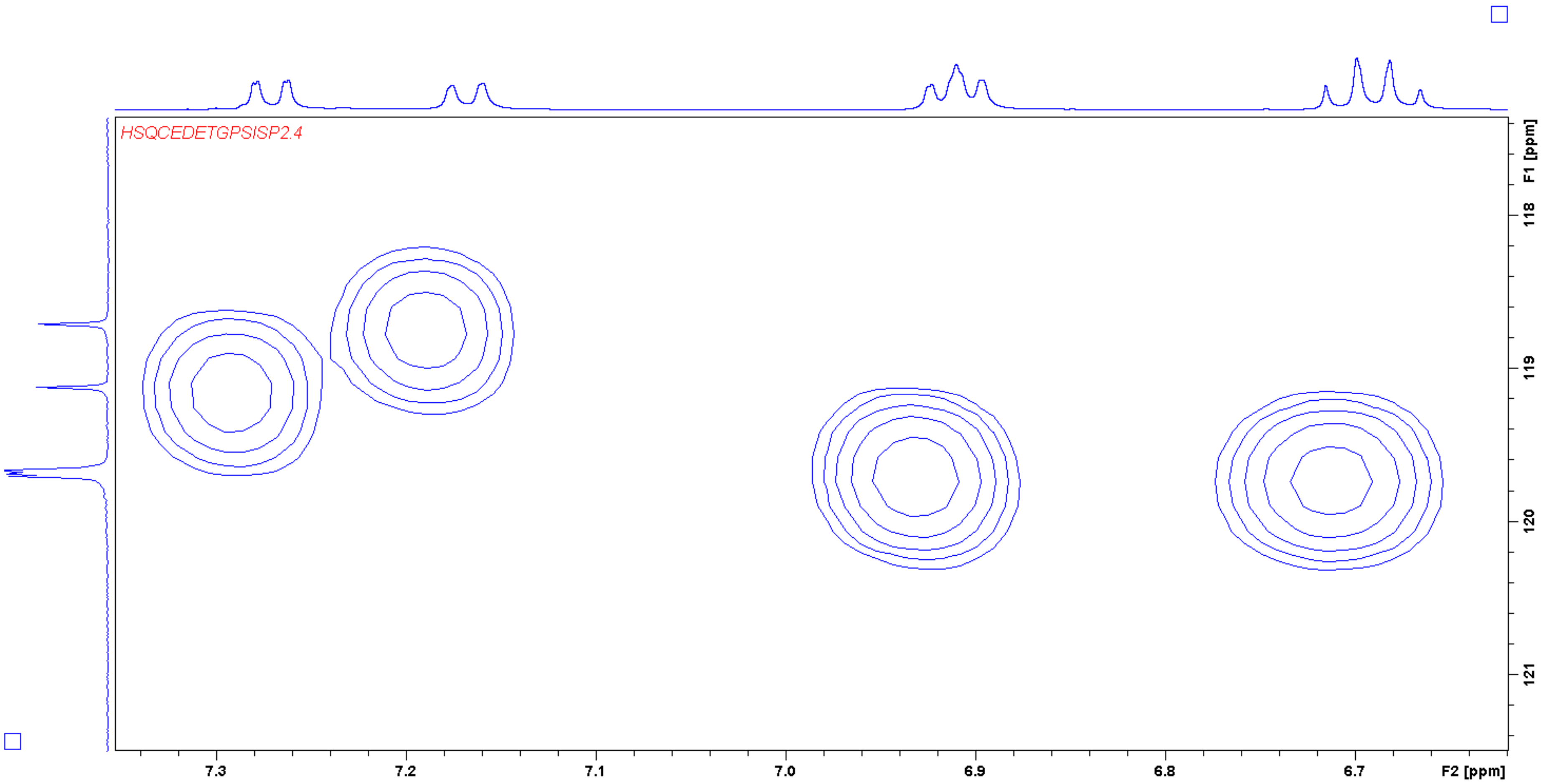

Figure S55. HSQC spectrum of compound **3** (expanded part 1).

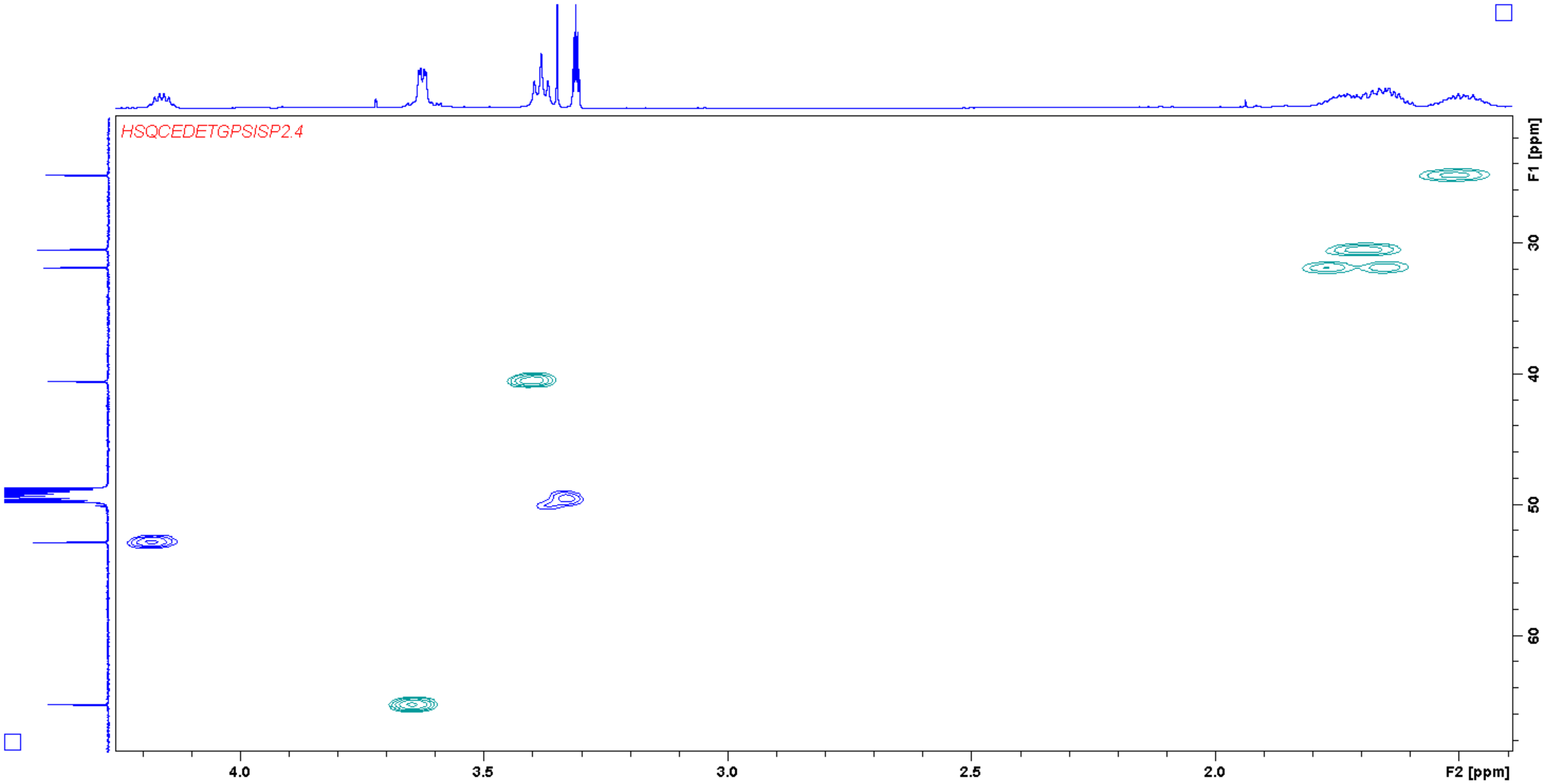

Figure S56. ^{1}H-^{1}H DQF-COSY spectrum of compound **3** (expanded part 2).

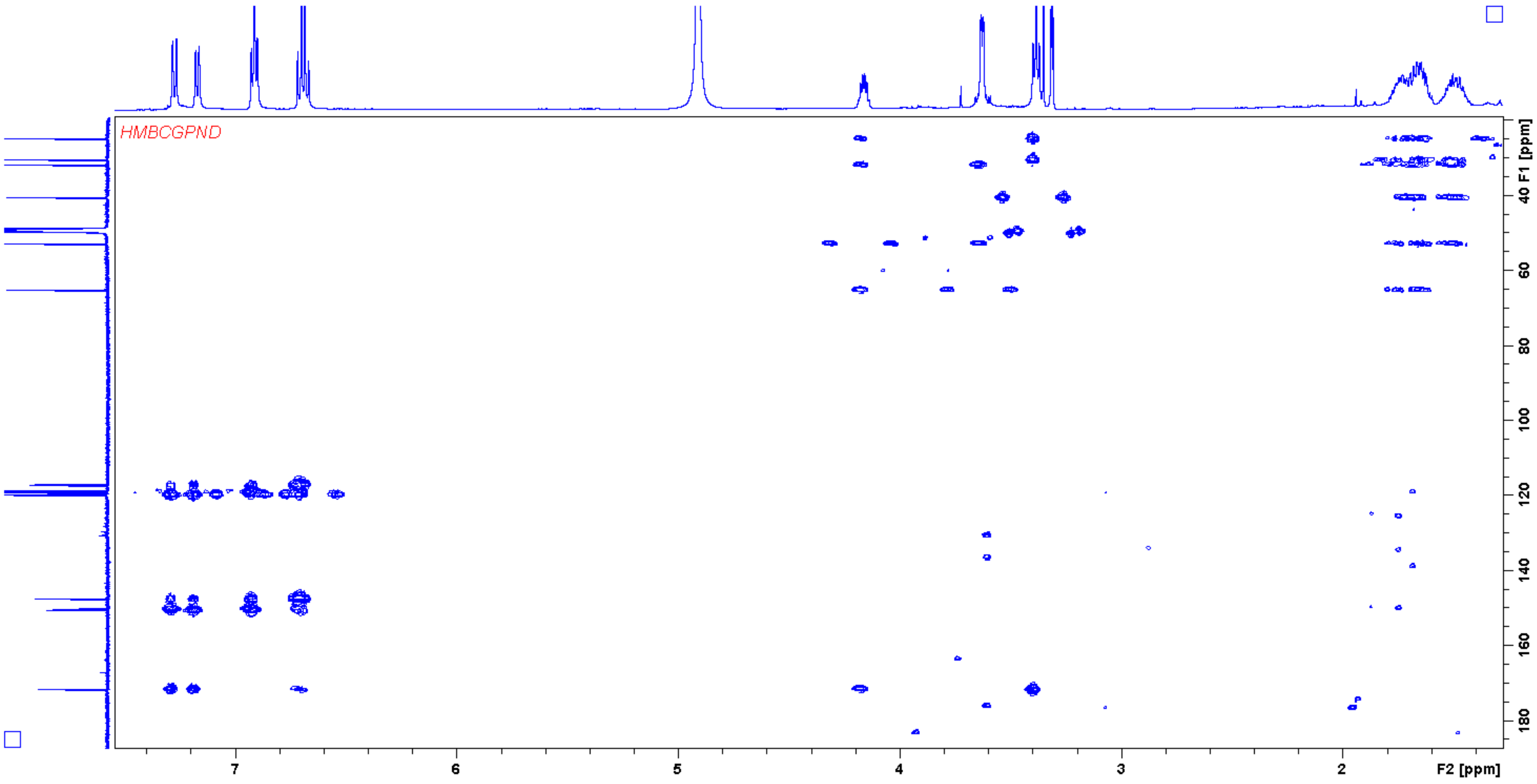

Figure S57. ^{1}H-^{13}C HMBC spectrum of compound **3**.

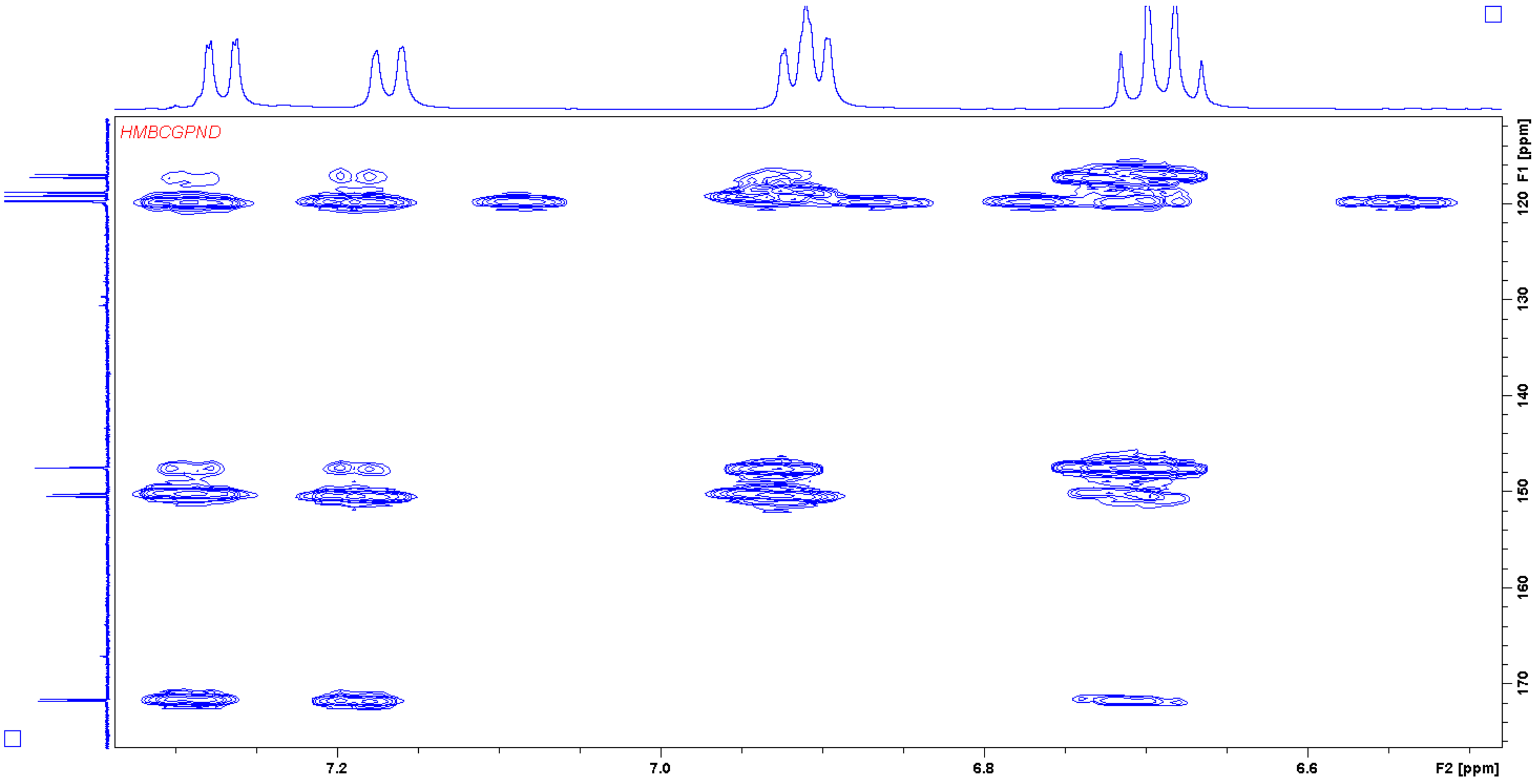

Figure S58. ^{1}H-^{13}C HMBC spectrum of compound **3** (expanded part 1).

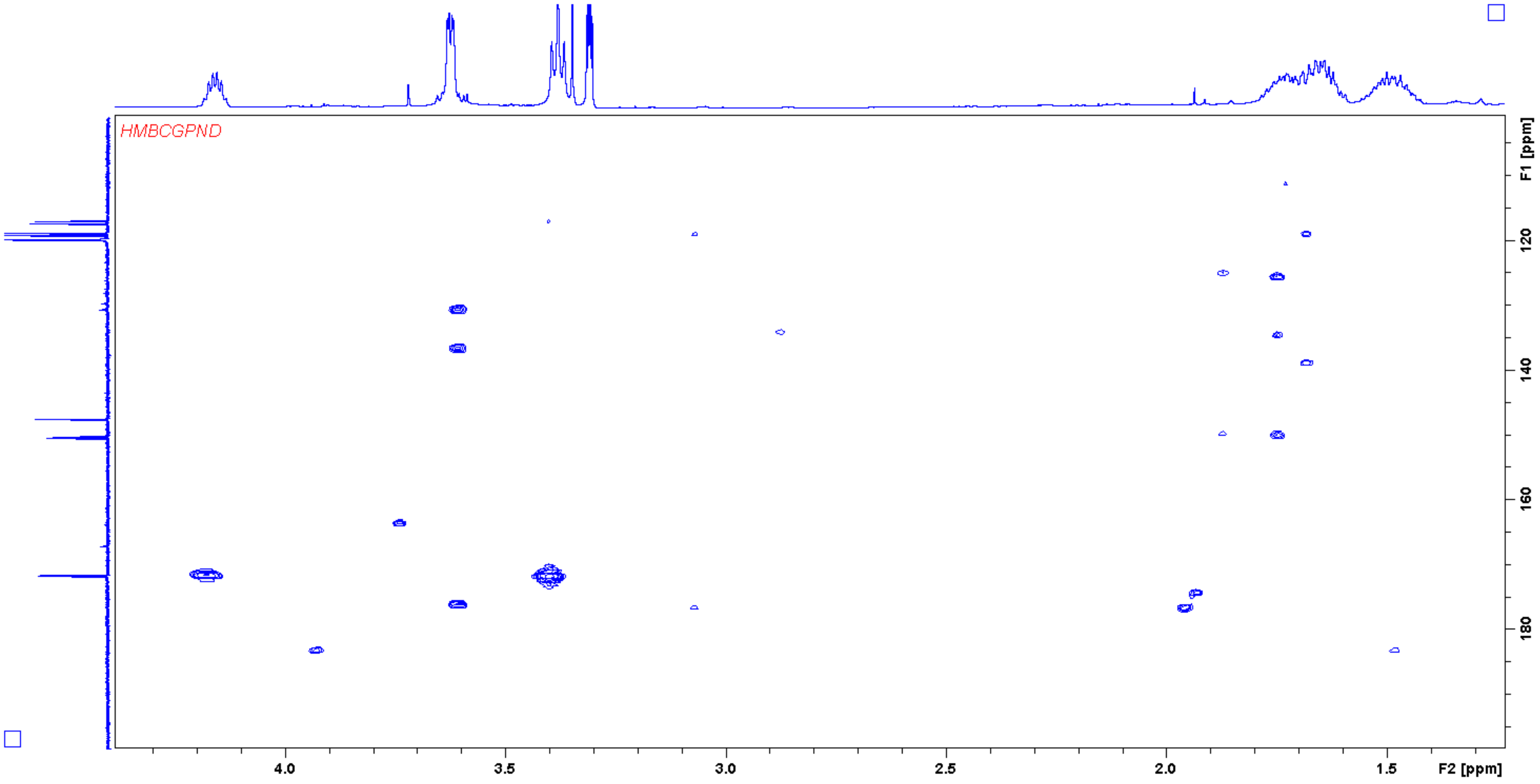

Figure S59. ^{1}H-^{13}C HMBC spectrum of compound **3** (expanded part 2).

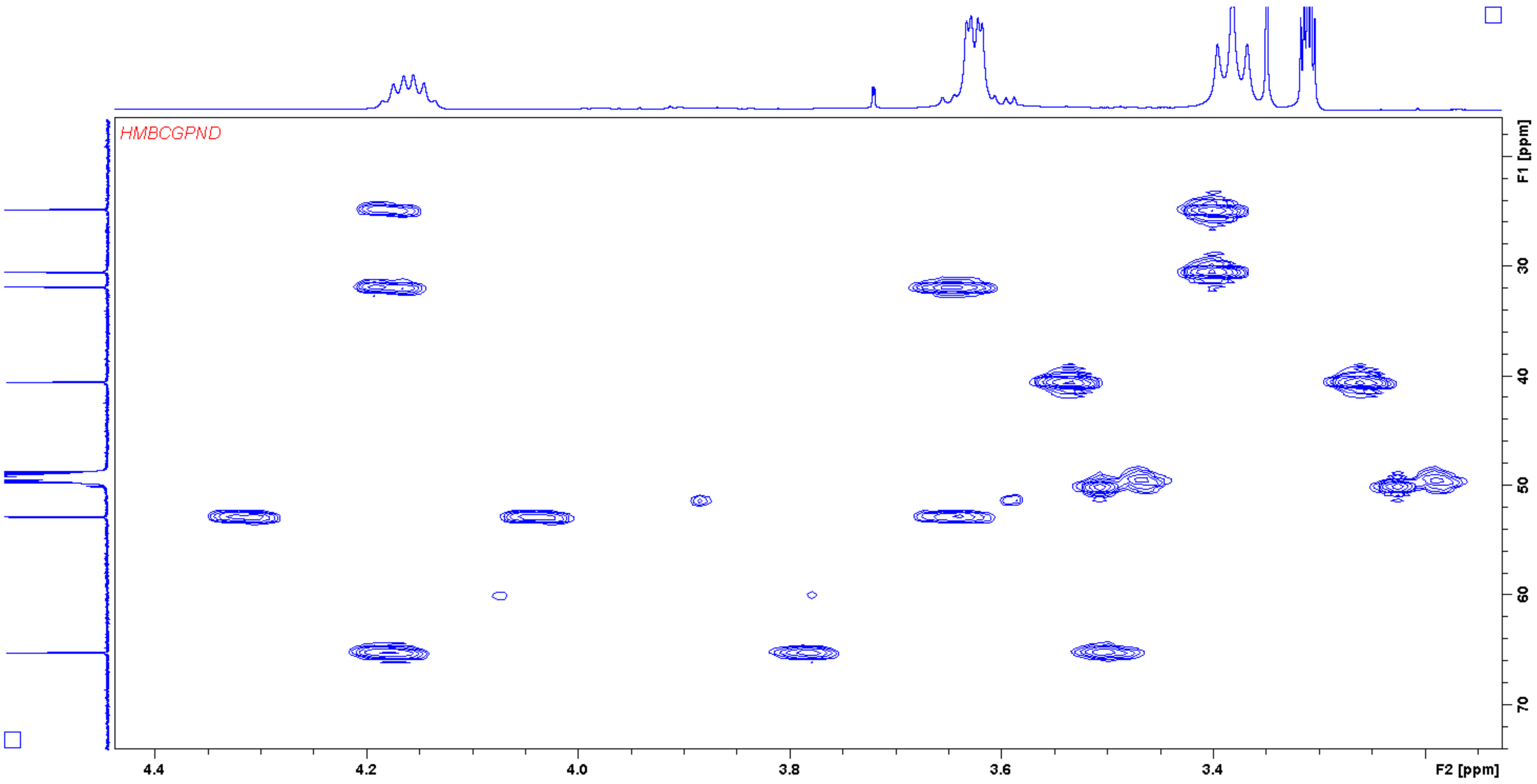

Figure S60. ^{1}H-^{13}C HMBC spectrum of compound **3** (expanded part 3).

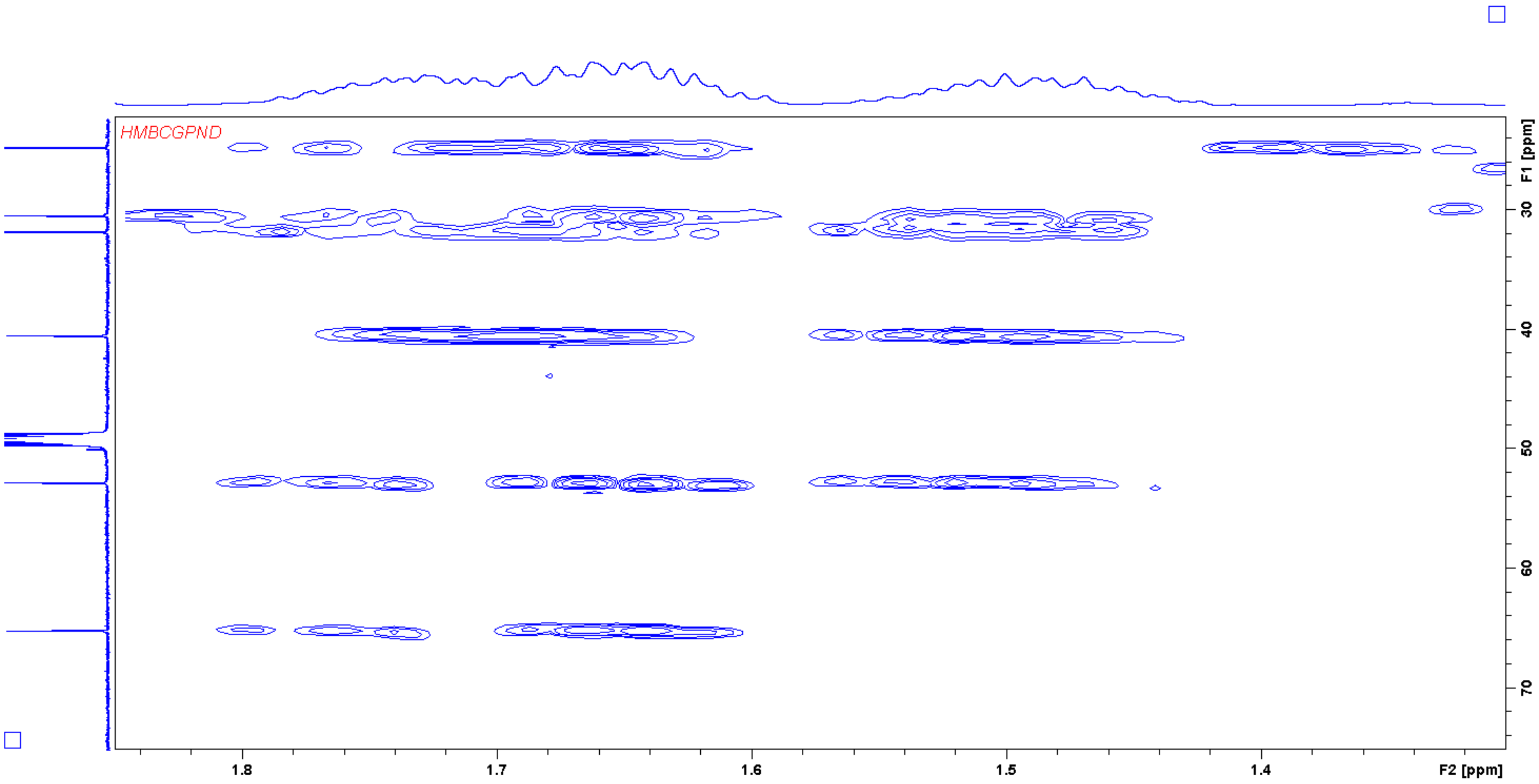

Figure S61. ^{1}H-^{13}C HMBC spectrum of compound **3** (expanded part 4).

6. References

1. Ambrosi, H.D.; Hartmann, V.; Pistorius, D.; Reissbrodt, R.; Trowitzsch-Kienast, W. Myxochelins B, C, D, E and F: A new structural principle for powerful siderophores imitating nature. *Eur. J. Org. Chem.* **1998**, 541–551.
